# Invited review: strategic adoption of antibiotic-free pork production: the importance of a holistic approach

**DOI:** 10.1093/tas/txac063

**Published:** 2022-05-16

**Authors:** John F Patience, Alejandro Ramirez

**Affiliations:** Department of Animal Science, Iowa State University, Ames, IA 50011, USA; Iowa Pork Industry Center, Iowa State University, Ames, IA 50011-1178, USA; College of Veterinary Medicine, University of Arizona, Oro Valley, AZ 85737, USA

**Keywords:** antimicrobial resistance, diet formulation, feed additives, no antibiotics ever, organic, raised without antibiotics

## Abstract

The discovery of the use of antibiotics to enhance growth in the 1950s proved to be one of the most dramatic and influential in the history of animal agriculture. Antibiotics have served animal agriculture, as well as human and animal medicine, well for more than seven decades, but emerging from this tremendous success has been the phenomenon of antimicrobial resistance. Consequently, human medicine and animal agriculture are being called upon, through legislation and/or marketplace demands, to reduce or eliminate antibiotics as growth promotants and even as therapeutics. As explained in this review, adoption of antibiotic-free (**ABF**) pork production would represent a sea change. By identifying key areas requiring attention, the clear message of this review is that success with ABF production, also referred to as “no antibiotics ever,” demands a multifaceted and multidisciplinary approach. Too frequently, the topic has been approached in a piecemeal fashion by considering only one aspect of production, such as the use of certain feed additives or the adjustment in health management. Based on the literature and on practical experience, a more holistic approach is essential. It will require the modification of diet formulations to not only provide essential nutrients and energy, but to also maximize the effectiveness of normal immunological and physiological capabilities that support good health. It must also include the selection of effective non-antibiotic feed additives along with functional ingredients that have been shown to improve the utility and architecture of the gastrointestinal tract, to improve the microbiome, and to support the immune system. This holistic approach will require refining animal management strategies, including selection for more robust genetics, greater focus on care during the particularly sensitive perinatal and post-weaning periods, and practices that minimize social and environmental stressors. A clear strategy is needed to reduce pathogen load in the barn, such as greater emphasis on hygiene and biosecurity, adoption of a strategic vaccine program and the universal adoption of all-in-all-out housing. Of course, overall health management of the herd, as well as the details of animal flows, cannot be ignored. These management areas will support the basic biology of the pig in avoiding or, where necessary, overcoming pathogen challenges without the need for antibiotics, or at least with reduced usage.

## Teaser

Adoption of antibiotic-free pork production is a very demanding strategy that requires a holistic approach to achieve success. This will include selecting effective non-antibiotic feed additives, modifying diet formulations to support normal immunological and physiology mechanisms, minimizing physical, social, and environmental stressors, and enhancing hygiene, biosecurity, and disease management.

## INTRODUCTION AND MOTIVATION

The potential to use antibiotics as growth promotants in animal agriculture was first discovered in the late 1940s and early 1950s. Moore et al. (1946) may have been the first to report the growth-promoting effects of antibiotics when they observed unexpected increases in growth rates in chicks when either sulfasuxidine or streptomycin was included in their diet; interestingly, they were studying vitamin requirements and toxicity when they made what proved to be a critically important discovery that would alter the feeding of chickens for many decades into the future. Similarly, Jukes et al. (1950) reported that aureomycin improved pig growth performance, but only when the “animal protein factor” or vitamin B_12_ was present in the diet. Similar to the Moore study, little did these authors appreciate at the time the enormous impact of their findings.

Sub-therapeutic levels of antibiotics earned widespread use in swine feeds initially to 1) improve product quality through reduced fat and increased lean content, 2) improve rate and efficiency of gain, and 3) control pathogens such as *Salmonella*, *Campylobacter*, *Escherichia coli,* and *enterococci*, resulting in healthier animals performing at a higher level (Cromwell, 2002; Hughes and Heritage, 2004). The benefit of improved carcass traits became less valuable over time as greater progress could be achieved through genetic selection combined with improved diet formulation.

Cromwell (2002) summarized the results of 453 experiments evaluating the use of in-feed antibiotics in the starter phase of pork production, 298 experiments in the growing phase, and 443 experiments in the growing-finishing phase, involving 13,632, 5,783, and 13,140 pigs, respectively. As expected, the benefit was found to be greatest in the younger pig, but declined as the pigs grew older. Thus, average daily gain and feed efficiency increased by an average of 16.4% and 6.9%, respectively, in the starter phase but only by 4.2% and 2.2%, respectively, in the growing-finishing phase.

According to Teillant and Laxminarayan (2015), who summarized the results of studies conducted between 1950 and 2005, there has been a substantial decline in the magnitude of the response to antibiotic growth promotants, especially in the grow-finish phase of production, although there is no consensus why this is occurring. It may be due to improvements in one or a combination of animal management and health care, improved nutrition, more robust genetics or vastly improved housing conditions compared to those used 50 yr ago. The one flaw in this analysis is the fact that only one study on the response to antibiotic growth promotants during the nursery period was reported (Dritz et al., 2002). More recently, Ruckman et al. (2020) reported a 4% improvement in ADG overall, but the increase was 9% when less complex diets were employed; this study was conducted during the starter phase of production on a commercial research farm.

While antibiotics continue to be used for the prevention (prophylaxis), control (metaphylaxis), and treatment of specific pathogens, the quantity of antibiotics used by the pig industry in the United States was decreasing 2 yr prior to federal regulatory controls, which were implemented on January 1, 2017 (FDA, 2018). Notably, the greater decline in usage was particularly pronounced in antimicrobials considered to be important in human medicine (FDA, 2018). In many parts of the world, antibiotics that play a role in human medicine can no longer be used for growth promotion.

In addition to regulatory or legislative demand, there has been increasing expectation in the consumer marketplace for pork derived from pigs that have been raised without the use of antibiotics or at least reduced use of antibiotics (Denver et al., 2021). Globally, antibiotic-free (**ABF**) production has captured significant portions of the broiler and dairy markets, but it has proven to be much more challenging with pigs. Importantly, there is reason to believe that consumers are unaware that excessive restrictions on the use of antibiotics could negatively impact pig welfare (Karavolias et al., 2018; Lemos Teixeira et al., 2021).

ABF was the first term applied in pork production, signifying the absence of antibiotics; the term is still frequently used. However, more recent terms have been suggested, since they are viewed as somewhat more definitive: “raised without antibiotics” and with “no antibiotics ever” are two of the more common examples. They are preferred by some because they appear to be more definitive than the term ABF, which sometimes means different things to different people.

Indeed, defining ABF is no simple task. Most people have defined ABF as no antibiotics provided to the pig through feed, water, or injection during the full course of its life. Others have defined it as no antibiotic provided to the pig in any manner “after weaning.” Still others suggest that ABF means no antibiotics can be used in pork production if they are important in human medicine. The Canadian Food Inspection Agency (CFIA, 2018) has adopted a stringent definition of ABF: the animal may not have been treated with antibiotics administered by any method, from birth to slaughter or harvest. Furthermore, antibiotics may not be administered to the lactating mother in any manner that would result in antibiotic residue in the offspring. Clearly, the term ABF does not have the same meaning in all instances.

The term ABF in the consumer milieu creates additional confusion because markets tend to impose other concurrent demands on the pork production system; the most common is freedom from all forms of animal protein, or even all animal products in their diets. Sometimes, “ABF” animals must be raised in housing conditions with specific restrictions, such as raised outdoors, the use of bedding or open pen gestation.

On October 25, 2018, the European Union announced further restrictions on the use of antibiotics in livestock production, noting that the preventative use of antibiotics will be restricted to individual animals, and then only when a veterinarian believes the risk of infection is very high. Treatment of a group of animals will only be permitted when all other alternatives have been exhausted. At the time of the announcement, the new rules were expected to reserve certain antimicrobials for exclusively human use. These changes were to take effect in 2022.

The terms “antibiotic” and “antimicrobial” are sometimes incorrectly applied in both science and general conversation. The former is a substance that is derived from microorganisms which at low concentrations has the ability to inhibit or destroy other microorganisms, while the latter is a broader term embracing any substance of natural or synthetic origin which may inhibit or kill microorganisms. Examples of both will be discussed later in this review. There are many products with antimicrobial properties that can and are used in ABF production systems, but they cannot be antibiotics.

The objective of this manuscript is to identify, in a single document, nutritional and management strategies, as well as select feed additives, to support success in ABF pork production. This hopefully emphasizes that a multidisciplinary approach, which effectively addresses the whole pork production system, is required; certainly, it requires more than simply selecting a feed additive or additives to replace antibiotics. Success in ABF production will require changes in how diets are formulated, in the use of carefully selected feed additives, and in the management of the pig, through the adoption of robust genetics, enhanced perinatal and post-weaning animal care, minimized social and other stressors, reduced pathogen load in the barn, and the adoption of improved health management systems.

## BIOLOGICAL BASIS FOR ANTIBIOTIC-DRIVEN GROWTH PROMOTION

Despite having been used for more than half a century, the exact mode of action of antibiotic growth promotants is not well understood. Numerous possibilities exist, although most explanations focus on their impact on the microbiome, supported by the observation that they fail to show benefit in germ-free animals (Visek, 1978). It is widely accepted that antibiotic growth promotants provide bactericidal, bacteriostatic, or antiprotozoal effects in the gut (Thomke and Elwinger, 1998), thereby 1) stimulating nutrient absorption as a consequence of a thinner intestinal wall, known to exist in pigs treated with some antibiotic growth promotants, 2) reducing use of nutrients by the microbiota themselves, 3) reducing microbial metabolites such as ammonia and amines with known growth-depressing effects, 4) inhibiting infection, which in turn reduces the metabolic cost of an activated innate immune system, or 5) reducing opportunistic pathogens which cause subclinical infection and associated pathologies (Visek, 1978; Dibner and Richards, 2005; Niewold, 2007).

An activated immune system places a significant burden on available nutrient and energy supply; for example, Huntley et al. (2018) reported that stimulation of the immune system through administration of lipopolysaccharide (a common and highly immunogenic component of cell walls from Gram-negative bacteria) increased the maintenance energy requirement of young pigs by 23.3% and reduced growth rate by 18.3%. Furthermore, cytokines are known to impair appetite as well as release catabolic hormones, which in turn slow protein accretion.

Other theories exist with respect to the mode of action of antibiotic growth promotants. Niewold (2007) proposed that many antibiotics accumulate in inflammatory cells and are able to stimulate their bactericidal function while at the same time inhibiting parts of the innate immune response, namely that which is associated with macrophages and polymorphonucleocytes. Biancone et al. (2002) explains that the intestine is a critical organ supporting a highly responsive immune system, but that over-stimulation can lead to reduced productivity. For example, when cytokines are released, the liver switches to production of acute-phase proteins and away from normal protein synthesis; catabolism of muscle also takes place along with anorexia (Niewold, 2007). This is a very expensive response by the immune system (Humphrey and Klasing, 2003); if an antibiotic growth promotant suppresses this response, improved productivity could very well be the consequence.

## ANTIMICROBIAL RESISTANCE

There is little question that the use of antibiotics has been a boon for pork producers, due to their ability to reduce mortality, and improve animal health, care, and well-being, resulting in a lower of the cost of food production. However, controversy has arisen due to the development of antimicrobial resistance. Bacteria may become resistant to antimicrobials by spontaneous genetic mutation or by horizontal gene transmission (Sneeringer et al., 2019). Starr and Reynolds (1951) are believed to be the first to report on antimicrobial resistance in animal agriculture, in this instance in turkeys.

The most frequently cited example of antimicrobial resistance in human medicine is the methicillin-resistant *Staphylococcus aureus* (Lassok et al., 2013). Such strains are typically also resistant to erythromycin, clindamycin, tetracycline, and members of a class of antibiotics known as aminoglycosides. The evolution of antimicrobial-resistant pathogens is considered a matter of grave concern in human medicine. In 2013, the Centers for Disease Control identified 18 bacterial infections of great concern due to the rising development of antimicrobial-resistant infections (CDC, 2013). Three of these were listed as urgent threats to human medicine: *Clostridioides difficile*, Carbapenem-resistant *Enterobacteriaceae,* and drug-resistant *Neisseria gonorrhoeae* (CDC, 2013). Another 12 resistant infections were identified as serious threats and included multidrug-resistant *Acinetobacter*, drug-resistant *Campylobacter*, fluconazole-resistant *Candida*, extended-spectrum Beta-lactamase producing *Enterobacteriaceae*, vancomycin-resistant *Enterococcus*, multidrug-resistant *Pseudomonas aeruginosa*, and the previously mentioned methicillin-resistant *Staphylococcus aureus*. The problem is real and has serious consequences for human as well as animal health. The Centers for Disease Control and Prevention estimated that 35,000 deaths occur in the United States each year due to infections with antimicrobial-resistant pathogens (CDC, 2020). The UK government has estimated annual global mortality as a consequence of antimicrobial resistance to be about 700,000 people (Brüssow, 2017).

The American Veterinary Medical Association recently completed a report outlining the pathogens in animal medicine for which antimicrobial resistance has become an issue as well (AVMA, 2020). In swine medicine, the list of pathogens with antimicrobial resistance of concern includes *Escherichia coli*, *Salmonella* spp, *Pasteurella multocida,* and *Streptococcus suis* (AVMA, 2020).

In 1969, the Report of the Joint Committee on the Use of Antibiotics in Animal Husbandry and Veterinary Medicine, the so-called Swann Report, was presented to the British parliament. The report recommended restrictions on the availability and use of antibiotics in animal production and differentiated their use for therapeutic purposes from growth promotion. Since then, much has been written on the apparent relationship between antibiotic use in farm animals and the progression of antimicrobial resistance in human medicine (Baker, 2006; Tang et al., 2017; Kirchhelle, 2018). However, the topic is complex. For example, a report prepared by the UK Department of Health entitled UK Five Year Antimicrobial Resistance Strategy 2013 to 2018 concluded that “increasing scientific evidence suggests that the clinical issues with antimicrobial resistance that we face in human medicine are primarily the result of antibiotic use in people, rather than the use of antibiotics in animals.”

The Centers for Disease Control noted that 30% of antibiotic prescriptions to humans are not necessary; this is equivalent to about 47 million antibiotic courses per year. They have also estimated that 46% of visits to urgent care centers resulted in unnecessary antibiotic prescriptions; this compares to 25% in emergency departments, 17% in medical offices, and 14% in retail health clinics (CDC, 2019). King et al. (2018) reported that at least two-thirds of prescriptions for sinusitis exceeded the antibiotic courses recommended by the Infectious Diseases Society of America. The situation with respect to antibiotic use in human medicine is receiving considerable attention - with some success; between 2011 and 2016, antibiotic prescriptions declined by 5% (CDC, 2019). Even with that improvement, the number of prescriptions written in 2016 would have supplied five out of every six (83%) Americans with one antibiotic prescription annually.

A potential contribution to antimicrobial resistance is antibiotic residues found in meat, milk, and eggs. This is considered to be one of the vehicles by which antimicrobial-resistant bacteria may possibly emerge (Price et al., 2005; Kirbis and Krizman, 2015). The incidence of antibiotic residues in pork appears to be extremely low, although it could vary by geographical region. In the United States, the United States Department of Agriculture is responsible for the National Residue Program for Meat, Poultry, and Egg Products, which actively monitors for the presence of antibiotic residues and other chemical contaminants in meat, poultry, and egg products (FSIS, 2017). It reported that of 7,029 randomly selected samples tested from pigs at U.S. packing plants, 22 were found to contain violative levels of antibiotics, an incidence of 0.3%. Another category of testing involved carcasses deemed by in-plant inspectors to be “at risk,” meaning that for whatever reason, they suspect illness or injury in an individual animal. In this case, 177,238 samples were submitted for assay and 681 were violative, a rate of 0.4%. Testing of imported pork products found a slightly higher violative rate – 0.9%.

The solution adopted by most nations to reduce the risk of selecting for resistant bacteria is to reduce antibiotic use. Outright banning of antibiotics for use in animals is widely recognized as extreme and imprudent as it ignores the welfare of animals affected by a pathogen. In order to reduce the use of antibiotics in meat, milk, and egg production, the current logic, such as that established by the U.S. Food and Drug Administration, promotes more focused use of antibiotics for specific purposes such as 1) treatment of disease in animals that are sick, 2) control of disease in a group of animals when some of the animals are sick, and 3) prevention of disease in animals that are at risk of becoming sick. The use of medically important antibiotics—those employed in human medicine—for strictly growth-promoting purposes or to improve feed efficiency, has been banned in many jurisdictions around the world, such as Sweden (1986), Denmark (1999), the European Union (2006), Bangladesh (2010), Korea (2011), United States (2017), and Canada (2018; Hossan et al., 2018).

Notwithstanding the relative role of human and animal medicine in contributing to the problem, both must be part of the solution; both have much to lose if success is not achieved. The American Association of Swine Veterinarians has a position statement that “Judicious therapeutic use of antimicrobials is a core principle of the broader goal of antimicrobial stewardship. Antimicrobial Stewardship involves maintaining animal health and welfare by implementing a variety of preventive and management strategies to prevent common diseases” (AASV, 2022). In any event, legislation in many jurisdictions has made it clear that reductions in antibiotic use is expected in animal production.

## THE COST OF ABF PORK PRODUCTION

The cost to pork producers of banning antibiotic growth promotants is controversial. There are few properly controlled studies that describe the financial consequences of the ban on overall industry economics or on individual farms. Pluske (2013) identified several impacts of such a ban, including increased mortality, reduction in bodyweight, impairment in feed conversion, increased body weight variation within a group of pigs, and a possible increase in the need for therapeutic medications. There have been a number of national or regional comparisons studying before and after the implementation of antibiotic growth promotant bans. The typical conclusion of such studies is revealed in this statement offered by Tang et al. (2019): “Increasing evidence suggests though, that the benefit of antibiotics for productivity is likely minimal in industrialized production with no significant long-term negative impacts seen when antibiotic growth promoters are eliminated.” While other reports have reached the same or similar conclusions—that the net effect of banning antibiotic growth promotants was less than feared by pork producers (Aarestrup et al., 2010), other studies have been less optimistic. For example, Calleson (2004) reported a decline in average daily gain and increase in mortality in weanling pigs in Denmark; no impact was reported in finishing pigs. Wierup (2001) reported that following the ban in Sweden in 1986, little difference in performance was observed in market hogs, but serious problems occurred in weanling pigs. In the 4 yr after the ban, about 75% of the pigs required antibiotic therapy. However, the use of antibiotics gradually declined, such that over the subsequent 13 yr, total use of antimicrobials declined by 55%.

Fortunately, some controlled comparisons of conventional and ABF production have been reported. It is very difficult to assign a specific cost to the complete removal of antibiotics from pork production. Certainly, there are non-monetary costs, the most prominent of which is reduced welfare of the pigs. Denying pigs antibiotic prophylactic care or treatment during periods of illness or injury runs counter to farmers’ responsibilities to care for their animals. Obviously, it is not possible to assign an economic value to animal suffering, nor should there be.

Main et al. (2010) compared ABF production vs. conventional management. There were 108,000 pigs involved in the ABF system and 611,000 pigs in the conventional system; all pigs originated from the same sow production pyramid and thus were of a similar genetic background and originating health status. The ABF pigs were weaned 5 d later than conventional, received an all-vegetable diet, received numerous vaccines and were given more floor space than that used in normal production. Nonetheless, growth rate was reduced and mortality was considerably higher in the ABF system. The researchers calculated that the added cost of ABF was about $11 per pig sold, and that most of the costs accrued prior to the pigs reaching 23 kg. Indeed, growth performance was similar in both systems during the grow-finish period; this is in agreement with discussion earlier in this manuscript about sub-therapeutic use of antibiotics being in decline during the finishing period due to a lack of response.

Wolter and Gaines (2016) also reported a comparison of ABF vs conventional production conducted under commercial conditions. Average daily gain expressed on a carcass weight basis (−5%; *P* < 0.05), average daily feed (−2%; *P* < 0.05) and gain:feed ratio (−3%; *P* < 0.05) were all impaired under ABF conditions. Most critically, mortality—which greatly impacts the economics of production—more than doubled from 6.5% to 14.1% (*P* < 0.05). The authors reported an increase in the cost of production by 14% to 21%.

Dee et al. (2018) compared three antibiotic treatments in ~2,000 pigs exposed experimentally to the Porcine Reproductive and Respiratory Syndrome virus (**PRRSv**), known to predispose pigs to secondary bacterial infections. One experimental treatment included a standard medication protocol that featured mass medication on days 4 and 21 post-farrowing combined with in-feed antibiotics and individual treatments as needed. The second treatment removed the day 21 mass medication and in-feed medication. Pigs on the third treatment received no antibiotics while on test. Pigs on all treatments received a PRRS vaccine 4 wk prior to exposure to PRSSv. This was a demanding trial, due to the exposure to PRRSv, resulting in mortality and removals of 20.9% and 24.9% on the first two treatments and 58.0% on the ABF treatment. Net income per pig was reduced by 6% on treatment 2 vs. 1, while it was reduced by 68% on the ABF treatment. Rate and efficiency of gain were similar on the first two treatments, but reduced by 26% and 15%, respectively, on the third treatment.

Numerous evaluations of the adoption of ABF production systems reach a similar conclusion - that mortality is the main driver of increased cost and thus needs to be a focus in order to make ABF sustainable in the long term. Labor requirements obviously increase with the more intensive animal care associated with ABF. In one study in Denmark, the added labor was determined to be 1.44 min/wk/sow, which would translate into one additional stock person per 1,600 sows (Bagger et al., 2015). The authors concluded that under the conditions of their study, 50% of pigs could quantify as ABF, reaffirming that the concept of eliminating antibiotic use completely in pork production is an unrealistic target that would place animal welfare at risk (Baker, 2002).

Future experience will no doubt improve performance, welfare, and financial outcomes with ABF production. This should result in a higher proportion of pigs achieving ABF status at the time of marketing.

## REDUCING ANTIBIOTIC USE IN PORK PRODUCTION: AN OVERVIEW

Some animals are currently raised to market on farms operating under an ABF production model, but they represent a small portion of the total, at least in North America. On individual farms, experience suggests that it is possible to have as many as 95% or more of total production enter the ABF market, although a more likely outcome for high health herds is 75 to 85% (Johnson, 2018); the remaining pigs become ill or suffer injury during the course of their lives and require interventions with antibiotics, resulting in their removal from the ABF market stream. However, there are many producers who achieve a much lower level of success—50% or lower—often due to underlying health challenges in their herd or limitations in facilities, genetics, nutrition, or management skills (Fablet et al., 2012). A recent Danish study reported that 64% and 68% of all pigs born on two farms were able to reach 12 wk of age with the use of AB (Lynegaard et al., 2021). In any event, the level of success is likely to improve over time with the benefit of new technologies and greater experience.

It is clear that from the limited experience already gained by the pig industry that success in ABF pork production will demand a multidisciplinary approach involving genetics, health management, building engineering, design, and operation, and animal husbandry as well as adjustments in diet formulation (Table 1). It certainly will not be achieved by simply replacing AB with one or more feed additives. Given the current state of the art, certain minimum operating standards will be required to achieve the greatest level of success; these should be considered minimal requirements and include 1) a pig herd which is free from PRRSv, 2) a high level of building hygiene and disease management combined with heightened attention to biosecurity (Gleeson and Collins, 2015), 3) a well-designed vaccination program 4) minimized social stressors, 5) adoption of a weaning age greater than 24 and possibly as high as 28 d of age, 6) selection of more robust genetics that are more resistant to disease and less affected by common stressors, 7) effective and successful ventilation to ensure a high-quality environment within the barn, 8) feed formulation that considers both nutrient requirements and functional properties of ingredients, 9) an abundant supply of high-quality drinking water, and 10) strong individual perinatal care of the sow and her offspring and of individual pigs at weaning. Upon reviewing this list, it becomes clear that success with ABF production lies in starting with a healthy pig herd, combined with minimizing social, health, and environmental stressors, backstopped by the highest possible level of animal husbandry (Table 2).

**Table 1.** Important topics when considering the adoption of ABF pork production

Diet formulation strategie	Selection of feed additives	Management strategies	Feed processing
• Acid binding capacity	• Antibacterial metals	• Robust genetics	• Particle size
• Carbohydrate sources	• Bacteriophages	• Perinatal & postnatal care	
• Fiber type and level	• Direct fed microbials	• Stressors	
• Functional amino acids	• Enzymes	• Older weaning age	
• Ingredient quality	• Feed fermentation	• Barn environment	
• Protein & amino acid levels	• Lysozyme	• Group size, floor space	
• Specialty proteins	• Medium chain fatty acids	• Body weight variation	
• Functional proteins	• Nucleotides	• Maximize feed intake	
	• Organic acids and their salts	• Water supply	
	• Plant extracts	• Reduce pathogen load	
	• Prebiotics, stimbiotics, etc	• PRRS-free status	
	• Resistant starch	• Building hygiene	
	• Yeast products	• Biosecurity	
		• Strategic vaccination	
		• All-in-all-out prod’n	
		• Herd health management	

**Table 2.** Minimal operating standards for success in ABF pork production

1. Pig herd which is free from PRRSv
2. Strategic adoption of non-antibiotic feed additives proven to be effective
3. High level of building hygiene and disease control combined with biosecurity
4. Well-designed vaccination program
5. Minimized social stressors on the pig
6. High-quality barn environment
7. Feed formulation that considers the functional properties of ingredients
8. High level of perinatal care of the sow and her offspring and of pigs at weaning

Greatest attention must be paid to the individual newborn pig and to the newly weaned pig. These are the times in the pig’s life when the absence or restriction of the use of antibiotics is most likely to generate problems that can impair animal performance but also impact its welfare (Johnson and Lay, 2017). To a lesser extent, the farrowing and lactating sow also requires special attention, in part because she has the potential to impact both the degree of exposure of her piglets to pathogens and their ability to withstand health challenges.

Managing pigs to minimize the impact of disease has its own benefits beyond simply preparing for a future in which fewer antimicrobials will be used. Disease is a costly visitor to a hog farm. For example, Holtkamp et al. (2013) reported that PRRSv alone costs the American pig industry USD 664 million per year. On an individual 1,000 sow farrow-to-finish farm, Nathues et al. (2017) estimated an average reduction in net income of USD 767,000 per year due to PRRSv. Using actual data collected from three co-located commercial wean-to-finish research units with differing degrees of health challenge, Cornelison et al. (2018) determined that for a single 2,400 head wean-to-finish barn, a high health challenge reduced net income per turn by about USD 50,000 compared to a barn with a low health challenge. These few examples illustrate the magnitude of the cost borne by barns with a poor health status.

Particular attention is paid to the microbiome of the young piglet. It can affect overall immunological competence and robustness, but it is also intimately related to physiological functioning of the gastrointestinal tract and nutrition (Fouhse et al., 2016). At the present time, the profile of the ideal, or even acceptable microbiome, has yet to be characterized with any degree of precision, leaving researchers and industry personal with the task of achieving a desired goal that lacks quantitative definition. In concert with a healthy microbial population, gastrointestinal tissue plays an equally critical role in maintaining health and contributing to the survival of the pig, despite the often hostile environment which exists in the lumen of the gut (Moeser et al., 2017). The complexity of the gastrointestinal tissue is truly impressive, as it contributes to digestion and absorption of nutrients, secretes enzymes, and plays a major role in the immune system--all the while maintaining a selective barrier preventing the entry of pathogens or cell fragments that can compromise the health of the pig (Li et al., 2018, 2019).

The process of weaning places stress on the pig as a whole, but in particular on the functioning of the gastrointestinal tract. Recent research suggests that compromised development of the barrier function of the gastrointestinal tract may have lifelong implications for susceptibility to disease due to increased gut permeability, suppression of immune function, and a hyperactive nervous system associated with the gut. These studies have provided compelling evidence that mast cells play a central role in these longer-term pathologies associated with gut function (Moeser et al., 2017; Wilson et al., 2019). In addition to the various stresses imposed on the pig at weaning, other factors may play a role in the magnitude of this issue, including birth weight and gender.

Many of the challenges facing the young pig, some of which persist to market, have clearly been shown to be influenced by feeding regime, the make-up of the microbiome, thermal and other environmental insults, social disruption, poor health management, including biosecurity, and animal care. Weaning age appears to be a leading factor in overall weaning adversity (Moeser et al., 2017; Huting et al., 2019; Faccin et al., 2020c). Another requirement for success in ABF production is the maximization of feed intake in the sow after farrowing and in the piglet immediately after weaning. Lethargy in sows in the first 3 d post farrowing is associated with reduced feed and water intake and thence poor litter growth (Fraser et al., 1993). Feed intake in the newly weaned pig is equally important but also frequently falls well short of expectation (Bruininx et al., 2001). These issues will be discussed in more detail later in this review.

Mortality is increasingly recognized as important in any comparison of production method or nutrition strategy adopted in ABF production. Small differences in mortality can result in large differences in economic returns. The financial impact of modest differences in growth rate or feed efficiency frequently pale in comparison to the effect of small changes in mortality. Although important, measurement of mortality is not a simple task; it requires much larger numbers of animals than is employed in most studies (Gebhardt et al., 2020b). The problem is exacerbated by the fact that mortality is frequently not measured or reported in many published studies.

There are different approaches to the practicalities of integrating ABF into a pork production system. An essential need is a system to identify pigs that maintain ABF status throughout their life, or alternatively, identify those that have lost ABF status due to the need for medical treatment arising from injury or illness. There are numerous approaches to address this need. One option is to tag all pigs in the herd at birth, and remove the tags when pigs receive antibiotic treatment. At the time of harvest, only tagged pigs will enter the ABF flow to market (Lynegaard et al., 2021). A second option is to isolate pigs receiving antibiotic treatment, either by removal to a pen or pens identified for this purpose, or to a new building altogether; the latter is difficult to achieve as it could lead to the mixing of pigs of different ages within the same airspace, something considered anathema to successful health management (Scheidt et al., 1995). The third option is to tag pigs at the time of treatment; unlike the first option, only untagged pigs will be accepted as being ABF at harvest. This approach is not recommended because treated pigs could lose their ear tag and thus be incorrectly identified as AF.

It is abundantly clear that success in ABF production requires a multidisciplinary approach that addresses a wide array of inputs. The objective of this review is to clearly demonstrate, based on the available literature, that success can only be truly achieved when all aspects of diet formulation and animal management are adequately addressed. It is likely that ABF has not achieved as much penetration in the pig market because it is such a complex and expensive process with uncertain success.

## DIETARY APPROACHES TO IMPROVE ABF PRODUCTION

Many of the pathogens that infect pigs in the wean-to-finish phase of production are directed at the gastrointestinal tract; the other major target is the respiratory system. Furthermore, the process of weaning is known to adversely affect mucosal permeability, electrolyte balance, and local cytokine expression (Lallès et al., 2007). It therefore makes sense to turn our attention to diet composition, with the objective of developing feeding programs that assist the pig in resisting gastrointestinal pathologies and disease but to also position the immune system of the pig to more effectively resist respiratory disease as well (Larivière et al., 2021). To this end, the nutritionist might consider such targets as pH adjustment, modification of the microbiome, preservation of gut barrier function, strengthening of the intestinal architecture, and improvement in oxidative status, along with modulation of the immune system, to name just a few (Barba-Vidal et al., 2018; Li et al., 2020; Lallès and Montoya, 2021; Lynegaard et al., 2021). Nutritionists have a large number of tools at their disposal: specific feed additives, selection of functional ingredients for use in diets, and unique formulation strategies which speak to specific health requirements of the pig. This section of the review will address these options.

In an excellent review on the subject of gut function and dysfunction in the young pig, Lallès et al. (2004) identified key contributors to an impaired gastrointestinal tract. For example, the newly weaned pig is struggling with a general immaturity combined with psychological and dietary stresses, the latter of which is the result of disrupted access to milk, forced dependence on feed which is now provided in dry form, and the need to access water independent of the feed. This leads to intestinal disorders such as alterations in the architecture of the gut and the presence of enteric pathogens. Their description of disturbance of the gut is cause for concern; it includes alteration in its morphology, including villus atrophy, reduced levels of activity of digestive enzymes and disturbance in intestinal absorption, secretion and permeability, mucosal disruption associated with mast cell activation, and constant exposure to pathogens which prior to weaning was blunted somewhat by a modicum of protection afforded by their mother’s milk. The main risk factors at play in the newly weaned pig, include low and erratic feed intake, completely unfamiliar antinutritional factors in the feed, and a diet which is probably not as digestible as sow’s milk but contains a high level of protein and possibly significant undesirable buffering capacity. On top of all of these dietary stresses, the newly weaned pig may be over-crowded, experience mixing with unfamiliar litters, living with poor hygiene, and a physical environment that may not be optimal for its needs (cool, drafty, etc). The pig certainly experiences a diversity of challenges at the time of weaning; the magnitude of the impact is dependent, at least in part, on weaning age (Moeser et al., 2007; Faccin et al., 2020c).

Given the array of insults experienced by the pig at the time of weaning and associated with diet change, it makes sense to evaluate feed additives to assist the pig during this transition period. The conditions under which feed additives are evaluated has been shown to be of critical importance. As one example, Olsen et al. (2018) compared feeding regimes with or without antibiotics in the feed, and also investigated two additional feed additive treatments. This study was conducted in a commercial scale research barn with 1,300 mixed sex pigs with an average initial body weight of 6.1 kg and weaned at an average of 21 d of age. The pigs were housed in large vs. small group sizes consisting of 31 or 11 pigs each; importantly, floor space per pig was kept constant, and feeder access was similar, although not quite identical. Pigs in the smaller group size grew faster and were more feed efficient in the absence of antibiotics compared with pigs housed in the larger groups. The proportion of pigs requiring medical treatment was greater in the larger group size pens as well. In the presence of antibiotics in the feed, there was no difference in performance between the two group sizes. Neither of the non-antibiotic feed additive regimes affected growth performance, irrespective of group size. It was clear from this study that if only one group size had been investigated, the authors may have drawn very different conclusions. The authors proposed that in all studies involving the evaluation of non-medical feed additives, the health status, physical environment, and genetics of the pigs should be very well characterized and recorded for the benefit of the reader; this would enable more effective comparison of results across multiple studies (Bedford and Masey-O’Neill, 2016).

### Diet Formulation Strategies

Diet formulation for ABF production will require careful selection of proper levels of energy and nutrients, concurrent with choosing the right ingredients to meet these requirements. Concurrently, selection of appropriate ingredients is much more important in the ABF newly weaned pig because the functional properties of the diet, as discussed below, will play a critical role in achieving success. Finally, the feed must be processed properly and presented to pigs in a manner that encourages maximum intake.

Why is this so important in the newly weaned pigs, and why is the ABF pig more susceptible to a growth slump accruing from health problems at weaning? This is obviously a complex issue, and the solutions are many and varied; indeed, diet formulation is only one part of the nutritional program that supports the growth, health, and welfare of the newly weaned pig. In its simplest form, the young pig experiences a general decline in the integrity of the intestinal tract, a consequence of low feed intake, insults accruing due to changes in nutritional, environmental, behavioral, and physiological stressors, and gut inflammation (Heo et al., 2013). The problem is exacerbated by the removal of sow’s milk from the pig’s diet, resulting in the loss of important functional components such as fatty acids, oligosaccharides, biogenic amines, and bioactive peptides, which are not easily supplied in a typical post-weaning dry diet (Hurley, 2015; Metzler-Zebeli, 2022). A related and equally disruptive outcome is gut dysbiosis.

Ingredient choice in diet formulation is consequently so important because feedstuffs do much more than simply provide energy and nutrients to the pig; increasingly, nutritionists are recognizing the functional properties of ingredients as well as their nutrient content (Patience, 2017). The selection of certain ingredients, or more appropriately, their chemical profile and associated functional properties, can either help the pig resist disease or they can predispose the pig to certain illnesses, especially those of the gastrointestinal tract (Helm et al., 2021). They can also impact such things as rate of passage, the profile of the microbiome, bulkiness of the digesta and feces, viscosity of the digesta, anti-oxidative properties, impact on the immune system, enhanced or impaired sense of satiety, and physical abrasiveness. The list is rather long and becomes much more important in ABF production.

The functional properties of ingredients may be associated with certain proteins, fiber, fatty acids, and even minerals. A familiar example of a protein with functional attributes is the immunoglobulins which may be found in ingredients derived from milk or blood (Owen et al., 1961; Kats et al., 1994; Kar et al., 2016). As a mineral, limestone has a functional property, that being its ability to serve as a buffer, especially in the stomach; ZnO can have the same effect (Hajati, 2018). Ingredients can be processed to reduce or remove negative functional activities; a common example would be fermentation of soybean meal. Zheng et al. (2017) reported that Bacillus fermentation of conventional soybean meal reduced glycinin from 149 to 21 mg/g, β-conglycinin from 104 to 31 mg/g and trypsin inhibitor from 38 to 2 mg/g. However, perhaps the most recognized though perhaps least understood example of functional properties of ingredients relates to taste and other sensory perceptions (Roura and Fu, 2017).

When feeding the newly weaned pig, which has relatively immature and sensitive digestive, immune and microbiological systems, improper selection of ingredients can lead to serious health and productivity problems. On the other hand, selecting the correct ingredients with the needed functional properties can greatly enhance the piglets’ transition from nursing the sow to consuming dry feed. Older pigs, with a more mature physiology, are much less sensitive to these problems. Even so, it is well known that ingredient selection in diets for growing and finishing pigs can leave them susceptible to serious health problems; as one example, Wilberts et al. (2014) reported that corn DDGS could possibly increase a growing pig’s susceptibility to swine dysentery, while replacement of this insoluble fiber source to one which is more readily fermented appears to provide protection (Helm et al., 2021). In the same vein, the benefits of including milk products such as whey or casein, and certain plasma products, in the diet of the newly weaned pig are well established (Grinstead et al., 2000).

#### Acid-binding capacity.

A low pH in the stomach is an essential part of effective protein digestion; it also serves as an important line of defense against the inadvertent entry of pathogens into the gastrointestinal tract via the feed (Zhu et al., 2006). Unfortunately, regulation by the newly weaned pig of gastric pH is poorly developed. The problem becomes more acute in ABF production because antibiotics are not available to protect against food-borne pathogens. The selection of ingredients for inclusion in the diet can also contribute to this problem.

Diet composition appears to affect gastric pH and thus protection against feed-borne bacteria. For example, Lagos et al. (2021) fed diets containing lactose as well as spray-dried plasma, and enzyme-treated soybean meal in addition to traditional corn and soybean meal. This achieved a pH of 2.7, whereas Radcliffe et al. (1998) fed diets based solely on corn and soybean meal and observed pH in the stomach of 3.6 and 3.8 in two experiments. Zhu et al. (2006) concluded that a pH of 2.5 was required to achieve significant bacteriocidal effects, with pH of 3.5 much less effective, unless proteolytic enzymes were present. It therefore would be logical to assume that buffers in the diet would have greater impact when the basal pH of the stomach is below 3.5 and ideally below 2.5.

Feed ingredients vary in their acid-binding and buffering capacity. Acid binding capacity is determined by titrating a feed or an ingredient with 0.1 N HCl to achieve a final pH of 3.0 or 4.0 or even 5.0; the answer is expressed in mEq/kg (Jasaitis et al., 1987). A final pH of 3.0 would be considered most relevant to the circumstances of the young newly weaned pig (Lawlor et al., 2005). Buffering capacity is determined by dividing the acid binding capacity by the change in pH from the start to the end of the titration, and thus represents the quantity of acid required to achieve a one-unit change in pH. Furthermore, elevated gastric pH will impact proteolytic activity, which in turn can lead to impaired feed digestion, leading the greater fermentation in the gut as opposed to the more desired digestion (Lawlor et al., 2005).

Ingredients can be loosely categorized as having low (0 to 500 mEq/kg), medium (500 to 1,500 mEq/kg), high (1,500 to 5,000 mEq/kg) or very high (>5,000 mEq/kg) acid-binding capacity. Most grains would be considered to have a low acid-binding capacity while most vegetable protein sources would have medium acid-binding capacity; for example, soybean meal has an acid-binding capacity of about 1,000 mEq/kg (Hajati, 2018). Limestone has an extremely high acid-binding capacity, in the range of 20,000 mEq/kg, while dicalcium phosphate sits at about 8,000 mEq/kg (Hajati, 2018). ZnO has greater acid-binding capacity than limestone, but is added to the diet at much lower levels. Lawlor et al. (2005) provides a comprehensive listing of ingredients along with their associated acid-binding capacities.

While the topic of acidification of diets is not new, the concept of formulating diets on the basis of buffering capacity or acid-binding capacity has only recently started to translate into commercial practice, largely based on theoretical expectations, as explained above, and unpublished data. Research is clearly needed on this topic. Given that the newly weaned pig is not well positioned to excrete sufficient acid by itself, studies on the reduction of limestone in the phase 1 (days 0–7) starter diet should probably include some form of acidifier (Radcliffe et al., 1998); the benefit of a diet with lower buffering capacity may be to maximize the benefit of acidifiers rather than simply to assist the pig in lowering gastric pH. However, the most recent publication on this topic failed to support this hypothesis (Lagos et al., 2021).

Of course, reducing limestone to reduce diet buffering capacity is problematic, because the pig’s calcium requirement must be met, and limestone is usually the lowest cost option available. Data are required to determine if feeding diets deficient in calcium for a short period of time (<1 wk) will adversely affect long-term skeletal development. This appears to be a topic needing further detailed investigation, especially as it relates to ABF production. An alternative strategy to lower acid-binding capacity would be to replace calcium carbonate with the calcium salt of an organic acid such as calcium formate (Lawlor et al., 2006).

#### Carbohydrate sources.

The quantity and type of proteins, fats, vitamins, and minerals in diets for the newly weaned pig tend to receive much more attention than the quantity and type of carbohydrate. When the conversation turns to carbohydrate, the focus is typically on fiber sources and content, a very important topic, of course, which will be discussed later in this review. Failure to consider the simple carbohydrate component of the pig’s diet ignores an important source of energy and the functional properties that it provides in the diet of the newly weaned pig. For example, at the time of weaning at 21 d of age, sow’s milk typically contains 26% lactose on a dry matter basis (Hurley, 2015); therefore, the nursing piglet receives approximately 20% of its net energy calories from lactose. It is not a coincidence that this is approximately the portion of energy derived by the pig from lactose in a typical phase 1 starter diet (days 0–7); nutritionists have tended to select the quantity of lactose in a phase 1 diet to reflect that present in sow’s milk.

However, does the pig require lactose in its diet, or is lactose even the best source of carbohydrate energy at the time of weaning? The fact that it exists in abundance in sow’s milk leads most nutritionists to assume that it is. Yet, lactose has other benefits than simply supplying energy to the pig, such as serving as a source of valuable peptides and oligosaccharides (Dallas et al., 2014). Also, while most lactose will be digested enzymatically to glucose and galactose, a portion will be fermented to short-chained fatty acids and in the process, lower gastrointestinal tract pH to levels which should impair the proliferation of pathogenic bacteria (Jang et al., 2021). As the pig grows older, the concentration of lactase produced in the intestinal epithelium declines (Pluske et al., 2003). For this reason, and to lower feed cost, the quantity of lactose in the diet is reduced as the pig proceeds beyond weaning. Typically, 15 to 20% lactose is included in the phase 1 diet of pigs weaned at approximately 3 wk of age and fed for 7 d, followed by a phase 2 diet containing 10% to 15% lactose for the subsequent 7 to perhaps 14 d. There appeared to be almost no limit to the pig’s response to increasing levels of lactose during the first week after weaning. Interestingly, the presence of antibiotic growth promotants in the diet muted the lactose response (Zhao et al., 2021). Beyond phase 2, lactose is rarely recommended, unless the feed is being directed to pigs with compromised health or performance, or are lighter in weight than normal.

It should also be kept in mind that the use of various milk by-products can be problematic due to their negative impact on pellet mill throughput. Appropriate combinations of diet moisture level, heat, and shear help to minimize their impact in this regard (Dunmire et al., 2020). Increasing the level of fat in the diet by adding fat directly or utilizing basal ingredients with higher endogenous fat content is frequently recommended.

The use and levels of lactose in starter diets is being questioned for reasons other than processing efficiency. Lactose is an expensive ingredient and the cost of its use is likely to only increase in the future. Furthermore, researchers have investigated alternative carbohydrates with interesting results. Dextrose, sucrose, liquid lactose and molasses have all been proposed as full or partial replacements for dried lactose (Dunmire et al., 2020; Zhao et al., 2021). Recently, Clouard et al. (2018) reported that using maltodextrin in place of lactose in a liquid milk replacer improved cognitive performance 8 wk later and did not impact growth performance.

#### Fiber type and level.

 The potential role of dietary fiber to mitigate stress and disease is of growing interest to nutritionists and pork producers, particularly those interested in ABF production, but it is also a source of considerable controversy and uncertainty. Fiber represents that portion of plant cells which are resistant to hydrolysis by enzymes secreted by the alimentary system of the pig. It therefore includes such entities as cellulose, hemicellulose, lignin, gums, waxes, oligosaccharides, β-glucans, and pectins (Trowell et al., 1976; Patience and Petry, 2019).

One of the great challenges with fiber is finding assays which define its chemical composition in terms that help us to better understand its physiological and nutritional function. Tremendous progress has been made in this regard in the past 60 yr. The oldest assay, referred to as crude fiber, was developed more than 200 yr ago; it has since been found to possess many limitations, including incomplete recovery of cellulose (50 to 80%), hemicellulose (20%), and lignins (10 to 50%; Van Soest and McQueen, 1973). Yet, crude fiber is still frequently measured, although it has little if any value in swine nutrition. Starting in the 1960s, Van Soest developed and subsequently refined the detergent system for defining the fiber content of animal feedstuffs. Neutral detergent fiber (**NDF**) and acid detergent fiber have now been used, even in monogastric species, for more than half a century, and remain a foundation for fiber analyses in the field. In general, neutral detergent fiber captures hemicellulose, cellulose, and lignin; acid detergent fiber quantifies cellulose and lignin (van Soest et al., 1991). The detergent system works reasonably well for fiber which is highly insoluble, such as that found in corn and corn co-products, but has less value if the fiber contains a significant soluble fraction, which is excluded from the assay.

Because of this limitation, the assay for total dietary fiber (**TD**) is growing in popularity in swine nutrition. Cost and accessibility remain barriers to more routine application on the farm, so it is largely restricted to use in research; however, it is being used in commercial practice with increasing frequency. The advantage of the total dietary fiber (**TDF**) assay is its ability to quantify both soluble and insoluble fiber fractions (AOAC International, 1995). Insoluble fiber includes celluloses, hemicelluloses, and lignin; although lignin qualifies as fiber due to its being refractive to enzyme digestion, it is essentially inert and generally is not considered terribly relevant in discussions on fiber. Soluble fiber includes, for example, pectins, gums, β-glucans, and resistant starch.

The final option for fiber analysis is to determine total non-starch polysaccharides (**NSP**); like TDF, its cost, complexity, and the time it takes to complete, makes it less popular in commercial practice than simpler but less robust options. Whether or not total NSP is similar to TDF will depend on the specific assay procedure being used; some methods will exclude lignin and other polyphenols and therefore will be quantitatively less than TDF, while others include these compounds, in which case NSP and TDF will be very similar.

The impact of a given source of dietary fiber on the digestive tract of the pig can be largely attributed to one or more of the following characteristics: the quantity of fiber in the diet and its solubility, fermentability, water binding capacity, and viscosity (Bach Knudsen, 2001). Generally, it is assumed that highly soluble fiber will also be fermentable and contribute to viscosity of the digesta in the small intestine. Conversely, insoluble fiber is generally considered poorly fermentable and not viscous. These generalities are not necessarily true; for example, soy hulls are insoluble but highly fermentable (Jaworski and Stein, 2017). Sugar beet pulp contains 74% total dietary fiber, of which 29% is soluble. Uronic acid and pectin contribute to this soluble component, giving it a very high water-holding capacity—three to five times higher than wheat or barley (Li, 2018)! Thus, while solubility, fermentability, viscosity, and water-binding frequently align, that is by no means always the case.

A final issue with fiber assays lies in the fact that not all insoluble fiber acts alike in the gastrointestinal tract; this is even more true with soluble fiber, whose actions in the gut can be highly diverse. Thus, simply assays for “soluble” or “insoluble” fiber fails to predict the action of the fiber in the gut of the pig.

Some data would suggest that poorly fermented, insoluble fiber is most beneficial for the young pig going through the transition of weaning, because it enhances rate of passage and provides a degree of abrasion along the length of the intestine, thus impeding adhesion of pathogens to the gut epithelium (Heo et al., 2009). An opposing view, which is clearly in the minority at the present time, is to provide a more fermentable fiber, to encourage proliferation of commensal bacteria, and thus aid in the maintenance of a healthy gut (Jha and Berrocoso, 2015; Li et al., 2019). Considering the literature in totality, it is very likely that in the future, pig diets, especially those fed during periods of stress such as weaning, will be formulated to contain a specific balance between soluble and insoluble fiber. This ratio will no doubt vary according to the relevant pathogen challenge and other circumstances in the barn (Jha and Berrocoso, 2016; Li et al., 2020). As one example, Hermes et al. (2009) suggested that 4% wheat bran and 2% sugar beet pulp, both sources of soluble fiber, would be a good starting point for practical diets; this will, no doubt, depend on the overall composition of the basal diet, which in this instance consisted of rice, barley, sweet whey, potato protein and 44% crude protein soybean meal.

Looking at the broad array of literature on this topic, the optimum quantity of fermentable fiber in the diet may be influenced by the amount of protein reaching the lower gut. If the diet is low in protein, or contains highly digestible protein, such that the quantity of protein reaching the lower gut is low, encouragement of fermentation may be beneficial, as it could favor such species as lactobacillus, bifidobacterial, etc. However, if there is a likelihood of large quantities of undigested protein reaching the lower gut, encouraging fermentation may simply exacerbate the formation of toxic amines and other products of protein fermentation. The issue of undigested protein reaching the lower gut may help to explain the divergence of opinion among nutritionists regarding the benefit or lack of benefit from the use of fermentable fiber in phase 1 (days 0–7) and 2 starter diets (days 7–21).

#### Functional amino acids.

 Individual amino acids, frequently but not always those that are normally considered non-essential or dispensable in the diet, may play a role in pig health and performance, especially around the time of weaning. Consequently, if non-essential, they may need to be supplemented in the diet of newly weaned pigs when normally they are not, or supplemented at levels higher than that required to maximize protein accretion if they are considered essential in the diet (Rezaei et al., 2013; Rodrigues et al., 2021). These “functional” amino acids, so named because they fulfill a specific function beyond growth, may become more important in the diet of pigs not receiving the benefit of antibiotics in their feed or water.

Glutamine and glutamate are both effective sources of energy for enterocytes and have been proposed as a means of minimizing intestinal atrophy, a common observation at the time of weaning (Domeneghini et al., 2006). Alanine and glycine appear to stimulate the release of the so-called anti-secretory factor, which provides protection against diarrhea (Lange and Lonnroth, 2001). However, in these instances, experimental evidence of actually improving pig performance is inconsistent. Perhaps the most encouraging recent data have been reported by Duttlinger et al. (2019; 2020); when weaning and transportation stress were combined, adding 0.2% to 0.4% L-glutamine improved rate and efficiency of gain, although the benefit was lost by the end of the growout period. Data demonstrating a consistent and sustained benefit, including financial value, from the supplementation of non-essential amino acids in diets for stressed pigs is required before this practice becomes more common in commercial practice in the pig industry.

Individual amino acids, and blends of individual amino acids, have also showed encouraging results in the diets of newly weaned pigs (Le Floc’h et al., 2018). Wessels et al., 2021 reported that the addition of multiple synthetic amino acids provided benefit in terms of fecal dry matter and expression of MUC-2, an important protein involved in defense mechanisms, but did not impact growth performance. Rodrigues et al. (2021) reported that supplementation of nursery diets with threonine, methionine, and tryptophan improved growth performance and immune status of *salmonella*-challenged 14 kg pigs. Numerous other examples exist in the literature.

#### Ingredient quality.

 Successful diets for swine require a consistent and high level of feedstuff quality, especially when feeding the young pig. This becomes even more critical when feeding pigs without the benefit of antibiotics in the feed; stressors on the gastrointestinal tract are particularly troublesome when low quality, damaged, or contaminated ingredients are used. In the instance of ingredients derived directly or indirectly from crops, quality issues can arise during the growing season, harvest, storage, and processing. In the instance of manufactured ingredients, quality will be impacted starting with the selection of the originating feedstock and continuing through processing and delivery to the feed mill and handling of the final mix feed. The importance of an effective quality assurance program to ensure consistency of feed quality is readily apparent (Patience, 1996).

Ingredient quality may refer to such concerns as freedom from heat damage, insect infestation, chemical and biological toxin contamination, or deterioration during storage (Suleiman et al., 2013). It can also refer to the inherent variation in energy and nutrient content which exists in all grains and protein sources (Fairbairn et al., 1999; Zijlstra et al., 1999). Because of this variation in quality within and among ingredients, purchase price should not be the highest priority when selecting ingredients for purchase, especially when they will be used in the production of diets for the young pig. Cost is certainly important, but not at the expense of the health and performance of the pig.

Heat damage of ingredients can occur during harvest, such as when grains or certain protein sources are dried at an excessive temperature. The most serious concern regarding the application of excess heat relates to amino acid availability, but energy can also be impacted (Van Barneveld et al., 1994; Columbus and de Lange, 2012).

Milk products, such as those used in phase 1 (days 0–7) and 2 starter diets (days 7–21), may vary in quality due to the degree of control over the drying process, titratable acid level, and pH (Nessmith et al. 1997). From a diet formulation perspective, it is also helpful to measure total and NPN protein, fat, and ash content. There are different whey products available in the feed ingredient marketplace and they all derive from either acid whey or sweet whey. Most that are available to the pig industry as a feed ingredient will be based on sweet whey; acid whey tends to be more challenging to dry. Acid whey is the by-product of yoghurt and cottage cheese production; as its name suggests, it is the result of fermentation of milk using lactobaccilli or by acid coagulation using citric, acetic, lactic, hydrochloric, or sulfuric acids. It tends to have a lower protein content than sweet whey. Sweet whey is generated from the coagulation of casein in milk using rennet or a combination of chymosin and pepsin; this leads to the manufacture of hard cheeses and tends to have a higher protein content than acid whey (Rocha-Mendoza et al., 2021).

Whey permeate is an ingredient commonly used in starter diets for pigs; it contains about 80%–85% lactose, 9% ash, and 3% crude protein. Therefore, whey permeate is included in the pig’s diet as an energy/carbohydrate source and not as a protein source. If a source of milk protein is desired, then a preferred product would be dried whey, which contains about 12% crude protein and 72% lactose (NRC, 2012). It is not commonly used in pig diets due to cost, although this can vary according to location. Another milk product option is casein, but it too is quite expensive and not commonly used.

Soybean meal is another example of a protein that can vary in quality if not processed correctly. For example, inadequate heating will result in excessive levels of anti-nutritional factors such as urease and trypsin inhibitor, while over-heating will inactivate or destroy amino acids such as lysine and methionine. These are a few examples to illustrate that quality control must consider how the ingredient is processed and whether such processing has been adequately managed.

Chemical and biological toxin contamination can be a serious practical problem, resulting in significant loss of energy and nutrient value; mycotoxins are one frequent concern in many parts of the world (Bryden, 2012). Storage is frequently overlooked in discussions on feedstuff deterioration, but months-long retention of ingredients or mixed feeds can be problematic, as demonstrated by Dierick and Decurpere (2002) in the instance of fatty acids. Insect pests can also be a serious problem if not identified and addressed early in the infestation (Larson et al., 2008).

The quality of fat utilized in all diets is important, but it is particularly critical in starter diets due to the immature status of the pig’s intestinal tract. Shorter chain lengths and unsaturation of fatty acids delivered as triglycerides are preferred by the young pig but are less important as pigs age (Weng, 2016); the utilization of longer chain fatty acids requires success in a sequence of events which include emulsification involving bile, hydrolysis by lipases present in saliva, gastric and pancreatic secretions, and the formation of mixed micelles and uptake by enterocytes, ultimately leading to delivery to the lymphatic system (Kerr et al., (2015). Van Heugten et al. (2016) reported that as the degree of peroxidation of the lipid increased, there was a corresponding rise in mortality, the number of culled pigs at the time of harvest and the number of pigs requiring medical treatment. Kellner et al. (2017) evaluated 14 commercial fat sources of plant and animal origin, reporting a 30% range in net energy content for the 13 kg pig: from 5.95 to 7.76 Mcal/kg; the free fatty acid content ranged from nil to more than 130 g/kg while MIU (moisture, insoluble impurities, unsaponifiable matter) content varied from 2 to 11.6 g/kg. The energy content of the fat source was most affected by its free fatty acid content, MIU content, and the omega-6:omega-3 ratio. However, much of the literature suggests that the newly weaned pig does not typically respond to dietary fat addition with improved growth rate although feed efficiency may be improved (Tokach et al., 1995; DeRouchey et al., 2004).

Because of the wide variation which exists in many feed ingredients in use today, pork producers and nutritionists interested in ABF production have a particular interest in acquiring higher quality products as a means of achieving maximal performance but also minimizing stress on the pig’s gastrointestinal tract.

#### Protein and amino acid levels.

There is a diversity of opinions throughout the world on the most appropriate strategies for supplying essential amino acids to the newly weaned pig. The concern receiving the greatest attention focuses on the quantity of fermentable protein which reaches the lower gut, resulting in post-weaning diarrhea. There are also concerns that, motivated by reduced costs, lowering the levels of specialty protein sources, such as processed plant proteins (e.g., enzyme-treated soybean meal, soy protein concentration, pea protein concentrates, etc) and animal proteins (e.g., fish meal, proteins derived from blood, etc), may have negative consequences for gut health in the newly weaned pig, at least in some circumstances. There are also questions about the fundamental levels of essential and non-essential amino acids required in the diet in ABF production systems. The first issue is the most prominent in the literature and stems from the concern that undigested or unabsorbed protein will reach the large intestine where it will ferment and produce chemicals which at best are irritating and at worst toxic: branched-chain volatile fatty acids, biogenic amines, indoles, volatile phenols and, of course, ammonia (Halas et al., 2007). Their production in the lower gut is frequently associated with changes in the composition of the lower gut microbiome, which also can lead to post-weaning diarrhea. The young pig has limited ability to digest plant protein in the early post-weaning period, in part due to limited capacity for gastric acid secretion to initiate protein digestion. It is therefore not surprising that the nature and severity of post-weaning diarrhea can be impacted by the level and digestibility of protein in the diet and the secondary and tertiary structure of such proteins. Pieper et al. (2016) suggested potential involvement of anti-nutritional compounds and the secretion of endogenous proteins. The severity and impact of post-weaning diarrhea appears to be related to the balance between fermentable protein and fermentable carbohydrate in the gastrointestinal tract (Bikker et al., 2006; Jeaurond et al., 2008; Kim et al., 2008). Kil and Stein (2010) reported that a Danish study conducted under commercial conditions lowered the incidence of post-weaning diarrhea by 25% by lowering dietary crude protein content from 21% to 18% while maintaining levels of essential amino acids above the pig’s requirement. Heo et al. (2009) lowered crude protein from 24% to 18% while maintaining essential amino acid requirements for 7 d post-weaning; animal performance held constant while reducing post-weaning diarrhea.

However, feeding lower protein diets has not always been successful in controlling post-weaning diarrhea or maintaining pig performance (Batson et al., 2021). Two complicating factors inherent in this approach are the possibility of negatively impacting piglet growth performance, reported in some studies and noted above, and higher diet cost, especially when amino acids other than lysine, methionine, and threonine are supplemented in crystalline form.

Some authors have suggested that crude protein levels below 18% may be needed to adequately control post-weaning diarrhea when antibiotic growth promotants are not available. Under such conditions, diet cost can become prohibitive, since the more expensive synthetic amino acids tryptophan, isoleucine, and valine may be required. It should be noted that this very low protein diet would normally be fed for a limited length of time, frequently when feed intake is quite low and therefore the cost of the more expensive diet expressed on a per pig basis represents a tiny fraction of the total feed cost to market. Furthermore, as crude protein levels decline, care is required to maintain sufficient dispensable amino acids to meet the pig’s need for non-essential amino acid nitrogen (Wu et al., 2011). One approach is to maintain a maximum ratio of SID lysine:total crude protein. Millet et al. (2018a, b) summarized 8 studies, 2 of their own and 6 others from the literature and reported that the mean maximum acceptable ratio appeared to be 0.067 for pigs between 7 and 48 kg.

Another option is being considered, and that is to lower the levels of essential amino acids in the diet to 70 to 75% of the pig’s requirement. This allows dietary crude protein to be substantially reduced at much lower cost; impairment of growth performance while the deficient diet is fed is expected and accepted, in an attempt to control post-weaning diarrhea in ABF systems. Preliminary, unpublished data suggest that the early loss in growth performance is compensated for by the end of the nursery period, typically 42 d.

A final approach to reducing the quantity of fermentable protein reaching the lower gut is to feed highly digestible protein sources. In this way, proteins are catabolized in the upper gut and amino acids are absorbed prior to the digesta reaching the lower gut. This is the approach that has been adopted by the industry for decades but with inconsistent results. The fact that only partial or uneven success is achieved is probably due to the immature and variable status of the digestive system of the newly weaned pig, rather than the quality of the diet. No matter how digestible the protein may be, excess undigested material reaches the lower gut due to the failure of the poorly developed gut to function effectively. Under ABF production conditions, high-quality proteins will remain an important part of the newly weaned pig’s diet, but the quantity in the diet may be reduced in order to achieve health and performance objectives.

The dietary protein story has experienced an interesting twist with the release of the data of Moran et al. (2017), which draws a completely opposite conclusion from that presented above. They showed that in PRRSv positive pigs, phase 1 diets (days 0–14) containing either 15% or 25% soybean meal supported equivalent growth performance. Mortality was unaffected. Smith and Dilger (2018) recently reviewed the potential of soybean-derived isoflavones and saponins for use in pig diets. Isoflavones are known to have anti-inflammatory and anti-oxidative properties, and a possibility for anti-viral effects as well. They have been shown to improve immunological status in disease-challenged pigs, including PRRS. Earlier, Greiner et al. (2001) reported that the soybean isoflavone genistein decreased serum PSSRv concentration and enhanced pig growth performance when fed to pigs injected with the virus.

Finally, there is an outstanding question about the amino acid requirements of pigs raised under ABF conditions. One of the advantages of including antibiotic growth promotants in pig diets is improved nutrient digestibility and reduced demands for nutrients by the immune system, as previously discussed (Visek, 1978). It therefore makes sense that in their absence, requirements may be increased for nutrients such as amino acids. Limited publications exist on this topic, but there is sufficient data to support the need for a better understanding on this subject. Bikker and Dirkzwager (2003) reported that amino acid requirements were increased by about 5% when antibiotic growth promotants were excluded from the diet. Ren et al. (2021) recently reported that the sulfur amino acid requirements are elevated in diets containing no antibiotics. Rakhshandeh et al. (2014) reported that pigs experiencing immune system stimulation, something more likely to occur under ABF conditions, had a greater sulfur amino acid requirement for maintenance. These data support the need for additional studies on amino acid requirements under ABF and non-ABF conditions.

#### Specialty and functional proteins.

Specialty proteins refer to those included in specific pig diets due to some valuable nutritional characteristic, such as being highly digestible, possessing a highly desirable balance of amino acids, or contributing to increased feed intake immediately after weaning. They may also be selected for use in certain diets because they lack, or are low in, antinutritional factors. They are generally too expensive to be employed routinely in the pig’s diet, but because of their specific nutrient or chemical characteristics, may be used in a starter diet to reduce the concentration of less expensive proteins in the diet that, for example, contain antinutritional factors and therefore may impair growth.

Examples of specialty proteins used in pig starter diets include fish meal, milk casein, whey protein concentrate, pea protein concentrate, fermented soybean meal, enzyme-treated soybean meal, and soy protein concentrate. Since these protein sources are by-products and frequently involve heat processing, quality control is an important consideration because excessive heating can impair amino acid digestibility and overall ingredient palatability.

In contrast to specialty proteins, functional proteins are those which have roles in the diet other than, or in addition to, simple nutrient supply. Examples of functional proteins include spray-dried blood meal, spray-dried blood cells, and spray-dried plasma proteins (porcine or bovine). The distinction between functional and specialty proteins can sometimes blur, especially when some protein ingredients can meet both criteria; for example, spray-dried plasma protein is highly digestible, and therefore is considered a specialty protein, but also fulfills unique roles related to immune function, health, and feed intake, which make it a functional protein. The distinction is important, however, because specialty proteins will be formulated into the diet based solely on their energy and nutrient profile, while the inclusion of functional proteins is much more nuanced because the science is still evolving. Functional proteins will, of course, provide nutrients and energy, but they will also be employed to enhance gut health and architecture and possibly the microbiome. In this respect, functional proteins differ from feed additives, which are employed to provide benefits to gut health or the microbiome, but they rarely contribute significantly to the quantity of nutrients provided by the diet.

Spray-dried porcine plasma may be one of the best known and most studied functional proteins, which also can serve as a specialty protein. The isolation and use of spray-dried plasma protein as a supplement for young pigs dates back to the 1970s (Delaney, 1975). They became a staple in phase 1 (days 0–7) and sometimes phase 2 nursery diets (days 7–21) to encourage feed intake leading to a boost in growth rate, especially when pigs are weaned at 3 wk of age or earlier; the greatest boost in animal performance occurs in the first week post weaning. Typically recommended inclusions of 4% to 8% are reported (Perez-Bosque et al., 2016). If they are used in phase 2 diets, and this is rare due to cost, the inclusion rate is typically reduced by 50% to 75% of the level in phase 1.

Alternative but related products, such as spray-dried bovine plasma has been developed and frequently adopted due to cost or to avoid the feeding of animal products back to the same species. However, the benefit of spray-dried bovine plasma appears to be somewhat less than that achieved by using spray-dried porcine plasma (Torrallardona, 2010). The mode of action of such products has not been completely verified, but it appears at least in part to be through modulation of intestinal immune function and inflammation and enhancement of gut barrier integrity (Peace et al., 2011).

A specialty protein that appears to be gaining interest is egg yolk antibodies or dried egg protein derived from hens vaccinated against specific pathogens (Wiedemann et al. 1991). This approach was developed more than 30 yr ago, but is only achieving general acceptance as a viable commercial technology now, largely due to the reduction in cost and the efficiency of processing the product. Experimental results showing improved growth rate and reduced incidence of diarrhea are encouraging.

In reporting the results of a study conducted on a commercial research farm where pigs were naturally exposed to a number of disease challenges, Ruckman et al. (2020) noted that both spray-dried plasma protein and dried egg protein improved the performance of pigs receiving no antibiotics in the feed to levels similar to that of pigs receiving antibiotics. There were no benefits when antibiotics were included in the diet, however. These data remind us that when evaluating diet formulation options for ABF production, experiments conducted with the use of antibiotics may not be helpful.

### Feed Additives

The list of feed additives is truly exhaustive and will no doubt grow further in both breadth—new categories of products—and in depth—more products within existing categories. Many of these products are most effective under a specific set of conditions. This speaks to the need for clarity on the mode of action of each product or category of product, in order to maximize its utility on the farm. Given the wide array of housing conditions, feeding programs, health statuses, environmental conditions, and approaches to animal husbandry under which pigs may be studied in research or raised on farms, it is not logical to expect a single product to perform equally and effectively in all instances. Because of the cost and complexity of research on modes of action, there is limited information on many products, so focusing their use to specific circumstances is challenging at the present time. As a consequence, it is likely that pork producers will incorporate multiple products into their diets to maximize the benefit to their pigs. The decision on which product or products to use will depend on the cost:benefit ratio, which in turn will be based on access to relevant scientific information and an understanding of the products’ potential role in the diet. Because it is likely that multiple products will be utilized in the diet concurrently, there is a pressing need to evaluate the impact of blends of such products (Hutchens et al., 2021); some feed additives may antagonize the functioning of other products added to the feed. Yet, there is very little information on this topic in the literature at the present time.

One of the most common flaws inherent in studies of such feed additives is incomplete information on the genetics of the pigs being used, their vaccine and medication history, their health status during the study, the composition of the test diets, and a clear description of the experimental design and environmental conditions within the research facility (Bedford and Masey O’Neill, 2016; Olsen et al., 2018). These factors can clearly impact the outcome of the evaluation, make it difficult to interpret the results, and certainly impede the ability of researchers and commercial nutritionists to compare study outcomes.

#### Antibacterial metals and nanoparticles.

Both zinc and copper are known to possess antimicrobial capabilities, and are therefore widely used in the diets of young pigs when digestive disturbance is a frequent occurrence or a significant risk. Along with silver, zinc, and copper are known to generate active oxygen species, interfere with bacterial protein functions, and damage bacterial DNA (Xiu et al., 2014; Rosen, 2002). Højberg et al. (2005) suggested that both zinc and copper may reduce the microbiota in the small intestine, protecting certain nutrients from fermentation so they can be absorbed as intact nutrients, a rout that is recognized as more efficacious, as opposed to being fermented later in the intestinal tract to short-chained fatty acids. Zinc has other effects as well, such as supporting a more effective intestinal barrier function (Skrovanek et al., 2014) and improved overall epithelial architecture in the small intestine (Lee et al., 2016; Wei et al., 2021b).

Both zinc and copper are included in diets at nutritional levels—those required to satisfy minimum nutrient requirements—but may also be added at supra-nutritional levels—those employed to provide protection against select pathogens in the pig’s environment. The latter is sometimes referred to as pharmacological levels. Zinc at so-called supra-nutritional doses has proven to be particularly effective against colibacillosis. To maximize antimicrobial benefit, zinc is frequently added to the phase 1 nursery diet at 2,500 to 3,000 mg zinc/kg of diet as ZnO for the first week to 10 d post-weaning. In North America, zinc is typically fed for another 1 to 2 wk at 1,500 to 2,000 mg zinc/kg of diet. Feeding beyond 21 d is not recommended, as the performance of the pigs may start to decline. More recently, other sources of zinc are being investigated to achieve similar health and growth benefits with reduced impact on the environment.

In some parts of the world, notably the European Union, zinc at supra-nutritional levels has been banned as of 2022. Part of this was motivated by environmental protection, due to fears of zinc accumulation in the soil as a consequence of elevated zinc in the manure. Recently, research in Germany has revealed that high levels of zinc in the diet can lead to *E. coli* that are resistant to such antimicrobials as tetracycline and sulfonamide (Bednorz et al., 2013; Vahjen et al., 2015; Ciesinski et al., 2018). Interestingly, the development of antimicrobial resistance was either absent or very small after 14 d of exposure to zinc, but extending the feeding period to 28 d clearly resulted in the development of resistance. Slifierz et al. (2015) surveyed 26 farms in southern Ontario and observed that the use of zinc as ZnO at levels above 2,000 ppm was associated with the presence of methicillin-resistant *Staphylococcus aureus*; it was not detected in any herd using zinc as ZnO at levels below 2,000 mg/kg. There is some suggestion that following the removal of ZnO from the diet, genes coding for antimicrobial resistance may decline and even return to baseline; this is a topic requiring additional research. A recent study reported by Kansas State University has challenged the whole concept of microbial resistance arising from the use of supra-nutritional levels of zinc, since they found no evidence of this effect (Chance et al., 2021).

It has also been noted that supra-nutritional levels of zinc may impair the effectiveness of phytase releasing phosphorus from phytate, although not all studies have reported this observation (Augspurger et al., 2004).

Looking to the future, there is a growing body of knowledge demonstrating that zinc oxide provided as nanoparticles at lower concentrations may achieve equal or similar outcomes in terms of animal performance and health (da Silva et al., 2019). For example, Pei et al. (2019) reported that 300 mg Zn/kg in the form of nanoparticles achieved improvements in rate and efficiency of gain, as well as in the incidence of diarrhea as 3,000 mg Zn/kg provided in conventional form. However, not all studies have achieved such success. Encouraging results have also been reported with zinc oxide encapsulated in a lipid matrix to reduce the total quantity of zinc used while achieving similar antimicrobial activity (Kim et al., 2015). Finally, organic forms of zinc, such as those complexed with amino acids, have also been considered as a means of reducing total zinc addition to the diet, while maintaining desired performance and health outcomes. Results have been variable in studies of alternative sources of zinc; some studies have been very encouraging and others less so. Nonetheless, the ability to achieve similar outcomes at lower zinc levels is encouraging from an environmental perspective. Therefore, continued research into alternative forms of zinc is encouraged.

Supra-nutritional levels of copper, between 125 to 250 mg/kg, are most frequently used in nursery diets to help control diarrhea as well as improve growth performance (Bikker et al., 2016) In North America, 250 mg copper per kg of diet as copper sulfate may be fed in the phase 3 diet (days 21–42), when zinc is no longer employed, for about 3 wk. In cases where ZnO is not permitted, copper may be used in phase 1 and 2 diets as well. Tribasic copper chloride, providing the same level of copper in the diet as copper sulfate, has been shown to provide equivalent benefits to growth performance (Cromwell et al. 1998). Copper is used much less frequently in grow-finish diets due to cost and a generally muted response. Like zinc, copper has been shown to also contribute to antimicrobial resistance.

#### Bacteriophages.

Viruses that attack bacteria, or bacteriophages, were discovered in the early 20^th^ century and quickly earned interest as a possible treatment for bacterial infections (Weinbauer, 2004). Phages exist in plentiful supply in nature and are found in water, soil, and foods. In humans and animals, they have been found on the skin, as well as in the GI tract, the lungs and urinary tract (Van Belleghem et al., 2019). They are known to interact with the mammalian immune system in both direct and indirect ways. Given their natural presence, they are considered safe for use in animal and human treatment. Indeed, early data have confirmed the safety of phage therapy (Klopatek et al., 2021; Thanki et al., 2021), but further work will be required to provide a more complete portfolio of safety documentation for specific products utilized in specific ways.

Phage “therapy” failed to live up to early expectations, and with the later discovery of antibiotics, interest in the topic flagged. However, with the high cost incurred in the development of new antibiotics, combined with the growing concern about AMR, interest in phage therapy has been revived. Phage therapy has some advantages and disadvantages compared to antibiotics. For example, phages tend to be active against only a single bacterial species, while antibiotics have a broader impact. This may be advantageous since other bacterial species remain unaffected, leading at least theoretically to fewer secondary effects of treatment; however, it may also be disadvantageous since the target bacteria must be clearly identified prior to treatment. This greater priority placed on diagnostics may delay the onset of treatment, or lead to errors in treatment (Sulakvelidze et al., 2001). Some phages, called lytic, are considered to be strictly virulent, killing their target bacteria without otherwise interacting with the target; others are called temperate, with the ability to insert their genomic material into the host DNA and thus potentially alter the bacteria in some manner which might not be advantageous to the infected animal. Because of this ability, temperate phages are not used for therapeutic purposes (Kahn et al., 2019). There is also the risk of animals developing resistance to specific strains of phages after repeated use, so this will have to be carefully managed to maintain efficacy (Johnson et al., 2008).

Phages have been studied in swine with highly variable results in the treatment of *Salmonella* spp. (Callaway et al., 2011) and *Escherichia coli* (Jamalludeen et al., 2009). Phages are not yet available for administration to pigs during production, but they are already contributing to food safety programs through post-harvest applications against *Salmonella*; a number of such products have received provisional Generally Recognized as Safe (GRAS) status from the U.S. Food and Drug Administration (Kahn et al., 2019).

There are practical issues to be addressed before commercial use of phages will occur. Apart from achieving more consistent experimental results, information on appropriate doses is required as well as development of the best route of administration. Because phages are not stable at low pH, they may have to be either encapsulated to bypass the stomach if administered through feed or water, or inoculated; they are also susceptible to the action of bile (Johnson et al., 2008). Suffice it to say that much more research is required before phages can achieve their full potential as a tool for nutritionists and veterinarians to utilize in the pork industry in support of ABF production. Nonetheless, it is an area of great interest, due to the potential phages could bring to pork production if these practical and technical issues can be adequate addressed (Desiree et al., 2021).

#### Direct fed microbials (Probiotics).

Under ingredient definition T36.14, the Association of American Feed Control Officials defines direct-fed microbials as feed products that contain live (viable) naturally occurring microorganisms. The organisms must present no safety concerns and must be non-toxigenic (AAFCO, 2018). Therefore, in the United States, products destined for use in animal feeds are called direct-fed microbials; such products directed toward the human food market are referred to as probiotics. Elsewhere in the world, the term probiotic applies to both animal feed and human food products. The FAO/WHO defines probiotics as “live microorganisms that, when consumed in adequate amounts, confer a health effect on the host” (Hill et al., 2014).

To be effective, direct-fed microbials must obviously contain live organisms which have the ability to endure feed processing conditions and storage before being fed, and following consumption, survive the acidic environment of the stomach and then colonize the GI tract. In so doing, they must be able to suppress the proliferation of enteric pathogens. Lactobacillus, Bifidobacterium, Enterococcus, and Bacillus are the most commonly used genera of bacteria in direct fed microbials; all are classified as members of the firmicute phyllum. The first three are notable in that they are lactic acid producing, which is a highly desirable trait in competing against pathogenic bacteria. However, they are also non-spore-forming, so their physical and biological stability is less certain. Bacillus, on the other hand, is spore-forming, conferring on them a natural stability that is favorable under the conditions experienced during feed production and storage.

Within a given direct-fed microbial product, it is common to find more than one species of bacteria within a given genus, and sometimes more than one genus is also represented (Lambo et al., 2021). Also, not all DFMs contain bacteria; some may include yeast or fungal products, such as fungi of the genus Aspergillus or yeast of the genera Saccharomyces (Liao and Nyachoti, 2017). Barba-Vidal et al. (2018) undertook a thorough review of the literature on probiotics published between 1992 and 2017; the vast majority of the 31 total papers were published since 2010. Of this total, 26 reported at least some positive results, generally in some aspect of immunology, but very few described benefits in terms of growth performance. The authors noted that this summary could include some degree of publication bias, due to the difficulty of publishing neutral results.

The benefit of utilizing direct-fed microbials in ABF production systems may accrue from one or more of a variety of possible outcomes. These include effective direct competition against pathogens resident in the GI tract, improvements in gut barrier function, increases in diet digestibility and faster and more efficient growth. It is believed that direct-fed microbials may prevent adhesion of pathogens to the intestinal epithelium through competitive exclusion or steric hindrance (Li et al., 2003; Roselli et al., 2005). Organisms commonly used in direct-fed microbials secrete various compounds which may provide benefit to the pig; this may include short-chain fatty acids, colicin, and bacteriocins. A review of the literature fails to identify a single outcome or even group of common outcomes associated with the use of direct-fed microbials. While this may be due to the wide array of probiotics being investigated, it is probably also due to such research variables as the composition of the basal diet, the health status of the research herd, the length of feeding of the product, the organisms present in the direct-fed microbials, and the social and physical environment within the research facility. Research on direct-fed microbials would benefit greatly from standard experimental quality assurance practices such as measurement of viable organisms in the feed at the time of presentation to the pig. While there can be many explanations for a lack of product response, improper feed handling, which destroys live bacteria is a particular concern.

In addition, information on the presence and risk associated with antibiotic-resistant genes (Shridhar et al., 2022) and in some cases antibiotic-resistant phenotypes (Amachawadi et al., 2018) in direct-fed microbials should be noted. Reporting information on virulence characteristics and antimicrobial resistance of a commercial probiotic is required per guidelines developed by the Food and Agriculture Organization-World Health Organization (FAO/WHO, 2002).

#### Enzymes.

Enzymes are commonly added to the diets of pigs to enhance their innate digestive capacity. This may be achieved by helping to release nutrients entrapped within the fiber matrix present in plant cell walls, by reducing the loss of nutrients that occurs due to fermentation, improving the utilization of sugars present in polysaccharide chains, by producing beneficial fiber hydrolysis products, by enhancing gut barrier function in the small intestine, by reducing endogenous losses occurring as a result of the sloughing of epithelial cells or by reducing the impact of antinutritional factors present in the diet (Patience and Petry, 2019). In terms of sheer market penetration, phytase is the most frequently used enzyme in pig diets. However, there are numerous carbohydrases in use as well, including xylanase, β-glucanase, β-mannanase, and cellulase. Proteases round out the family of exogenous enzymes most commonly used in practical pig diets.

While these enzymes are typically viewed as functioning to increase nutrient utilization by the pig, recent research has revealed another potential role—improving pig health and reduction in mortality. For example, Li et al. (2018) and Tiwari et al. (2018) reported that a blend of carbohydrases reduced the urinary ratio of lactulose to mannitol and increased the mRNA abundance of claudin-3, occluding, and zona occludens-1, indicative of enhancement in small intestine barrier integrity. Li et al. (2019) observed that carbohydrase blends reduced markers of gut and systemic inflammation coinciding with improved growth performance in newly weaned pigs. Improved barrier function and reduced systemic and tissue inflammation may explain observations by Boyd et al. (2019) that xylanase inclusion in the diet of growing and finishing pigs reduced mortality.

Carbohydrases may provide health benefits through another mechanism. These enzymes have the ability to cleave polysaccharide chains, thus forming oligosaccharides, which are carbohydrates with chain lengths of fewer than 20 sugars. These enzyme breakdown-products appear to provide benefit in the instance of a K88 (F4) enterotoxigenic *E. coli* (ETEC) challenge (Kiarie et al., 2009). Carbohydrases, in the presence of soluble fiber, appeared to also provide benefit in the presence of an F18 ETEC challenge, including reduced immune activation, enhanced gut barrier function, increases in *Lactobacillus* and decreases in *Escherichia* and *Shigella* (Li et al., 2019; González-Ortiz et al., 2014; Chen et al., 2012). More research is required to craft a well-defined strategy to apply this technology in the field in ABF production systems, but the technical foundation appears to be sound.

Proteases may provide another benefit associated with improved nutrient digestibility. It relates to the enhancement of digestion of dietary protein so that it is utilized as a source of amino acids or energy in the small intestine; if this is the case, then improvements in intestinal health could occur from a reduction in the fermentation of protein in the lower gut. As on example, Perez-Palencia et al. (2021) reported that protease included in the diet reduced diarrhea on days 7, 10, and 14 post-weaning, although this did not translate into improved pig growth performance.

#### Fermenting ingredients and feed.

Fermenting feed or ingredients is not an additive per se, but it represents a process that can be used to positively affect pig health and performance, through enhancement of the microbial population within the GI tract. And of course, there are materials which have been fermented industrially, dried and sold to the pork industry for use in diets. Ingredients which are best suited to fermentation are probably those which are rich in simple carbohydrate, such as starch, to support the process, but any carbohydrate source will work. These fermented ingredients are then added to the feed, using a liquid feeding system if necessary.

Fermentation of diets prior to feeding has the potential to improve growth performance and decrease mortality and morbidity (Braun and de Lange, 2004). These benefits are believed to accrue from a combination of factors, including increased energy and nutrient digestibility, suppression of pathogenic bacteria and their negative impact on gut function, enhanced gastric function accruing from lowered pH, and the provision of lactic and organic acids. However, depending on the conditions of fermentation, it may result in adverse outcomes due to reduced feed efficiency if excess fermentation consumes too many nutrients, production of amines which are highly unpalatable and accrue from protein fermentation, and generation of acetic acid, which is also unpalatable. Given the above, it is not surprising that the benefits of fermentation on performance are highly variable and speak to the need to understand and control the process very well (Plumed-Ferrer and Von Wright, 2009).

Fermenting feed requires mixing with water in a ratio of approximately 2 to 3:1 (Plumed-Ferrer and Von Wright, 2009) and storage for approximately 4 d prior to delivery to the pigs in liquid form. The objective of fermentation is to lower the pH of the feed—typically to between 3.5 and 4.5—through the production of lactic acid and possibly acetic acid, elevating numbers of lactic acid producing bacteria and lowering numbers of *Enterobacteriaceae*. Yeast fermentation should be avoided due to the production of compounds that reduce feed intake; the use of warm rather than cold water will help in this regard (Plumed-Ferrer and Von Wright, 2009). Organic acids may be added to the mixture to ensure achievement of the desired final pH of the mixture.

Feed fermentation is believed to improve its palatability, but this has not been consistently achieved. Fermentation may be beneficial in controlling *salmonella*; the addition of 50 ppm copper as the pentahydrate to the mixture has proven to be helpful in this regard (Beal et al., 2004). Fermented diets are believed to provide some degree of protection against pathogens, possibly through the reduction in gastric pH and/or beneficial changes in the architecture of the gastrointestinal tract.

Fermentation appears to be a two-part process; during the first phase, pH is above 4.5 and *Enterobacteriaceae* dominate but in the second phase, pH declines and lactic acid bacteria dominate (Canibe and Jensen, 2003). Many factors such as feed composition, ambient temperature, water temperature, and water quality will influence the length of the first phase as well as the total length of time required to extend fermentation to achieve the desired final pH and bacterial profile. These are critical factors in determining the success of feed fermentation. The possibility of unfavorable fermentation exists, so the process must be carefully monitored. Inoculants might be used to achieve more control of the fermentation process, but the regulatory status of this practice is uncertain at the present time (Plumed-Ferrer and Von Wright, 2009).

#### Lysozymes.

Lysozymes, also called 1,4-β-N-muramidase, is a special group of enzymes (EC 3.2.1.17) with the ability to cleave the β-(1,4)-glycosidic bond, which link N-acetylmuramic acid and N-acetyl-glucosamine in peptidoglycan. Peptidoglycan happens to be a major component of the cell wall of bacteria; disruption of its integrity leads to lysis of the cell and subsequent death. Interestingly, the products of bacterial cell wall hydrolysis stimulate the secretion of immunoglobulin A, activates macrophages, and increases the speed of bacterial pathogen clearance (Oliver and Wells, 2015). Lysozymes are ubiquitous in nature and exist in many different types, but it appears that all have antimicrobial activity; some have digestive functions as well. Gram-negative bacteria have a natural defense against lysozymes, namely a surrounding outer layer rich in lipopolysaccharide. Lactoferrin, cathelicidins, and defensins, all part of the animal’s innate immune system, are able to rupture this membrane, exposing it to the action of lysozymes (Callewaert and Michiels, 2010; Cooper et al., 2013).

Lysozyme is abundant in egg whites and mammalian milk, but for unknown reasons, is present at low concentrations in sow’s milk; it can also be found in tears and saliva. However, egg white lysozyme has quite different properties compared to the milk form; the former tends to be more stable at higher temperatures and has a wider pH optimum. For reasons which are not yet clear, human milk is a much, much richer source of lysozyme than milk from livestock; it is also much more active than the lysozyme present in egg whites. Lysozyme harvested from egg whites is considered the most promising for use in swine diets (Oliver and Wells, 2015).

These properties and functions of lysozyme have naturally led to interest in their use as an alternative to antimicrobials, particularly in the diet of the newly weaned pig, which is so susceptible to colibacillosis. Indeed, early research has demonstrated favorable changes in the intestinal microbiota and the architecture of the gastrointestinal tract (Brundige et al., 2008) as well as providing protection against enterotoxigenic *E. coli* (Garas et al., 2017) and a reduction in inflammation (Nyachoti et al., 2012). Improvements in pig performance have been reported, in some cases—but not always—equal to that of pigs fed antibiotics (May et al., 2012; Oliver and Wells, 2013; Oliver et al., 2014; Zou et al., 2019). In other instances, a performance benefit was not observed at all (Nyachoti et al., 2012).

Lysozyme is produced commercially and has been widely used for more than 30 yr as a preservative in foods and pharmaceuticals. In some countries, it is reported to be a popular medicine employed in the treatment of such maladies as colds and sore throats. It is even used in organic wines, in place of sulfite, and in non-pasteurized beer. It is a relative newcomer to the world of non-antibiotic feed additives in swine diets. Larger scale production and refinement in production and harvest procedures will no doubt lower the cost, allowing lysozyme to become a competitive alternative to antibiotics.

#### Medium-chain fatty acids and medium-chain triglycerides.

 Medium-chain fatty acids are those which are saturated monocarboxylates with 6 to 12 carbons, including the 6-carbon caproic acid or hexanoic acid, 8-carbon caprylic or octanoic acid, 10-carbon capric or decanoic acid, and 12-carbon lauric or dodecanoic acid. Some authors do not consider caproic or lauric acid as medium-chain fatty acids. They occur in nature in the form of triglycerides in milk and various vegetable fats such as palm or coconut oil. These products are of interest in ABF production systems 1) as a highly available source of energy, helping the young pig successfully transition at the time of weaning and possibly providing growth-promoting benefits, 2) as an antimicrobial in the diet of the pig, 3) as compounds which help to maintain favorable intestinal architecture, and 4) as a means of controlling pathogens in the feed such as African Swine Fever virus, Porcine Epidemic Diarrhea virus, and *Salmonella* (Zentek et al., 2011). This latter role will be addressed later in this manuscript.

From a nutritional perspective, the advantages of these products over longer chain triglycerides are their relative ease of digestion, their passive absorption directly into the portal vein and obligatory oxidation in the liver, thus providing a highly available source of energy for the young pig (Odle, 1997). As ideal as medium-chain fatty acids may appear for the young pigs, it should be noted that sow’s milk is considered a very poor naturally occurring source. While medium-chain fatty acids and monoglycerides may be fed as such, they are frequently included in the diet as medium-chain triglycerides; depending on the situation, such as the age of the pig, lipase may be included with the medium-chain triglycerides to ensure rapid hydrolysis to medium-chain fatty acids and monoglycerides (Jackman et al., 2020). One advantage of feeding medium-chain triglycerides is the avoidance of concerns regarding palatability and off-odors, frequently identified as a concern with some medium-chain fatty acids and thought to result from rapid oxidation when the free fatty acid is not bound to a glycerol skeleton (Decuypere and Diericks, 2003).

Some researchers have reported improvements in the growth performance of newly weaned pigs when medium-chain fatty acids or medium-chain triglycerides are added to the diet; for example, Gebhardt et al. (2020a) reported improvement in feed efficiency but not in growth rate when individual or blended C6:0, C8:0, and C10:0 were fed for the first 2 wk post-weaning. The typical rate of addition of individual medium-chain fatty acids has ranged from 0.2% to 0.5% and of MCT has ranged 1.2% to 4.8%. Higher levels have been employed, but the results have been disappointing (Allee et al., 1972). Overall, their impact on the performance of young pigs has been inconsistent, and more research in vivo is required, especially under commercial conditions (Zentek et al., 2011; Hanczakowska, 2017). Feeding medium-chain triglycerides to pregnant sows has been proposed as a strategy to improve livability in gilt offspring; the most recent research suggests this is not a likely outcome, at least not under the conditions of this particular trial (Craig et al., 2019).

Medium-chain fatty acids possess bacteriostatic, bactericidal, antiparasitic, and antiviral activity. They have been shown to inhibit a wide array of pathogens, including both Gram-negative and Gram-positive bacteria, along with algae, fungi, protozoa, and certain viruses (enveloped viruses). It appears that the monoglycerides of capric and lauric acids have the greatest antimicrobial activity, although the free fatty acids are also effective (Jackman et al., 2020). It is important to note that there is a degree of specificity towards specific pathogens by various medium-chain fatty acids and their monoglycerides, so this needs to be considered in the adoption of a medium-chain fatty acids-based antimicrobial treatment program. Chemically, the undissociated forms of these fatty acids will express the highest degree of antimicrobial activity, although the pH of the intestinal environment will impact the degree of activity of the dissociated acid (Eklund, 1983; Rossi et al., 2020). Due to their size and chemical composition, medium-chain fatty acids are able to penetrate the semi-permeable cell membrane of bacteria; once inside, they lower the pH of the cytoplasm, impairing metabolic enzymes and nutrient transport systems, thus leading to death. They may also function by cleaving the plasma membrane, leading to cell death (Jackman et al., 2020). Acid-producing bacteria such as Lactobacilli do not appear to be affected in the same way, probably due to their lower cytoplasmic pH, and therefore escape the bacteriostatic and bactericidal outcomes. It should also be noted that medium-chain fatty acids and their monoglycerides may benefit the pig through modulation of the immune system and also through changes in the microbiome.

Given the antimicrobial and immunological modulatory capabilities of medium-chain fatty acids, their monoglycerides and medium-chain triglycerides, this group of compounds may prove to offer a very useful role in ABF production systems. However, it is important to note that medium-chain fatty acids and the monoglycerides are much more potent bacteriostatic and bactericidal compounds than medium-chain triglycerides; this is why lipases may be added when medium-chain triglycerides are used, especially in the young pig, because they catalyze the conversion of the triglyceride to free fatty acids and monoglycerides (Jackman et al., 2020). Further development of this technology, including appropriate dosing and delivery systems, enhanced understanding of individual fatty acid specificity for certain pathogens, evaluation under commercial pork production conditions, and more efficient production technologies to reduce cost, will help to more solidly secure their position in the pork industry of the future.

#### Nucleotides.

Nucleotides are organic molecules that are naturally found within the body of all living things. This is because they form the foundational structure of all RNA and DNA. Nucleotides also play a central role in numerous metabolic processes; for example, the so-called “energy currency” of the body includes one of the nucleotides, adenosine in adenosine triphosphate (ATP). They are an important part of cell signaling mechanisms which include, as one example, cyclic adenosine monophosphate (cAMP). Finally, they are incorporated into coenzymes such as NAD, NADPH, and FAD.

Because nucleotides contain nitrogen, they are included in crude protein assays of ingredients and feeds, because the procedure does not distinguish between nitrogen in protein and non-protein nitrogen. The crude protein content of many ingredients is therefore over-estimated by as much as 10% to 25% due in part to the level of nucleotides and other non-protein nitrogen present (Patience et al., 1995).

At the present time, supplementing nucleotides is proposed for the diets of the young newly weaned pig. The origin of this concept is difficult to pinpoint, but it appears to be based on the fact that sow’s milk contains substantial levels of nucleotides. It is assumed that milk contains nucleotides because they are required in the diet, and the levels of nucleotides in milk can be used to estimate this requirement. The major roles of nucleotides were previously described, but they also enhance antibody responses, contribute to iron absorption and are involved in polyunsaturated fatty acid synthesis in the young pig (Schlimme et al., 2000). This has led some people to consider nucleotides a conditionally essential nutrient in the diet of the young pig. Various studies in the human infant have suggested that nucleotide supplementation optimizes the growth of tissues of the GI tract. They may also enhance cellular and humoral immunity, especially after an injury. Nucleotide supplementation may also enhance the profile of the microbiome in the young pig, but that requires further research and confirmation. While nucleotides can be supplemented in purified form, they can also be provided as constituents of yeast extracts at a much lower cost.

Achieving measurable benefits in response to nucleotides in the diets of newly weaned pigs has been challenging. This may be due to their essentiality only during periods of excessive stress or tissue injury, or it may be related to the fact that enzymes involved in nucleotide catabolism are present in significant quantities in the gastrointestinal tract (Carver and Walker, 1995). Perhaps nucleotides are best supplemented in the diet of the young pig when GI damage has occurred, or other insult which places greater demands on nucleotide synthesis; in any event, much more research is required on this topic.

#### Organic acids and their salts.

Extensive reviews have been published on the use of OAs and inorganic acids, and their salts in pig diets (Suiryanrayna and Ramana, 2015; Nguyen et al., 2020; Tugnoli et al., 2020). The expected benefits of organic acids stem from one or more of six proposed modes of action: 1) lowering the rate of stomach emptying, 2) modification of antimicrobial activities, 3) lowering digesta pH, 4) stimulating pancreatic enzyme production, 5) enhanced nutrient digestibility, especially protein, and 6) direct provision of nutrients to enterocytes (Partanen and Mroz, 1999; Pettigrew, 2006; de Lange et al., 2010; Nguyen et al., 2020). However, these potential beneficial effects are not always realized, possibly because of the age or health status of the pig, the nature and concentration of organic acids employed or the composition of the diet, including its acid-binding capacity. For example, some diets, notably those high in calcium carbonate, will possess a substantial degree of acid-binding capacity, which could counter the acidifying impact of organic acids (Blank et al., 1999; Hajati, 2018). Some formulations will stimulate acid secretion in the stomach on their own, potentially obviating the value of more acid being added to the diet. It is also possible that the acid is metabolized or absorbed in the duodenum, resulting in minimal benefit in the jejunum and ileum, where lowering the pH may provide benefits to the pig challenged by pathogens favoring a high pH environment. To counter this, organic acids are sometimes encapsulated in a lipid matrix as a means of delaying the release and absorption of acids, so they exert their impact lower in the gastrointestinal tract (Piva et al., 2007; Upadhaya et al., 2014).

The most common organic acids in commercial use today include butyric, formic, propionic, lactic, citric, acetic, fumaric, malic, and sorbic acids, as well as their salts (e.g., calcium formate, calcium propionate). However, other acids and their salts are earning commercial attention as well. Many commercial products contain a blend of organic acids, or organic acids and their salts; the approach seeks to achieve more consistent microbial control and metabolic impact, possibly due to the synergism of their differing pK_a_ values. In some instances, a blend of acids with phytogenic compounds or other additives is sold to achieve the complementary benefits of the two classes of compounds (Choi et al., 2020).

Organic acids may be classified into three main structural categories: short-chain carboxylic acids with a maximum of 5 carbons, medium-chain fatty acids possessing aliphatic chains of 6 to 12 carbons, and tricarboxylic acids, which are best known as intermediates of energy metabolism (Tugnoli et al., 2020). The short-chain fatty acids—acetic (pK_a_=4.7), propionic (pK_a_ = 4.9), and butyric (pK_a_ = 4.8)—may be produced by microbial fermentation in the lower gut; butyric acid is known as an important energy source for enterocytes. In vivo production of these short-chain fatty acids lowers the pH of the contents of the large intestine and cecum, thus inhibiting proliferation of pathogenic bacteria, which tend to prefer a higher pH environment. Formic acid, with only one carbon, is also considered a short-chain fatty acid and has a pK_a_ of 3.8. The medium-chain fatty acids are more effective as antimicrobials due to their pK_a_ [caproic (5.1), caprylic (4.9), capric (4.9), and lauric (5.3)]. Since the undissociated form of the acid is a more effective antimicrobial, a pK_a_ between 3 and 5 is desired (Partanen and Mroz, 1999). The tricarboxylic acids include citric (C6; pK_a_ = 3.1, 4.8, 6.5), fumaric (C4; pK_a_ = 3.0, 4.4), and malic acid (C4; pK_a_ = 3.4, 5.1). Beyond these three categories, a few other relevant OA exist which are used in pig diets, often for their antifungal properties. These include lactic (C3; pK_a_ = 3.8), benzoic (C7; pK_a_ = 4.2), and sorbic acids (C6; pK_a_ = 4.8). Salts are sometimes preferred over their corresponding free acid since they are typically odorless, less volatile, more soluble in water and less corrosive; they are thus easier and safer to handle (Tugnoli et al., 2020).

As previously stated, one theory on the importance of organic acids in the diet is to lower the pH of the stomach. The newly weaned pig is at particular risk of a high gastric pH due to an immature acid-secreting capacity as well as a lack of lactic acid, derived from sow’s milk prior to weaning. The composition of the diet of the young pig may also contribute to this elevated gastric pH. The net result is impaired functioning of pepsin, leading to reduced initial digestion of proteins in the diet, leading to more rapid emptying of the stomach, which places pressure on the digestive capabilities of immature duodenal activity. Organic acids are thus added to the diet of the young pig to counter these challenges to the developing gastric competency. However, this largely theoretical explanation of the role of organic acids may not be achieved in practice. Pettigrew (2006) reported that while organic acids lowered the pH of the feed in 98% of reported studies, they only lowered the pH of the contents of the stomach and small intestines in about 55% of the studies, of the large intestine in 36% of the studies, and of the colon in 72% of the studies. Other than feed, none of these observed differences achieved statistical significance (*P* > 0.10). Pettigrew (2006) concluded that the benefit of organic acids was more likely related to improvements in nutrient digestibility and changes in microbial populations, than to the alteration of the pH of the gut contents.

Ultimately, organic acids are fed to pigs to provide protection against pathogens and to enhance growth performance. It is therefore important to know if the product, applied as directed, will have bacteriostatic or bactericidal effects in the gut. As one example, Zentek et al. (2013) reported that a combination of organic acids and medium-chain triglycerides resulted in no benefit in terms of growth performance, but clear benefits in terms of the microbial ecology in the gut. In a novel study conducted at two American universities, Wei et al. (2021a) reported that a combination of benzoic acid and differing levels of sodium butyrate improved growth rate in one location and not the other. There were indications of a positive effect on the microbiota as determined in fecal samples. Overall, the literature suggests that there is considerable variation in the performance response to organic acids, but in general terms, the benefit is greatest in the young pig and declines as the pig grows older (Nguyen et al., 2020). The smallest benefit tends to be observed in pigs in the finishing period (Tugnoli et al., 2020). Typically, organic acids are included in the diet at between 0.2 and 1.5%, whether they contain a single or multiple acids (Nguyen et al., 2020).

#### Plant extracts.

Also referred to as phytogenics, these compounds represent a category of feed additives which include herbs, spices, essential oils, and oleo resins. The active ingredients may be extracted from bark, seeds, leaves, or roots; they may be harvested from plants through cold pressing, steam distillation, maceration, or extracted through the use of nonaqueous solvents (Windisch et al., 2008). As a category of feed additives, they pose unique challenges because they may vary with respect to botanic origin, may be processed in different ways, and may differ in their composition and concentration. Their mode of action is largely unknown or poorly defined, but they are believed to possess antimicrobial, antioxidative, or grow-promoting properties.

Phytogenics derived from plants high in terpenes, such as rosemary, oregano, and thyme, are believed to have antioxidant properties. Anise, coriander, flavonoids, anthocyans, red pepper, and chili are also believed to contain components with antioxidative properties. Since they are antioxidants, they may provide protection against the deterioration in fat quality in pig diets, as well as provide support to other antioxidants in the diet, such as selenium and vitamin E.

It has been known since before the turn of the century that certain herbs and spices possess antimicrobial activity; more recently, bacteriostatic and bactericidal effects along with action against fungi and protozoa have been demonstrated (Franz et al., 2010). Phenolic compounds, such as carvacrol, thymol, and eugenol are of greatest interest in this regard, but other compounds such as phenylpropane, limonene, geraniol, and citronellal also show promise. Their mode of action is not well known, but it has been hypothesized that their lipophilic properties allow them to transverse the microbial membrane, causing lysis and death (Windisch et al., 2008). Effectiveness against *Bacillus cereus*, *Salmonella typhimurium*, *Escherichia coli*, *Listeria monocytogenes,* and *Staphylococcus aureus* appear to be the pathogens attracting the greatest attention in resent research.

Phytogenic compounds may be able to replace some of the growth-promoting benefits of antibiotics as well (Franz et al., 2010; Maenner et al., 2011; Hunger et al., 2017) and provide benefits against *E. coli*-based post-weaning diarrhea (Moran et al., 2020). As seen with other non-antibiotic feed additives, results are inconsistent; there can be many reasons for variable results, but palatability is a topic that needs attention, as some of the products may improve diet attractiveness to young pigs, while others may have the opposite effect. Not surprisingly, beneficial responses appear to be more frequent in studies with young pigs as compared to grow-finish swine (Zeng et al., 2015). The development of a better understanding of the mode of action of such products will be crucial to their gaining better traction within an increasingly sophisticated pig industry. The introduction of new technology into the use of phytogenic products, such as nanotechnology, may also prove advantageous (Omonijo et al., 2018).

#### Prebiotics, stimbiotics, and synbiotics.

Bindels et al. (2015) have proposed the following definition of a prebiotic: “a nondigestible compound that, through its metabolization by microorganisms in the gut, modulates composition and/or activity of the gut microbiota, thus conferring a beneficial physiological effect on the host.” In human nutrition, prebiotics are typically selected on the basis of four criteria. First, they cannot be hydrolyzed by mammalian gastrointestinal enzymes, nor absorbed through the gut, and they are also resistant to gastric acidity. Second, they must be selectively fermented by one or more beneficial bacteria in the colon. Third, they must alter the colonic microbial population towards what is considered to be a healthier composition. Finally, prebiotics should ideally provide health benefits to the host (Gibson, 1999).

According to the above, good candidates for prebiotic status in swine diets would be inulin with 2 to 60 degrees of polymerization and its hydrolytic product, oligofructose, with 2 to 20 degrees of polymerization. Inulin is present in such feedstuffs as Jerusalem artichoke, agave, and chicory roots; agave inulin is a highly branched form of inulin which begins to be fermented in humans about 4 h after ingestion, with peak fermentation occurring within 6 h, while chicory inulin is more linear and less branched, so it achieves peak fermentation in the human gut about 8 h after ingestion (Holscher, 2017). These fructooligosaccharides, along with the closely related galactooligosaccharides; sometimes referred to as trans-galactooligosaccharides, meet all the criteria for classification as prebiotics. However, other ingredients in the diet of the pig may satisfy one or more of the above criteria, and thus provide benefits to the host. This list might include soybeans and oats (Pandey et al., 2015); even breakdown products of enzymes appear to possess some functions similar to prebiotics, as shown in recent studies by Petry and Patience (2020). These are sometimes referred to as stimbiotics (González-Ortiz et al., 2019).

There is likely to be considerable benefit if success can be achieved in managing the microbial population of the gut in the same way that we control other aspects of the pig’s growth and metabolism. Part of the challenge in achieving this is the wide array of factors that influence the gastrointestinal microbiota. These include such things as the genetics of the pig, the age of the pig, its health status, the degree of stress under which it lives, its physical environment and, of course, its diet (Holscher, 2017). Our understanding of the role of diet on the gastrointestinal microbial population is advancing at an increasingly rapid pace, so there is justification for optimism in our being able to manage it more effectively in the future than we have in the past. With this expanding knowledge comes an understanding that specific changes in the diet, through the addition of prebiotics or probiotics, may provide an opportunity to reduce our dependence on antibiotics for the control of certain pathologies of the gut. Prebiotics are certainly one of the tools available to achieve this highly desirable outcome.

Historically, the focus of most research in the previous century was on bifidobacteria, eubacteria, and lactobacilli. With the advent of more rapid and less costly molecular methods to characterize the microbiota, the list of target organisms has grown substantially. For example, *Faecalbacterium prausnitzii*, a butyrate-producing bacteria, and *Akkermansia muciniphila*, a mucin degrading bacteria, have attracted more recent attention (Holscher, 2017).

Prebiotics have an advantage over direct-fed microbials. As live microbes, direct-fed microbials must be able to survive the conditions which exist in the pig’s upper gut, such as the acidic conditions of the stomach as well as the actions of pancreatic secretions in the proximal small intestine in order to survive to reach the lower gut, where they can become established and proliferate. As a non-digestible feedstuff or feed component, prebiotics would be expected to have a greater chance of surviving the rigors of the upper gut in order to support proliferation of commensal bacteria. On the other hand, achieving the objective of a prebiotic, that is to say stimulating the proliferation of a specific, beneficial bacterium or a group of bacteria to achieve a favorable health outcome, can be highly challenging, given the high degree of variation which exists within the GI tract of the pig.

Synbiotics represent blends of direct-fed microbials and prebiotics (Gibson, 1999; Heo et al., 2013). Synergistic synbiotics represent a combination of a prebiotic and direct-fed microbials that together promote the growth of a specific bacteria; conversely, a complementary synbiotic contains a direct-fed microbials and a prebiotic with differing targets, but which seek to achieve a common beneficial outcome in the overall microbiome.

#### Resistant starch.

Resistant starch is an example of the category of ingredients that act as prebiotics, previously mentioned. Due to their chemical structure, portions of starch found in many common cereal grains, legumes, and root crops escape enzymatic digestion in the upper gut and are fermented in the distal small intestine and colon. It appears that resistant starch alters the microbiota and that specific phyla can be associated with specific types of resistant starch. Resulting fermentation enhances the production of short-chain fatty acids, notably butyrate. This is significant because butyrate is a preferred fuel used by colonocytes and other intestinal cells. This enhanced fermentation also has the effect of lowering pH of the intestinal contents, encourages the proliferation of colonic and cecal cells, ameliorates mucosal inflammation and oxidative status, strengthens the epithelial defense barrier, and may even impact gut motility (Canani et al., 2011; Regassa and Nyachoti, 2018; Trachsel et al., 2019; Tan et al., 2021).

The proportion of starch that is resistant to intestinal enzymes is methodologically challenging to measure, but is estimated to be in the range of about 5% for common cereal grains such as wheat, corn, and barley. However, this can be influenced by feed processing, the age of the pig and the dietary source being evaluated. For example, 50% to 60% of starch in potato is fermented (Gerrits et al., 2012).

There are five different categories of resistant starch (RS1 to RS5), some of which occur in nature and others which are produced by man-made activities. RS1 is typically found in grains and legumes such as field peas and low amylose barley and corn; it survives digestion due to the physical barrier effect of cell walls and protein matrices which entrap the starch, rendering it inaccessible to digestive enzymes. RS2 is found, for example, in unripe bananas, raw potatoes, hulless barley, and high amylose barley and corn, and avoids digestion due to its crystalline structure. RS3 is produced when starchy foods such as potatoes and pasta are heated and then cooled, creating double helices within the amylopectin, which then cannot be hydrolyzed. RS4 is formed by chemical processes which occur during processing such as esterification. RS5 has been identified more recently than the other four and is frequently referred to as amylose-lipid complexes (Lockyer and Nugent, 2017).

Based on existing literature, resistant starch should probably not be fed with the objective of improving growth performance, at least not until more research is conducted and knowledge is gained on its application in swine diets. Rather, it should be looked upon to provide benefit to gut function, physiology, and structure, and thus overall pig health (Regassa and Nyachoti, 2018). Performance has been improved in some instances (Krause et al., 2010), but most frequently not improved or potentially even impaired (Olsen et al., 2018). It is possible that a lack of benefit in terms of growth performance from the use of RS could be attributed to its glycemic and insulinemic impact, leading to impaired appetite and reduced feed intake. Taking a different tack, Gerrits et al. (2012) reported that RS contained 73% and 83% of the metabolizable and net energy, respectively, of enzyme digestible starch in 23 kg pigs. This reflects the well-recognized inefficiency of fermentation as opposed to enzymatic digestion.

Based on the current state of the literature, RS should provide a viable product for use in phase 1 (days 0–7) and 2 starter diets (days 7–21) in ABF production systems. The beneficial effects on gut form and function, and on the microbial population, appear to be encouraging. However, greater research is required to bring clarity to the use of the different categories of RS and how they should be utilized in practical diets to achieve maximum and predictable outcomes. Further research is also required on optimum levels of residual starch to be included in the diet, but at the moment, most research falls between 5% to 10% of the diet.

#### Yeast cultures and yeast cell wall products.

Yeast products have been utilized in pig nutrition for many decades. They are generally divided into three broad categories: live yeast, yeast cell wall products, and products derived from yeast fermentation or culture. The manner in which the products are produced, including temperature, incubation period, nutrient source, and the specific yeast utilized can all potentially impact the benefits which might accrue from their use (Burdick Sanchez et al., 2021). Yeast culture is a dried fermented product that may also contain small amounts of live yeast cells—generally Saccharomyces cerevisiae—as well as metabolic intermediate and endpoint products of fermentation such as enzymes, vitamins, and oligosaccharides. In contrast, yeast cell wall products typically are rich sources of mannan oligosaccharides (30% to 70%) in the form of mannosylated proteins and (1,3)(1,6)-β-D-glucans (35% to 60%), with smaller amounts of chitin (1% to 8%; Spring et al., 2015). They are by-products of the extraction of yeast, which results from lysis or hydrolysis of the cell walls to release the soluble contents, also known as yeast extract. Yeast cell walls produced from autolysis contain about double the quantity of mannan oligosaccharide as those produced by hydrolysis. The residual cell walls may be sold as-is for livestock consumption, but increasingly are dried to facilitate a longer shelf life and lower transportation costs, thus adding value to what was previously a by-product. It is clear that yeast-based products represent a diverse and heterogenous category of feed additive, and that understanding their development and manufacture are essential to their effective use in pig diets.

Yeast products have been shown to function in a number of ways to achieve benefits in the health and performance of the young, newly weaned pig. Looking at the impact of yeast and yeast products in the diet, the benefits would likely be enhanced under ABF production situations (Mayorga et al., 2021). For example, yeast and related products have been shown to enhance intestinal barrier function, normalize intestinal epithelial architecture, reduce diarrhea, stimulate innate immunity, and improve growth performance in *e. coli*-challenged pigs, in some case to an extent similar to that of pigs receiving antibiotics (Che et al., 2017). The benefit of yeast products in pig diets raised under heat-stressed conditions is inconsistent (Mayorga et al., 2021).

Yeast products are typically utilized in nursery diets with the objective of improving the rate and efficiency of growth, as well as enhancing overall animal health. They have sometimes been included in sow diets in an attempt to improve litter performance (Taylor-Pickard et al., 2017). In an extensive summary of peer-reviewed publications on the topic of growth-promoting antibiotic alternatives and covering the period from 1990 to 2016, Schweer et al. (2017) reported that 24% of studies with yeast products reported an improvement in ADG, 12% in ADFI and 11% in feed efficiency. Thus, the improvement in growth performance expected from yeast supplementation is not always reliably observed (Chance et al., 2021).

### Feed Processing

Feed, and the ingredients included therein, are processed for a variety of reasons, but fundamentally it comes down to presenting the pig with a consistent diet that meets all of its needs for maintenance, growth, lactation, and pregnancy (Stark, 2012). More recently, this definition can be expanded to include enhancing animal health, especially gut health, and maintaining biosecurity. A detailed treatise on feed processing is beyond the scope of this review, but important issues as they relate to ABF products will be discussed. As our understanding of ABF production evolves, it is increasingly understood that paying attention to the details of feed processing can contribute to the success of ABF production; failure to do so will have the opposite effect.

Stark (2012) provided a very detailed overview of the role of feed processing in successful pork production. He proposed that, first and foremost, all individuals or teams contributing in some way to the feed production system—ingredient purchasers, feed mill operators, truckers, nutritionists, and barn production specialists—must be working together to achieve a common goal, which is a low cost of production leading to profitable raising of hogs concurrent with the maintenance of animal welfare and the achievement of a high quality, final pork product—all within the context of environmental sustainability. Feed processing also impacts many aspects of the health as well as the nutrition of the pig. It will contribute to maximizing feed intake, reducing waste, avoiding noxious toxins, and achieving such a degree of internal uniformity that nutrient intake by individual pigs will be constant from day to day, even when daily feed intake is low, such as occurs immediately after weaning (Patience, 2017). Properly processed feed will maximize energy and nutrient intake and utilization, contribute to a healthy gut, minimize mortality, enhance feed hygiene and maximize the effectiveness of feed additives (Millet et al., 2012; Kiarie and Mills, 2019).

Feed processing begins with purchasing and delivery, which has as its objective, achieving a consistent supply of ingredients that meet nutritional and biosafety specifications as defined by the nutritionist, are free of noxious toxins and contain antinutritional factors and mycotoxins within acceptable tolerances. This, of course, must be achieved at the lowest possible cost.

The process then continues to the actual manufacture of the feed, which starts with particle size reduction as needed. Decreasing feed particle size results in increased digestibility of dry matter, protein, starch, and energy, as well as improved feed efficiency (Vukmirović et al., 2017; Acosta et al., 2019; 2020). It also enhances feed movement and transportation inside and outside the feed mill and barn, and supports the production of a relatively uniform feed mixture, even though the original ingredients may vary widely in particle size and bulk density. Since it is closely related to GI health, management of grinding assumes greater importance in ABF production. If the particle size is too small, it could lead to gastrointestinal disturbance such as ulcers or gastric torsion and problems associated with feed delivery which in turn can result in out-of-feed events. Limited data suggest that larger mean particle sizes will create healthier conditions in the stomach of the pig, notably lower pH and greater microbial diversity. Unfortunately, the greater particle size feed will also reduce diet digestibility (Vukmirović et al., 2017). Selecting the correct particle size to balance GI health and overall animal performance will be influenced by the composition of the diet and the susceptibility of the herd to pathogenic *E. coli*. Typically, larger particle size is preferred when *E. coli* is an issue, as this helps to increase the abrasiveness of the diet, which in turn impairs adhesion of the pathogen to the epithelial cells in the gut. Larger particle sizes of grains also tend to be gentler on the gastrointestinal tract; an example is fewer problems with ulcers as particle size increases.

While both hammer mills and roller mills are options for grinding, the best equipment and the optimum particle size, in terms of diet digestibility, will depend on both the ingredient being ground and the grinder being used. In other words, the optimum particle size for wheat may differ from that of corn, and the most desirable particle size may differ between a hammer mill and a roller mill (Stark, 2012; Acosta et al., 2019; 2020). Also, feed mill capacity will consequently be reduced when grains are ground more finely, since this will reduce milling throughout. Nonetheless, in the case of corn, the recommended mean particle size is between 500 and 700 μm; lower particle sizes are possible, but the feed would need to be pelleted to avoid problems with flowability (Healy et al., 1994).

Excessive variability in particle size within the feed can be as serious a problem as incorrect average particle size (Patience et al., 2011). Indeed, when it comes to gut health, the mean particle size of a feed may be less important than the distribution of particle sizes within the feed. For example, Cappai et al. (2013) reported that diets are at a higher risk of causing ulcers if more than 36% of the particles are less than than 400 μm in size, and that lower risk could be achieved by lowering this proportion to less than 29%.

Following mixing, the blend may then be delivered to the farm, or may be further processed by some form of hydrothermal treatment, such as pelleting or possibly even extrusion. Pelleting has the advantage of ensuring the maintenance of feed uniformity and thus is often applied to stages of production when feed intake is low. It may also be applied when the cost of pelleting is less than the financial benefit of improved digestibility and, at least theoretically, lower wastage (Behnke, 1996). For this reason, finishing diets are pelleted in some parts of the world where feed costs are generally higher. Hydrothermal processing may also improve the hygienic status of the feed, although neither the temperature nor the duration of heat application is sufficient to kill all organisms. Like particle size, heat processing must be carefully managed because temperatures that are too high or too low can result in negative consequences. For example, excessive temperatures which might be achieved during pelleting may reduce nutrient digestibility, damage heat-labile vitamins and enzymes and other feed additives, and potentially lead to an increased incidence of gastric ulcers (Behnke, 1996). This is one more example of how feed processing must be carefully controlled to achieve maximum benefit to cost and performance but with minimum risk to the health of the pig.

## MANAGEMENT STRATEGIES TO IMPROVE ABF PRODUCTION

Proper diet formulation is critical to the success of ABF pork production, but no amount of manipulation of feed composition or use of feed additives will fully achieve the pig’s growth potential. It is only when the management of the pig, combined with careful diet formulation, that true success will be achieved. This section of the manuscript will identify those aspects of barn and pig management that have been shown to be critical, if not essential to success in ABF production.

### Adoption of Robust Genetics

Selection of pigs with reduced susceptibility to stress and disease is by no means a new concept. More than 40 yr ago, the discovery of the halothane gene allowed for selection of breeding stock that would produce offspring free of this specific form of stress susceptibility (Christian, 1972). Selection of pigs resistant to F18^+^*Escherichia coli* infections is possible due to the failure of some individuals to express F18R, the receptor necessary for adherence of the pathogen to the gut epithelium (Meijerink et al., 2000), which can be easily identified based on a genetic test. Similarly, a genetic marker that is associated with improved host response to PRRSv has been identified (Boddicker et al., 2012), but it only confers partial resistance.

More recently, clustered regularly interspaced short palindromic repeats (**CRISPR**)/Cas9 gene-editing technology has offered the prospect of establishing lines of pigs that are resistant to the PRRSv (Whitworth and Prather, 2017) as well as other diseases of importance to the pig industry. It is important to note that PRRSv, due to its immunomodulatory impact, causes infected pigs to be more susceptible to many other diseases. Control of PRRSv makes ABF a much less risky proposition; indeed, some producers who are experienced with ABF production believe that freedom from PRRSv is an essential criterion for success. Most critically, this technology has the potential to greatly enhance the welfare of pigs. While there are obviously ethical and societal issues that need to be addressed, the benefits of adopting CRISPR technology would be a tremendous development in pork production.

Rather than selecting for resistance or reduced susceptibility to specific diseases or stressors, selection for resilience has been identified as a more desirable target for genetic improvement (Knap and Doeschl-Wilson, 2020). Resilience is defined as ‘the capacity of animals to respond to short-term perturbations of their environment and return rapidly to their pre-challenge status (Colditz and Hine, 2016). These perturbations include environmental stress and disease. Unfortunately, selection of animals with improved resilience is proving to be difficult. There are challenges in identifying the genetic target, or even quantifying the desirable trait, at least in some instances (Knap and Doeschl-Wilson, 2020). The development of appropriate biomarkers would be particularly helpful in this regard (Kasper et al., 2020). As a consequence of the complexity of the problem, and the need to first develop proper selection targets, progress has been slow, although advances in automated phenotyping provide new opportunities (Berghof et al., 2019). As the demand for ABF production increases, there will no doubt be greater incentives for geneticists and breeding companies to redouble their efforts on this topic.

### Perinatal and Post-weaning Pig Care

The importance of detailed care of newborn and newly weaned pigs is well accepted (Lawlor et al., 2020); its value is even greater in ABF production, because producers cannot depend on antibiotics to assist the piglet during these highly stressful periods. However, there is surprisingly little literature on the topic. Much of what we know or believe to be true about piglet care has evolved by trial and error on thousands of farms over decades of time, supplemented with some excellent research in key areas.

Care of the neonate is an awesome and complex responsibility. Even before it is born, the fetus suffers challenges to its vitality, including the possibility of intrauterine growth retardation during gestation and hypoxia during farrowing, combined with physiological immaturity at birth (Farmer and Edwards, 2020). It is born with very limited energy reserves, which make it dependent on early and frequent consumption of its mother’s milk; poor thermoregulatory capability further compromises the pig’s survivability (Villanueva-Garcia et al., 2020), a risk factor that is magnified by low and highly variable milk intake among piglets, since metabolism of feed generates critically important heat energy within the body. Further, the fetal placenta prevents the transfer of antibodies from dam to fetuses. Along with an immature immune system, the piglet’s dependence on immunoglobulins from the sow’s colostrum and milk for protection from pathogens cannot be overestimated. Low birthweight, which is more common with the hyper-prolificacy of the modern sow (Beaulieu et al., 2010), compounds the newborn’s tenuous adaption to life outside the womb, the consequences of which are exacerbated by a slow birthing process which may lead to hypoxia (Farmer and Edwards, 2020). These problems are magnified by a large surface area to body mass ratio, which encourages body heat loss. If thermoregulation is not properly managed, it will lead to a potentially fatal cascade of events starting with an energy deficit which in turn leads to hypothermia and thence illness, or at the very least, increased risk of being overlain by the sow due to weakness. Even if the piglet survives, physiological and anatomical damage which may occur after farrowing or weaning could be permanent, resulting in poor performance and susceptibility to disease throughout its life (Moeser et al., 2017).

Success in the care of neonatal pigs therefore begins before birth and is influenced by factors related to intrauterine growth retardation and placental quality (Farmer and Edwards, 2020). Proper feeding of the sow to ensure adequate but not excessive energy and nutrient intake is essential. Increasing feed intake late in gestation, so-called “bump feeding,” remains controversial, but the preponderance of evidence suggests it is not impactful in terms of increasing average piglet birth weight, stillbirths, or other indicators of improved litter size and viability; however, the portion of low BW piglets may be improved (Mallman et al., 2019a,b; Araújo et al., 2020). One more promising technique is to feed the perinatal sow at least three times per day. Based on recent research, the sow may be suffering from a glucose deficit due to the sudden onset of lactation; feeding more frequently may help the sow improve her energy status and positively affect the farrowing process leading to a lower rate of stillbirths (Feyera et al., 2018).

While feed composition and feeding strategies for the sow are important, other aspects of management are equally impactful. Creating a warm, dry, and draft-free environment in the farrowing barn will help the neonate adjust to life outside the womb. This will require creation of a microclimate in the creep area of the farrowing crate, since temperatures considered ideal for the young piglet—estimated by Mount (1959) to be 34 °C—will be excessive for the sow. Herpin et al. (2002) reported that the lower critical temperature of the modern piglet is not much different from the past: 35 °C at 2 h post-farrowing declining to 33 °C at 24 h and 30 °C at 48 h. Assisting each pig to obtain at least 200 mL of colostrum within the first 24 h of life, and preferably 250 mL, is highly beneficial in reducing mortality, especially among low and medium birthweight piglets (Ferrari et al., 2014; Quesnel et al., 2012). Quesnel et al. (2012) reported mortality rate to weaning as low as 7.1% when colostrum intake during the first 24 h post farrowing exceeded 200 mL but as high as 43.4% when colostrum intake was lower. In addition, piglet growth rate and immune status are closely correlated with colostrum intake.

Split suckling litters early in life is sometimes practiced to facilitate more equal distribution of colostrum across all pigs in the litter. This is especially beneficial if the number of piglets exceeds the sow’s number of teats. Once colostrum has been consumed, cross-fostering of piglets and the use of nurse sows may be implemented to optimize weaning weights and reduce pre-weaning mortality. There are a variety of approaches to cross-fostering; the most advantageous will depend on individual circumstances within the litter (Vande Pol et al., 2021). The health status of a herd must be considered before cross-fostering is implemented due to its significant impact on the spread of pathogens within the farm, including the PRRSv (McCaw, 2000).

Batch farrowing has grown in popularity as a means of reducing the age variation within a population of pigs, and thus enhancing the ability of barn staff to control pathogen exposure. Variation in age makes this much more difficult (Dewey et al., 2006). Batch farrowing also helps the farm follow another key feature of success, namely no co-mingling of pigs of different ages within a given airspace, or ideally, a given site; this is frequently referred to as all-in-all-out production. It also provides greater flexibility in cross-fostering, especially on farms with smaller sow herds. All-in-all-out operation is considered essential for success in ABF production.

Management of pigs post weaning represents another specialized challenge, though different from that of managing the neonate, but with many common themes. Success in this instance requires creation of a physical environment which is warm, dry, and draft free. It also requires provision of an initial post-weaning diet which is highly digestible and palatable, containing concentrated and highly digestible sources of energy and nutrients. Maximizing feed intake is critically important in the newly weaned pig; not only is it valuable in its own right, but creating an environment that achieves this objective provides benefit to the pig in other ways as well. For example, health challenges are one of the major deterrents to enhanced feed intake, but steps taken to prevent illness, or to manage it effectively, provide benefits well beyond improved feed intake.

Early feed intake post weaning not only improves growth rate, but it also helps to protect the newly weaned pig from hypothermia, strengthens the immune system, and contributes to the maintenance of a healthy gastrointestinal tract architecture. In other words, the benefits of successful introduction to feed post-weaning are the same as those achieved in the care of the new-born piglet. Strategies to enhance feed intake will be presented later in this section.

### Minimize Social and Other Stressors

Appleby (1996) suggested that animal well-being requires meeting the physical, environmental, nutritional, behavioral, and social needs of the animal or groups of animals. Examples of social stress in the life of the pig might be related to group size, floor space, feeder space, and mixing (Wellock et al., 2003). It is, of course, well known that weaning represents a very stressful event in the life of the pig; indeed, it may be the most stressful event. A high level of individual pig management is required to minimize the impact of these stresses, not just in the immediate period after weaning, but throughout the pig’s life. For example, Pohl et al. (2017) reported that early life adversity increased susceptibility to GI disorders, including diarrhea, increased mast cell activity, and impaired intestinal permeability, especially in the ileum. Notably, such pathologies persisted much later in life. Moeser et al. (2017) provided particular focus on the issue of gut permeability and the long-term consequences of its disturbance early in the pig’s life. In conventional production systems, antibiotics can be used to assist the pig in dealing with these disruptions in gastrointestinal function. However, in ABF production, this is not possible. Consequently, reducing the stress of weaning to the lowest possible level becomes critically important. The following sections will address management practices which can be employed to reduce stress on the pig—not just at weaning, but throughout the pig’s life.

#### Older weaning age.

 Weaning pigs at 3 wk of age or less became popular in the latter decades of the 20^th^ century as a means of reducing pathogen transmission from the sow to her offspring, thus improving piglet health and performance. (Robert et al., 1999; Beaulieu et al., 2006). It was frequently associated with multi-site production, meaning that weaned pigs were moved to a facility that was separate from the sow unit, to further limit pathogen transmission. Patience et al. (2000) reported that pigs weaned at 12 d of age, but removed to a location different from that of the sow herd, were heavier at 56 d of age compared with pigs weaned at either 12 or 21 d of age but retained on the same site as the sows. In the past, the age at which weaning occurred depended on the pathogen of interest, and ranged from 10 to 21 d of age. Weaning at less than 21 d of age would facilitate elimination of such pathogens as Pseudorabies, *Actinobacillus pleuropneumoniae* (APP) and transmissible gastroenteritis (TGE; Harris, 1988). Weaning at less than 14 d would support elimination of *Haemophilus parasuis* (HPS), while weaning at less than 10 d of age would support elimination of *Mycoplasma hyopneumoniae* and *Bordetella bronchiseptica*.

Over time, problems inherent with weaning at less than 21 d of age became increasingly apparent. They include a greater incidence of social vices (Metz and Gonyou, 1990), and the consequences of a less mature GI system (Moeser et al., 2007) and immune system (Blecha et al., 1983), leading to disruptions in health and performance at weaning. As one example, Smith et al. (2010) reported that pigs weaned at 15 to 21 d of age experienced sustained intestinal barrier function impairment, as compared with pigs weaned at 23 to 28 d of age.

Postma et al. (2016) reported average ages at weaning of 35 d in Sweden and 24 d in Germany, Belgium and France, based on a survey of 232 farms. A comparison of 262 Spanish and 365 US hog farms indicated average weaning ages of 25 and 21 d, respectively (PigChamp, 2020). The same publication reported an average weaning age of 21 d in Canada (38 farms) and 26 d in South Africa (54 farms).

There is now a trend towards weaning pigs at an older age in North America; newer farrowing units are being sized to accommodate weaning at 22–24 d of age. Main et al. (2004) reported that as weaning age increased, mortality decreased, day 42 bodyweight increased and weight at harvest reflected greater weight per day of age. In that study, the oldest weaning age was 21 d. Boyd et al. (2019) reported that nursery mortality and total mortality to harvest declined as weaning age increased from 18 to 24 d of age, and that the benefit was greater if sow flows were positive for PPRSv and PEDv. McLamb et al. (2013) reported that small increases in weaning age, from 16 to 18 to 20 d of age, greatly enhanced the ability of pigs to respond to an ETEC challenge and reduce its physiological and immunological impact.

Overall, older weaning typically resulted in heavier weights leaving the nursery (Main et al., 2004; Leliveld et al., 2013) or fewer total days to achieve market weight (Main et al., 2004; Faccin et al., 2020a). Older weaning ages produced other benefits, such as reduced incidence of social vices, including belly nosing. Benefits also included lower fecal *E. coli* counts, lower morbidity and mortality and reduced need for antibiotic intervention (Widowski et al., 2003; Leliveld et al., 2013; Faccin et al., 2020c). Based on the available data, the trend towards older weaning ages, common in Europe, is likely to accelerate in North America in herds seeking to implement ABF production.

#### Create a high-quality barn environment, including air quality.

 Building ventilation is far more important to the achievement of success with ABF than many people realize, because it speaks directly to the amount of physiological stress being experienced by the pig. It starts with a properly designed and installed ventilation system, preferably involving input from an engineer with formal training and relevant field experience. This system will include fans, air inlets, and heaters, as well as an integrated electronic controller to ensure all components are operating in synchrony with each other; sometimes, cooling equipment may also be included in the design. It continues with properly trained and motivated barn ownership and staff, operating the system as intended when it was designed. Over time, it will require frequent maintenance to ensure that air exchange and distribution, for example, has not declined due to damage or wear on fans, inlets, and other system components.

The building design needs to consider thermal comfort in terms of more than just air temperature. For example, poorly insulated walls will increase heat loss from pigs during cold weather due to radiation; it may also lead to building condensation. Cold floors will also cause chilling, due to conductive heat losses, which can make pigs more susceptible to diarrhea, especially recently weaned pigs. An improperly designed or operated ventilation system can be a significant contributor to respiratory disease in pigs. Excessive airspeed over the pigs will increase heat loss due to convection; this will have a negative effect on the pigs when air temperature is at or close to the lower critical temperature of the pig, but could have a positive effect if the air temperature in the barn is above the upper critical temperature. If the air temperature is close to skin temperature of the pigs, such air movement will have much less impact. To obtain the best and most representative measurement of air temperature in a barn, thermometers or probes should be placed centrally, away from exterior walls, as close to pig level as possible but out of their reach, and distant to heaters and air inlets that could result in incorrect readings.

Air inlets need to be sized to match the capacity of the ventilation fans. And air inlets should also be adjusted to generate air movement into the barn in the range of 3.5 to 5 m per second. Fans and associated shutters will need to be cleaned frequently, since their output will be affected by accumulated dust or damage—by as much as 40%. Air inlets probably need to be reset before each barn fill; clamps can slide and cords can stretch. Something as simple as variations in the openings among air inlets has been found to affect the incidence of social vices such as tail biting.

When the outdoor temperature is less than the target interior temperature of the barn, the basic objectives of a ventilation system are to prevent the build-up of noxious gases such as ammonia and hydrogen sulfide, to manage humidity within a desirable range of 40 to 65%, to manage air speed at the level of the pigs and to maintain the air temperature in the barn within the pigs’ thermoneutral zone as well as avoid wide fluctuations of temperature. To control humidity in the heating season, a minimum exhaust capacity of ~2 cfm per head is required for pigs in a nursery and ~10 cfm per head for pigs in growout.

To reduce heat stress on pigs, evaporative coolers, also called cooling pads, may be installed in hog barns. Pigs are particularly susceptible to heat stress (Patience et al., 2005), so cooling equipment makes sense. The decision of whether or not to include them in the building design process will involve a cost-benefit analysis. They are most frequently installed in sow barns and much less so in grow-finish facilities in temperate regions. However, when ambient humidity levels exceed 80%, such equipment will have limited value—lowering temperature by perhaps no more than 2–3 °C. Under more ideal conditions, a much larger temperature reduction—in the range of 10 °C or greater—is possible (Stinn and Xin, 2014). Other options for cooling pigs include sprinkler systems (common in grow-finish situations), foggers and geothermal installations (Jacobsen, 2012a).

A successful ventilation system will theoretically maintain barn temperature within the pig’s thermoneutral zone and have a positive contribution to the health and welfare of pigs. Using the example of a finishing pig weighing 70 to 125 kg, this will be 14 °C to 21 °C. However, the temperature that supports maximum growth rate will be 16 °C; feed efficiency will be optimized at 20 °C (Jacobsen, 2012b). Unless cooling systems are installed in the barn, it will not be possible to maintain pigs within their thermal comfort zone in hot weather, resulting in physiological (heat) stress and reduced performance. When the air temperature outside the barn is above skin temperature of the pig, tunnel ventilation or stir/mixing fans will be helpful in reducing the degree of heat stress. As a rule, during hot weather, the lowest temperature that can be achieved in a conventionally ventilated barn is approximately 2 °C above ambient.

Dust and noxious gases may also contribute to pig stress. For example, Wathes et al. (2004) reported that a combination of airborne dust at 5 to10 mg m^-3^, compared with 0 or 2.5 mg m^-3^ and ammonia concentrations as low as 10 ppm, resulted in reduced growth in weanling pigs while feed conversion appeared to be unaffected. By the same token, dust alone, or ammonia alone, did not appear to impair growth at all. Done et al. (2005) looked at the clinical and pathological impact on these same pigs and concluded there was no impact (Done et al., 2005). These results differed from Michiels et al. (2015) who reported that particulate matter in the air was linked to increased lesions associated with pneumonia and pleurisy. Across the literature, it appears that a combination of dust and ammonia can be problematic, but perhaps not to the extent that many people expect. An important question that needs to be answered is the impact of dust and/or ammonia in pigs that are already suffering from respiratory disease.

#### Appropriate group size and floor space.

Group size and the provision of adequate floor space are two quite distinct topics. However, it is best to consider them jointly since a considerable portion of the available research addresses the two concurrently. Indeed, much of the literature confounds floor space with group size, and vice versa (Smith et al., 2004; Street and Gonyou, 2008; Flohr et al., 2016). Information upon which specific and independent recommendations can be made on either optimum floor space allowances or on the influence of group size on performance and well-being of swine is quite limited, but increasing in more recent research (Schmolke et al., 2003; Wastell et al., 2018; Laskoski et al., 2019).

Schmolke et al. (2003) concluded that neither animal performance nor health are affected by group sizes ranging from 10 to 80 pigs per pen, provided that equal resources (feed and water access) and floor space are provided. Wolter et al. (2001) reported similar growth performance with 25, 50, or 100 pigs per pen in a wean-to-finish barn; however, removals were greater with the smaller group size. Spoolder et al. (1999) found no difference in performance in pens housing 20, 40, or 80 pigs, although skin lesions—indicative of aggressive behavior—increased with group size, the opposite outcome compared with Wolter et al. (2001). Previously, Wolter et al. (2000) reported poorer performance with 100 pigs per pen compared with 20 pigs per pen. However, this trial lasted only 9 wk; since pig performance was slower in large group sizes during the first half of the study reported by Wolter et al (2001), the two studies generated very similar outcomes when considered over the total growout period. More recently, Meyer-Hamme et al. (2016) compared group sizes from <15 to >30 and found no impact on indicators of animal well-being; growth performance was not measured. Under the conditions of a commercial research facility, Olsen et al. (2018) compared 11 or 31 pigs per pen and reported that pigs in the smaller groups grew faster and more efficiently; in this study, floor space and feeder access per pig were equalized across treatment.

Defining floor space allowance according to an allometric equation was first proposed by Petherick and Baxter (1981), who suggested that an equation in the form kBW^0.667^ where BW is the maximum mean body weight of a group of pigs and k is a constant can be applied across many livestock species. Empirical data can be used to provide an estimate for k and are available in sufficient quantity to provide at least an estimate for use in building design (Gonyou et al., 2006). Based on the analysis of a number of datasets, they recommended a k value for grow-finish production on fully slatted floors between 0.0317 and 0.0348; they further estimated that for every 0.001 reduction in k, growth rate declined by an average of 1% (range from 0.56 to 1.41%). The application of the allometric approach has more or less been widely accepted as a means of determining adequate floor space for pigs from weaning to market, and appears to be supported by most data.

Disagreement remains on the optimum value of k. DeDecker et al. (2005) compared three group sizes of 22, 27, and 32 pigs per pen, all with the same total pen floor space. Performance up to 35 kg was unaffected by treatment, and k was no lower than 0.051. However, at the end of the second phase of the study, when control pigs (*k* = 0.039) reached 91 kg, growth rate was impaired by *k* values of 0.032 and 0.027. At the end of the experiment, when control pigs (*k* = 0.032) reached 122 kg, growth rate declined with k values of 0.026 and 0.022. Based on the results from phase 2, the control pigs may have been crowded to the point of reducing growth rate. In this study, it is not possible to distinguish the effect of group size from floor space; however, these data suggest that a *k* value above 0.032 is required. Anil et al. (2007) evaluated four space allowances with 19 pigs per pen and concluded that *k* values of 0.034 and 0.037 were acceptable from an animal performance and animal wellbeing perspective.

Thomas et al. (2017) compared *k* values of 0.032, 0.028, and 0.025 for pigs marketed at about 133 kg and housed in pens of 9 pigs each. Since the highest k value was 0.032, it was not possible to determine if a greater value would have been advantageous, but overall, this study supported previous reports and particularly Gonyou et al (2006). When k values fell below 0.032, growth rate declined. Reductions of the k value by 0.004 and 0.007 reduced ADG by 3% and 9%, respectively, fairly close to the expected decline of 1% for every 0.001 reduction in the *k* value.

Jensen et al. (2012) compared three values of *k* (0.033, 0.036, and 0.039) on two commercial farms involving more than 7,000 animals housed in pens of 14 pigs each; based on growth performance and pen hygiene, there was only a slight advantage in growth rate due to increasing floor space (*P* = 0.07; 1.006, 1.009, and 1,011 g/d) and also a slight advantage in gross margin per pig. The authors concluded that a k value greater than 0.033 could not be justified. Over-crowding has been identified as a stressor that can impact pig performance and other indicators of well-being. Unpublished data have suggested that ABF production will require greater floor space allowances than are currently employed by the commercial pig industry in many parts of the world. The allometric approach to defining floor space requirements may be very useful in defining effective and successful floor space allowances. Group size appears to be less of an issue, based on the preponderance of data described above.

#### Minimized body weight variation.

Jobling (1995) has described what might be referred to as a “social behavior model” for variability, albeit in another species (Arctic Char). He describes how competition for resources will lead to stress among animals in a group, lowering performance and increasing the variability of growth. According to this approach, but applied to swine, any resource which can lead to competitive behavior, such as feed access and water access, will confer an advantage to dominant animals in the group, while concurrently impeding performance of animals less dominant in the social hierarchy. This would lead to increases in variability. This model can serve as a useful tool to anticipate factors that may or may not increase variability in body weight within a group of pigs. As an example, Spoolder et al. (1999) reported that 20 pigs per feeder space resulted in more aggressive behavior while eating compared to 10 pigs per feeder space. If the resource does not offer a potential advantage to dominant animals, it may lower average group growth performance, but it will not impact variability. The exception to this rule is health; dominant animals can be equally stricken by illness as compared to less dominant animals, but disease certainly increases variation in growth performance (Cornelison et al., 2018). However, illness probably increases variation not due to some inherent advantage of dominant pigs, but rather the inequality of the impact of illness within a group of pigs.

The question of sorting pigs into pens by weight, either at weaning or upon entry into the finishing barn, in order to reduce variability in body weight at marketing, arises from time to time. The data are quite clear that reducing variability of bodyweight within the pen does not reduce the variability in bodyweight at the time of harvest; indeed, it may even slow growth rate compared to pigs being randomly assigned to pens (Gonyou, 1998; O’Quinn et al., 2001; Faccin et al., 2020b; Hastad et al., 2020). It has also been shown that sorting pigs by body weight did not reduce aggressive interactions post weaning. There is one exception to this since there is a need to select the smaller or poor performing pigs from a larger group for housing in separate pens so they can receive more specific attention, including a warmer microclimate and perhaps a different feed budget, compared to the rest of the barn. Indeed, sorting smaller pigs from the larger population has been shown to increase their feed intake postweaning (Bruininx et al., 2001). Increasing dietary energy content through the addition of fat may be more beneficial when applied to the smallest 50% of a barn rather than the whole barn (Hastad et al., 2020).

#### Maximize feed intake.

Maximizing feed intake is one of the most critical factors contributing to success in swine management. This is especially true in ABF production systems. It maximizes growth rate in the nursery and finishing barns, and it maximize milk production in the farrowing barn (Nyachoti et al., 2004). The only exception, of course, is the gestating sow which is normally limit fed. One of the most difficult times to achieve maximum feed intake occurs in the first week post weaning (Wensley et al., 2021a, b). Bruininx et al. (2001) reported that only about 50% of pigs have eaten within 4 h of weaning, with a quarter failing to eat during the first 24 h after weaning. Shockingly, 10% of pigs still have not eaten 30 h after placement in the nursery and 5% have consumed no feed within the first 48 h post weaning. These data underscore the need to encourage eating in the nursery in every way possible as soon as possible; particular attention must be paid to piglets that are showing early signs of inappetence after weaning.

The newly weaned pig thus faces low energy and nutrient intake resulting from unfamiliar environmental, social, and nutrition experiences. Impaired feed intake potentially results in damage to the GI tract (Dong and Pluske, 2007), which in turn leads to reductions in the efficiency of energy and nutrient absorption; it may also lead to intestinal “leakiness“ resulting in greater internal exposure to pathogens and immunostimulatory fragments (Spreeuwenberg et al., 2001). Nothing is more important at the time of weaning than getting pigs eating and drinking as quickly as possible; pigs that are eating well are less affected by chilling, are more resistant to pathogens, compete more effectively for limited resources, and generally grow faster and reach market weight sooner. Therefore, feed delivery and supply are critical to the pig’s survival, well-being, and growth performance. The most certain predictor of post-weaning feed intake is weaning age. Faccin et al. (2020a) and Leliveld et al. (2013) represent most studies on the subject, showing that as weaning age increases, feed intake rises.

Creep feeding is frequently justified by expected increases in weaning weight; however, the more important justification could be greater intake of feed post-weaning. A barrier for many North American farms is the very limited intake of creep by pigs weaned at less than 25 d of age. Bruininx et al. (2002), studying pigs weaned at 28 d of age, reported that pigs that had consumed creep feed prior to weaning initiated feed intake after weaning much more quickly than pigs that had not consumed creep feed. Ninety percent of pigs receiving creep feed had consumed feed within 10 h of weaning, while those who had not consumed creep feed required more than 24 h to achieve the same participation in feed intake. Sulabo et al. (2010b), studying pigs weaned at 20 d of age, also reported that pigs consuming creep feed ate more and grew faster after weaning, although in their study, the effect lasted only 7 to 14 d. A review of the broader literature reveals equivocal outcomes regarding both benefits in weaning weight and in post-weaning performance.

There are many possible reasons for these differing phenotypes arising from the use of creep feed. The first is the age of weaning. Tokach et al. (2020) reported that creep feed intake rises by about 50% when weaning age increases from 20 to 24 d and increases an equal amount again when weaning age extends to 27 d of age. Notably, weaning age varies widely in studies on post-weaning feed intake reported in the literature. The second reason for these different phenotypes is the relationship between birth weight and creep intake. Pajor et al. (1991) noted that pigs with a high birth weight tended to eat more creep feed, and initiated creep feed consumption sooner. Higher birthweight pigs also tend to grow faster post-weaning, independent of creep feed consumption (Beaulieu et al., 2010). Therefore, one has to be careful to not assign improvements in post weaning growth rate solely to creep feed when it could in fact be due to birth weight effects. There are many other possible variables at play, including genetics, litter size, herd health status, method of delivering creep feed, and the composition of the creep feed. Sulabo et al. (2010a, b) provide solid data supporting the last two items in this list. The most consistent response to creep feeding, but by no means an assured response, is improved feed intake post weaning.

Adequate feeder capacity is critically important in the delivery of feed to the newly weaned pig. Feeder capacity, in turn, can be defined in two ways. The first is based on linear inches of feeder width; using this method, nursery pigs are suggested to each have 2.5 cm and grow-finish pigs 5 cm of feeder width available. Due to their support of more rapid eating behavior, wet/dry feeders require only 3 cm/grow-finish pig (PIC, 2019). The concept of using feeder width recognizes that pigs require a minimum length of feeder to simply accommodate the basic width of the pig as measured at the shoulder. Typically, the recommended feeder length represents the shoulder width of the pig plus 10%. However, pig width has increased due to genetic changes in the overall dimensions of the pig’s body, which appears to be getting wider and shorter, per unit of body weight; Condotta et al. (2018) reported that, for example, a 100 kg pig in 1963 was 30.5 cm wide at the shoulder but that same pig in 2017 was 34.6 cm wide.

Length of the feeder defines how many pigs can physically fit at the feeder to eat at one time, assuming that the full width of the feeder is available for eating, and not divided into arbitrary feeding spaces. Another approach to defining feeder capacity is to define the ratio of pigs per feeder space; in other words, how many pigs in the pen are assigned to each available feeder space. Two pieces of information are required to convert feeder spaces to feeder capacity: length of time each pig spends eating each day and the reasonable maximum capacity of the feeder, expressed as a percentage of the day, during which the feeder can be occupied without impairing feed intake. Not surprisingly, there is considerable variation in the time pigs spent eating each day. Smith et al. (2004) reported that pigs spent 120 min eating per day 3 to 6 d after weaning (BW ~7 kg) and 82 min per day 39 to 42 d after weaning (BW ~ 29 kg). Hyun et al. (1998) reported that between 36 and 56 kg BW, pigs spent 125 min eating per day; they also reported that an average occupancy of 70% did not impair feed intake. In a subsequent study, Hyun et al. (2002) reported that pigs spent about 100 min eating per day; in this instance, feeder occupancy averaged 43%, and even when occupancy rose to 75%, feed intake did not appear to be impaired. These last two studies utilized group sizes of up to 12 pigs per pen, which is well below commercial practice in most countries; it is not known if larger group sizes would change these conclusions.

Li et al. (2017) studied this topic more intensively in growing pigs from 21 to 60 kg and finishing pigs from 60 to 93 kg BW and reported that growing and finishing pigs spent about 105 min eating per day when offered a mash diet from a dry feeder. Eating time declined to 73 min/d for growing pigs and 64 min/d for finishing pigs when offered the same diet from a wet/dry feeder. Pelleted diets required about 77 min of eating time/d whether the feeder was dry or wet/dry; finishing pigs spent 65 min/d under the same diet and feeder combinations. Overall, they concluded that whenever theoretical feeder occupancy exceeded 80%, either eating time or growth performance suffered. Feeder occupancy was calculated as total eating time (minutes per pig per day) times the number of pigs in the pen divided by the number of true feeding spaces divided by 1,440 total minutes in a day. True feeding spaces refer to the actual number of pigs that can eat from the feeder at any one time, which will be impacted by both the length of the feeder and the number of feeder spaces provided by the feeder, whichever is less.

There are very few studies investigating feeder space requirements under commercial conditions. One such study (Weber et al., 2015) utilized a commercial-scale research facility with 31 pigs per pen. The diets contained 30% or 60% corn DDGS, which was quite high in fiber by North American standards; diets were offered as a mash via dry feeders. The feeders, were modified to provide 5, 6, or 7 feeding spaces (25.4 cm of width per feeder space), but assuming the shoulder width of the pigs was 34.5 cm, and allowing for 10% extra space for movement, they actually offered only 3.1, 3.7, and 4.3 feeding spaces per pig. This is an excellent example of the number of holes in the feeder falling short of the number of spaces usable by the pigs. Based on daily feed intake, which was similar across the three feeder-space treatments, access to feed was not an issue, even when pigs were approaching harvest weight at 122 kg. Assuming the pigs were spending 105 min eating/d, maximum feeder occupancy never exceeded 73%. Given that feed intake was not impaired by any of the feeder space treatments, the threshold of 80% as suggested by Li et al. (2017) was supported.

Another recent study (Laskoski et al., 2019) conducted under commercial conditions provides further insight into the issue of feeder requirements, and interestingly, also reveals the challenge of determining the number of feeder spaces per pig. The study involved 630 pigs assigned to pens of 15, 20, 25, or 30 pigs each and utilized feeders that were 64 cm wide with 4 feeding holes each. The trial was conducted from weaning until the pigs reached approximately 21 kg. During the first 14 d of the trial, daily feed intake declined in a linear fashion as the number of pigs increased, co-incident with the increase in the number of pigs per feeder space. Throughout the remainder of the trial, there was no effect of treatment on daily feed intake; during two periods, rate of gain was reduced by treatment, but at the end of the trial, BW were not different across treatments. Because pigs per feeder space was confounded by number of pigs per pen, it is not possible to discern if the observed effects were due to feeder capacity or group size, or a combination of the two. Further evaluation of the study perhaps can provide some insight. While the study was designed to compare 3.75 to 7.50 pigs per eating space, the width of the feeder provided at most 2.7 feeding spaces, using the shoulder width data of Condotta et al. (2018) for 20 kg pigs and assuming an allowance of 10% beyond pig shoulder width, as suggested by Brumm (2012). Assuming the pigs spent 120 min eating per day (Smith et al., 2004), the occupancy rate of the feeders ranged from 46 to 91%, with 15 and 30 pigs per pen, respectively. Thus, the occupancy appeared to exceed standards suggesting that the decline in feed intake during the first week could have been at least partly due to feeder capacity and not group size.

Due to the importance of early adjustment to weaning with particular emphasis on feed intake, many producers who wean pigs at less than 25 d of age pay a great deal of attention of this matter. They typically use feed boards for the first week after weaning, to make feed more readily available to the pigs; they may gruel feed—mixing feed with water before presentation to the pig—especially for pigs that show compromised behavior associated with low feed intake. Ideally, one room in the nursery is filled in a single day, so people in the barn can establish one common routine for all pigs and all pigs enter a “clean” barn and air space. This does not mean that pens set aside for small or compromised pigs are not treated differently than their peers; rather, it means that there is only one age of entry into the nursery so there is only one peer group present. Mixing different arrival dates in a single room makes it much more difficult to establish a firm and consistent routine for pig care. If the nursery is not sized to match the size of the breeding herd, batch farrowing is often employed to achieve larger group sizes of weaned pig and thus the ability to fill a larger nursery room with the existing pig source in a single day (Brown, 2006).

Barn temperature is very important, as it relates not just to the comfort of the pig but also to its appetite. When pigs first enter the nursery, it must be very warm, dry, and free of drafts. Furthermore, as stated previously, floors, walls, and ceilings must also be warm at this time. Chilling simply cannot be tolerated during this highly stressful period in the pig’s life. Temperature control in the nursery is absolutely critical. If it is too low, piglets are chilled, but if it is too high, feed intake will be depressed. When the overall room temperature is lowered to encourage maximal feed intake, supplemental heat, using brooders or other thermal source, should be provided to the pens housing compromised pigs so they are not chilled. It is important to note that smaller pigs, and pigs that are not eating well, require a warmer temperature than their peers with better appetites. Chilling due to a lack of metabolic body heat normally provided by digestive processes is a serious problem for this smaller subgroup, which if not corrected, can lead to a vicious circle of poor appetite leading to chilling, which leads to lethargy, which further impairs appetite.

Many other factors can impact feed intake in the newly weaned pig (Dong and Pluske, 2007). They include health status, mixing of litters pre- or post-weaning, mixing different ages of pigs, the physical environment, diet nutrient level, palatability of the diet, forms of diet presentation to the pig, adequacy of the quantity and quality of the water supply, and general stockmanship. There is no recipe for success that applies in all types of production systems, but all of these factors must be addressed in order to maximize feed intake, and thus post-weaning growth rate.

#### Assured water supply and delivery.

An abundant supply of potable water provided in quantities that meet the needs of the pig is a critically important component of any production system seeking to minimize stress and thus be successful in ABF production. Because of the ease and low cost of accessing water for pork production in many pig production regions of the world, this topic frequently does not receive the attention that it deserves, especially given its importance in so many aspects of the pig’s life. Access to water may become more of a concern in the future, as agriculture increasingly finds itself in competition with industrial and urban users of available supplies (Molden, 2007; Rosa et al., 2020).

The quantity of water consumed by the pig is typically related to its feed intake. Most people believe that simply making water available ad libitum is sufficient “management” to ensure the pig’s requirements are being met; this is not altogether correct. Certainly, an inadequate supply of water will lower feed intake, which in itself is a serious source of stress to pigs.

Water fulfills a surprising array of roles in the diet of the pig, contributing to the maintenance of constant body temperature, acid-base balance, and electrolyte balance, assisting in the movement of nutrients and waste to and from cells as well as through the GI tract, and in lubrication. Water is a constituent of numerous chemical reactions, not the least of which are those associated with oxidation and hydrolysis (Patience, 2013).

Even when water is readily available, according to some older literature, there are at least two periods in the pig’s life when “typical” water intake may not meet its physiological needs. The first occurs around the time of farrowing. Research has shown that lethargic sows tend to drink less water during the first 3 d of lactation; this, in turn, is associated with reduced milk production and impaired litter growth (Fraser and Phillips, 1989). The second occurs immediately after weaning, when water intake is initially high and when feed intake is low, declining by day 3 to 5 before it starts to increase in relationship with feed intake (McLeese et al., 1992). In fact, the literature is unclear as to the ability of the newly weaned pig to properly regulate water intake in response to physiological need. For example, there are some data that show increased water consumption in the presence of diarrhea (Patience et al., 2004) while other data do not (Fraser et al., 1993). This remains a quandary in our knowledge about water use by the pig, and its ability at certain stages of life to connect need with consumption.

There is no doubt that pigs can increase water intake when exposed to thermal stress (Patience et al., 2005; Renaudeau et al., 2008). However, water use during periods of heat stress need to be interpreted with care. If nipple drinkers are used, pigs will play with them to spray themselves with water in order to achieve relief from the heat. When this increase in water flow is measured, it is frequently misinterpreted to reflect elevated r intake when it is, in fact, a combination of increases in both intake and waste. Other forms of luxury consumption are a well-known phenomenon in pigs, observed most frequently but not exclusively in adults. Examples include schedule-induced polydipsia and hunger-induced polydipsia (Stephens et al., 1983; Scipioni et al., 2009).

Notwithstanding the issue above that is specific to thermal stress, water intake can be utilized as a management tool to identify health or stress problems within a herd, which may lead to changes in water intake. Therefore, daily water consumption in the barn is increasingly being monitored as a means of achieving an early warning of the onset of illness. In order for this approach to be most effective, patterns of intake throughout the day must be monitored; pigs will adjust their diurnal pattern of water intake in response to a stress—such as inadequate nipple drinker operation. Therefore, monitoring average 24-h consumption could miss some of these changes in drinking behaviors (Andersen et al., 2014). By the same token, reductions in water intake can also be an early warning of barn problems that need immediate attention. Monitoring water disappearance in a barn should be considered an essential management tool in ABF production.

Water can be provided to pigs using a variety of delivery devices; a major difference among them is their predisposition to wastage (Brumm et al., 2000; Torrey et al., 2008). Well-designed wet-dry feeders appear to most effectively minimize water waste, followed by dish drinkers and then nipple drinkers. The latter are most effective when maintained at the correct height relative to the pig. Swinging nipple drinkers, while also associated with waste, conserved water more effectively than fixed-in-place nipple drinkers (Brumm et al., 2000); however, swinging drinkers are not recommended for newly weaned piglets. Ideally, dish drinkers are the best option for newly weaned pigs. Research shows that pigs find dish drinkers sooner than nipple drinkers and they waste less water. However, dish drinkers can be difficult to keep clean as fouling is a constant concern. Therefore, the best options for wean-to-finish barns are 1) to provide both dish drinkers and nipples permanently installed in the barn, 2) to provide portable dish drinkers that can be moved into pens at the time of weaning; as the pigs find the nipple drinkers, the dish drinkers can be removed. This option includes a significant increase in labor, but given the importance of early water consumption in the newly weaned pig, this can be justified, or 3) to install wet dry feeders as the major source of water and dish drinkers for use during the nursery period.

Achievement of proper flow rates will help to ensure adequate intake while minimizing wastage. The following flow rates are recommended: nursery aged pigs = 500 to 750 mL/min, grow-finish pigs = 750 mL/min and lactating sows = 1,000 mL/min. These flow rates assume, of course, that each pen contains an adequate number of drinking devices. The current recommendation is one drinker per 10 to 15 pigs in wean-to-finish facilities. Sometimes two drinkers are recommended per pen, independent of the number of pigs present, to protect against water deprivation if one drinker fails.

### Reduce Pathogen Load

With the adoption of ABF production, greater emphasis is placed on preventing disease; the option of utilizing antimicrobials when illness occurs is no longer available. However, reducing the pathogen load in a piggery requires much more than improved biosecurity. Every practical means of minimizing disease pressure must be pursued in order to afford the pig the best chance of avoiding illness. Unfortunately, disease prevention is not always possible as endemic diseases in different regions pose a threat to the health of other pigs in the same geographic location.

#### PRRS-free status.

 PRRS was first recognized as a novel disease distinct from other existing illness in 1987 (Hill, 1990). The causative virus was first identified in 1991 in Europe (Terpstra et al., 1991; Wensvoort et al., 1991) and then North America (Collins et al., 1992). It is considered one of the most problematic diseases in pork production, since it not only impairs productivity directly, but also impairs the functioning of the pig’s immune system, rendering it more susceptible to other pathogens (Butler et al., 2014). PRRS virus infections quickly spread systemically, impacting the health and wellbeing of the pig (Lunney et al., 2016). Normally, in the presence of a viral infection, innate defenses slow viral replication, and then trigger an adaptive response involving antibodies and T-cell mediated activities. Infection with the PRRSv is typified by a delayed and faulty innate immune response. Despite the fact that more than 2,000 publications exist on the topic of PRRS, our understanding of the pathogenesis and immunology of PRRSv remains incomplete (Butler et al., 2014). This is at least in part consequential to its high mutation rate due to being an RNA virus. Tools are available to classify herds according to their PRRSv status, to serve as a “road map” to managing herds infected with this pathogen. Such classifications have been used to define treatment regimes, establish vaccination protocols, manage the introduction of new breeding animals into the herd and to establish biosecurity protocols (Holtkamp et al., 2021).

The presence of this virus in a herd makes ABF extremely difficult due to its suppression of the innate immune system, rendering the pig more susceptible to secondary infections. Not only is the pig at greater risk of more infections, but the severity of illness is often greater as well. Combine this with changes in the immune system function at the time of weaning, due in part to the withdrawal of sow’s milk, which contains protective IgA, and the stress of weaning, which may elevate circulating levels of cortisol, further suppression of the immune system is likely to occur. Therefore, it can be seen that PRRSv infection sets off a cascade of events, especially in the newly weaned pig, which result in decreasing maternal antibody protection and increased susceptibility to disease at the very time that the young pig can least adapt.

As technology improves, and our understanding of the physiology and immunology of the pig advances, this restriction may be less important. However, at the present time, PRRSv negative pigs are considered an essential component for success with ABF.

#### Greater emphasis on building hygiene and site biosecurity.

 Reducing the exposure of pigs to all pathogens, as well as the frequency and intensity of that contact, is crucial to ABF success. In its broadest sense, hygiene embraces the dual disciplines of applied microbiology and applied epidemiology, with the ultimate objective of prevention of disease (Madec, 2005). Hygiene is particularly important in the presence of so-called multifactorial respiratory and enteric diseases (Gleeson and Collins, 2015). Emphasis on biosecurity, and associated adherence to a high level of hygiene, is founded on the understanding that prevention of disease is not only financially advantageous, it is also generally far more effective than treatment. This would obviously be very true in ABF production. However, achieving a high level of biosecurity is difficult by its very nature; the pig, the air, people working on or visiting the farm, vehicular traffic, and materials and supplies required by the farm, such as feed and water can all serve as carriers of numerous pathogens.

A high level of biosecurity is required to eliminate or at least minimize the transmission of pathogens from other farms (external biosecurity), or among barns on the same site (internal biosecurity). Its importance has been suggested by studies in numerous countries showing a positive relationship, sometimes objective and sometimes subjective, sometimes strong and sometimes not as strong, between biosecurity and animal welfare and performance: Germany (Raasch et al., 2018); Chantziaris et al., 2020); Ireland (da Costa et al., 2019); and Sweden (Backhans et al., (2015) as well as reports involving multi-country evaluations (Postma et al., 2015; Collineau et al., 2017).

A critical first step in the process is the thorough cleaning, disinfection, and in the heating season, warming of the barn before the pigs arrive. High-pressure washing is practiced to remove all organic material, leading to a clean environment for the newly weaned pigs. Disinfection is practiced to lower the pathogen load in the barn. Warming the barn serves two purposes--it dries the barn to support disinfection and reduces chilling so that the flooring and penning materials are all at room temperature. One of the challenges of ABF production is that it is often used in conjunction with outdoor production, which results in limited abilities to clean and disinfect the environment in which the pigs are raised.

Feed biosecurity cannot be ignored either, given the recent recognition of its potential role in the transmission of viable pathogens such as porcine epidemic diarrhea virus, *Salmonella typhimurium*, and African Swine Fever virus (Cochrane et al., 2016b; Dee et al., 2018; Niederwerder, 2021). The fact that some pathogens can survive for extended periods of time in feed is particularly troubling (Dee et al., 2016, 2018). Contaminated feed can spread pathogens regionally, nationally, or internationally.

Numerous options have been developed to provide protection against viral infections entering a farm via the feed (Niederwerder, 2021). Feed handling may represent one option that could be part of a larger, more comprehensive mitigation plan. For example, storage over an extended period of time has been investigated, but it will likely be most practical for individual ingredients which move from regions known to be infected with the virus of interest rather than mixed feed ready for delivery to the farm. Stoian et al. (2019) reported an average half-life of 12 d for the African Swine Fever virus in select ingredients, such as soybean meal and choline, as well as some feed mixtures such as dry dog food, moist dog food and moist cat food. Another option may be to apply heat treatment known to destroy the virus. In the case of porcine epidemic diarrhea virus, heating of the feed to 120 °C for 16.5 min or to 145 °C for 1.3 min, or applying irradiation, have been shown to be effective (Trudeau et al., 2016). All of the feed handling options are limited because of the risk of re-contamination after the treatment is completed and before it is consumed by the pig.

Alternatively, the use of select feed additives such as a combination of formaldehyde and propionic acid, medium-chain fatty acids, MCFA combined with their monoglycerides, OA, or essential oils (Cochrane et al., 2020; Dee et al., 2014, 2021; Jackman et al., 2020) all provided some degree of protection. However, formaldehyde plus propionic acid, MCFA, and monoglycerides look most promising. Cochrane et al. (2016a) has also shown that MCFA can be highly effective against *Salmonella typhimurium*. Formaldehyde is not available for use in all jurisdictions.

The list of products now available on the market for protection against certain viruses is growing at a very rapid rate (Dee et al., 2021). In the case of feed additives, ingredient composition was found to impact their effectiveness. Lerner et al. (2020) concluded that 0.5% of a blend of MCFA or 0.3% of C8 by itself can be effective against PED virus compared to a control diet. Studies by Niederwerder et al. (2021) suggested that the addition of 0.35% formaldehyde or 0.70% of a 1:1:1 mixture of caproic, caprylic, and capric acids as free fatty acids is probably in the range of that required to reduce the viral titers to below the level of detection. While these results are exciting, additional research is required to lock into the best combination of products required to achieve a satisfactory level of protection across a wide array of ingredient mixtures. Of course, this must all be achieved at a reasonable cost.

#### Strategic vaccination program.

 An effective and focused vaccination program is critical to success in ABF production. Vaccines function at both the level of the individual and the level of the herd. With respect to the former, vaccines provide protection to the individual through an induced immune response which is greater than that expected of a naïve animal. At the herd level, vaccines function to reduce pathogen transmission—the so-called concept of herd immunity. The effectiveness of the vaccine against a specific pathogen will involve three factors: the epidemiology of the pathogen and its transmission potential within the herd, the vaccine efficiency which is reflected in the reduction in susceptibility by the individual to the infection and the reduction in the ability to transmit the pathogen to others, and vaccine coverage (Rose and Andraud, 2017).

The vaccination program should create a robust and diverse immunity within the breeding herd as well as the offspring (Tizard, 2021). The exact nature of the program will depend on the diseases which are endemic to a particular herd or which represent a real threat to the herd if not yet present. However, vaccines cannot be viewed as an alternative to other herd health management practices, but rather as one of the tools used and indeed required to achieve success, especially in ABF production.

#### All-in-all-out production.

Along with a high level of hygiene and biosecurity, a commitment to all-in-all-out production is essential to success in ABF production. It also requires that pigs must be weaned into a barn at a different site (Beaulieu et al., 2006) or at least into a barn separated from the sow barn. As an example, Patience et al. (2000) illustrated the impact of site segregation; they reported that pigs weaned at 12 d of age, but housed off-site relative to the sow herd, were heavier at 56 d of age than pigs weaned at either 12 or 21 d of age but retained in the same building as the sows. This was an unusual finding, because weaning pigs at an older age is normally associated with more rapid growth post-weaning. It therefore illustrates the fundamental value of all-in-all-out production and separation of production sites.

It is equally important to house pigs of a single age on a given site, or at least within a barn on a given site. Mixing pigs of differing ages or pigs from different sources into a single air space places overall health status at a very serious risk (Scheidt et al., 1995). Faccin et al. (2020c) conducted a study of the subsequent performance of pigs based on the order in which they were placed in the barn. The 2,184 pigs were moved into the wean-to-finish barn across four batches of 546 pigs each; deliveries of each batch were separated by 8 d. The performance of the pigs was followed through to harvest. Interestingly, there was a clear decline in performance of the pigs according to their order of entering the barn; final market weight and gain:feed declined and the sum of mortalities and pulls increased, all in a linear fashion, as their order of entry increased. This reaffirmed that pigs perform better when entering a clean, new air space, or looked at from the opposite perspective, pigs performed more poorly when entering an air space already occupied by pigs (*P* < 0.05). Holding smaller pigs from one group back in the nursery in order to afford them time to “catch up” is not recommended because it leads to mixing pigs of different ages and facilitates disease transmission. For smaller farms where the sow herd cannot fill the whole nursery, batch farrowing is recommended and has proven to be highly effective (Brown, 2006).

### Health Management

There are certain minimum standards that must be adhered to in order to minimize mortality and morbidity in swine herds. These standards become critically important to success in ABF production systems. For example, it is crucial to understand that in various instances, pathogens can be transmitted from animals to animals, animals to humans, and humans to animals; in some cases, different species can transmit disease to pigs, even if they do not demonstrate symptoms of infection. When attempting to manage pathogen exposure, it is important to understand the many factors which can influence pathogen survival, including temperature, humidity, light, and pH.

Mitigating external risks has, as its objective, preventing the introduction of a new disease or strain of an existing disease into a barn (Ramirez and Zaabel, 2012; da Silva et al., 2018). This would include: 1) maximizing the distance of separation from other swine farms to minimize pathogen movement by air, water, insects, birds, rodents, and wildlife, 2) sourcing all pigs in a nursery fill from one sow herd, preferably one with a high health status, 3) isolation from outside pathogen sources through the use of shower-in-shower-out protocols, control of fomite entry, and down-time since prior exposure to other swine prior to barn entry, 4) farm security through the use of security fences and locked doors to prevent inadvertent entry of visitors, pets, wild animals, and birds, 5) the use of quarantine procedures to facilitate the entry of new breeding stock if a sow farm, 6) following strict protocols to clean and disinfect trucks travelling between farms and packing plants, auction barns and other public animal holding facilities, 7) careful sourcing of feed, as it is now known that feed can act as a vector for certain pathogens, 8) paying attention to water as a possible source of contamination, especially if surface water is being used, and 9) careful sourcing of semen to ensure that it is free of pathogens (Widowski et al., 2003; Dee et al., 2004; Dewey et al., 2006; Khan et al., 2013; Laanen et al., 2013; Thomas et al., 2015; Silva et al., 2018; Dee et al., 2021). Of particular interest in the Laanen et al. (2013) study was the negative relationship between a farm’s level of biosecurity and the need for prophylactic use of antimicrobials. This would be particularly relevant to herds transitioning to ABF production. Finally, when physical separation among farms is not possible, and therefore the risk of aerosol transmission is high, the use of HEPA filtration has been proposed as a viable although expensive option (Perez et al., 2015).

In contrast to addressing external threats to biosecurity, internal risk management is focused on minimizing the spread of disease already present within a barn (Ramirez and Zaabel, 2012). This would address internal health management issues within the hog barn, and include 1) minimizing the range in ages of pigs within a room or preferably within a nursery or wean-to-finish barn site, 2) minimizing cross-fostering among sows, and definitely not cross-fostering after the piglet is 24 h old, 3) frequent removal of manure to minimize contact with animals, 4) timely removal, incineration or composting of mortalities, and 5) adherence to cleaning and disinfection protocols, especially between room or barn fills (Ramirez and Zaabel, 2012; Laanen et al., 2013). The use of vaccines is an effective way to control internal spreading of disease. The use of disinfectants to safely bring fomites into a barn as well as control internal spread of pathogens within a barn can be particularly useful if managed strategically.

## CONCLUSIONS

This review of practices that are recommended to achieve success in ABF production confirms the multidimensional approach that is required: 1) ideally, establishment of a herd which is free of the PRRSv, 2) adoption of one or more feed additives that have been scientifically proven to contribute to a high level of animal performance and well-being, or to support improved gut health, 3) maintenance of a high level of building hygiene and disease management combined with heightened attention to biosecurity, 4) adoption of a proactive approach to health management including a well-designed vaccination program, 5) selection of housing systems combined with husbandry practices which minimize social stressors on the pig, 6) adoption of a weaning age greater than 24 and possibly as high as 28 d, 7) selection of robust genetics that are more resistant to disease and less affected by common stressors, 8) installation and operation of an effective ventilation system to ensure a high quality environment within the barn, 9) formulation of diets which consider the functional properties of dietary ingredients in addition to their supply of energy and nutrients, 10) maintenance of an abundant supply of high quality drinking water, 11) implementation of effective individual perinatal care of the sow and her offspring and of individual pigs at weaning.

One measure of success in ABF production could be the proportion of pigs that remain ABF from birth through to market. If there is a premium price placed on such animals—and there must be due to the higher cost of production—maximizing the number of animals sold through the ABF portal is a means to maximize net income.

Further research is clearly required on the mode of action of new feed additives and especially the new generation of feed additives. In the past, the provision of antibiotics in the feed, for example, had the benefit of being effective under a wide array of conditions; non-antibiotic feed additives do not possess the same degree of universality as reflected by the differences in phenotypic outcome across studies. In the same vein, further research is required on the underlying causes of illness or other forms of stress resulting from different approaches to raising pigs; this review has clearly demonstrated that there is no single “one size fits all” when it comes to successfully managing pigs in an ABF environment.

## References

[CIT0001] AAFCO. 2018. Midyear meeting agenda book. Champaign (IL): Amer. Assoc. Feed Control Officials.

[CIT0002] AASV. 2022. Basic guidelines of judicious therapeutic use of antimicrobials in swine. https://www.aasv.org/documents/JUG.php. Accessed March 17, 2022.

[CIT0003] Aarestrup, F. M., V. F. Jensen, H. -D. Emborg, E. Jacobsen, and H. C. Wegener. 2010. Changes in the use of antimicrobials and the effects on productivity of swine farms in Denmark. Am. J. Vet. Res. 71:726–733. doi:10.2460/ajvr.71.7.726. 20594073

[CIT0004] Acosta, J. A., A. Petry, S. A. Gould, C. K. Jones, C. R. Stark, A. Fahrenholz, and J. F. Patience. 2019. Enhancing digestibility of corn fed to pigs at two stages of growth through management of particle size using a hammermill or a roller mill. Transl. Anim. Sci. 4:10–21. doi:10.1093/tas/txz146. 32704962 PMC7200395

[CIT0005] Acosta, J. A., A. Petry, S. A. Gould, C. K. Jones, C. R. Stark, A. Fahrenholz, and J. F. Patience. 2020. Effects of grinding method and particle size of wheat grain on energy and nutrient digestibility in growing and finishing pigs. Transl. Anim. Sci. 4:682–693. doi:10.1093/tas/txaa062. PMC7344114 32705057

[CIT0006] Allee, G. L., D. R. Romsos, G. A. Leveille, and D. H. Baker. 1972. Metabolic consequences of dietary medium-chain triglycerides in the pig. Proc. Soc. Exp. Biol. Med. 139:422–428. doi:10.3181/00379727-139-36158. 5059032

[CIT0007] Amachawadi, R. G., F. Giok, X. Shi, J. Soto, S. K. Narayanan, M. D. Tokach, M. D. Apley, and T. G. Nagaraja. 2018. Antimicrobial resistance of *Enterococcus faecium* strains isolated from commercial probiotic products used in cattle and swine. J. Anim. Sci. 96:912–920. doi:10.1093/jas/sky056. 29584914 PMC6093546

[CIT0008] Andersen, H. M., L. Dybkjær, and M. S. Herskin. 2014. Growing pigs’ drinking behaviour: number of visits, duration, water intake and diurnal variation. Animal 8:1881–1888. doi:10.1017/S175173111400192X. 25075605

[CIT0009] Anil, L., S. S. Anil, and J. Deen. 2007. Effects of allometric space allowance and weight group composition on grower-finisher pigs. Can. J. Anim. Sci. 87:139–151. doi:10.4141/A06-051.

[CIT0010] AOAC International. 1995. Official methods of analysis, 16th ed. Gaithersburg (MD): Assoc. Off. Anal. Chem.

[CIT0011] Appleby, M. C. 1996. Can we extrapolate from intensive to extensive conditions? Appl. Anim. Behaviour Sci. 49:23–27. doi:10.1016/0168-1591(95)00664-8.

[CIT0012] Araújo, V. de O., R. A. de, M. de F Oliveira, A. Vieira, H. Silveira, L. S. Fonseca, L. K. S. Alves, E. B. B. Guimarães, A. P. Schinckel, and C. A. P. Garbossa. 2020. Bump feed for gestating sows is really necessary? Livest. Sci 240:104184. doi:10.1016/j.livsci.2020.104184.

[CIT0013] Augspurger, N. R., J. D. Spencer, D. M. Webel, and D. H. Baker. 2004. Pharmacological zinc levels reduce the phosphorus-releasing efficacy of phytase in young pigs and chickens. J. Anim. Sci. 82:1732–1739. doi:10.2527/2004.8261732x. 15217001

[CIT0014] AVMA. 2020. Antimicrobial resistant pathogens affecting animal health in the United States. Schaumburg (IL): Committee on Antimicrobials, Amer. Vet. Med. Assoc.

[CIT0015] Bach Knudsen, K. E. 2001. The nutritional significance of “dietary fibre” analysis. Anim. Feed Sci. Technol. 90:3–20. doi: 10.1016/S0377-8401(01)00193-6.

[CIT0016] Backhans, A., M. Sjölund, A. Lindberg, and U. Emanuelson. 2015. Biosecurity level and health management practices in 60 Swedish farrow-to-ﬁnish herds. Acta Vet. Scand. 57:14. doi:10.1186/s13028-015-0103-5. 25887040 PMC4359795

[CIT0017] Bagger, J., S. Mikkelson, and H. Bak. 2015. Production of antibiotic-free pigs – tools and results. Ames (IA): Proc. Int’l. Conf. Epidem. Control Biol. Chem. Physic. Hazards Pigs Pork. Iowa State Univ. p. 131–134. doi:10.31274/safepork-180809-263.

[CIT0018] Baker, R. 2002. Two sides of producing antibiotic-free pork. St. Paul (MN): Proc. Allen D. Leman Swine Conf., Univ. Minn. p. 87–88.

[CIT0019] Baker, R. 2006. Health management with reduced antibiotic use – the U.S. Experience. Anim. Biotechnol. 17:195–206. doi:10.1080/10495390600962274. 17127530

[CIT0020] Barba-Vidal, E., S. Martín-Orúe, and L. Castillejos. 2018. Review: are we using probiotics correctly in post-weaning piglets? Animal 12:2489–2498. doi:10.1017/S1751731118000873. 29720287

[CIT0021] Batson, K. L., H. I. Calderón, M. D. Tokach, J. C. Woodworth, R. D. Goodband, S. S. Dritz, and J. M. DeRouchey. 2021. Effects of feeding diets containing low crude protein and coarse wheat bran as alternatives to zinc oxide in nursery pig diets. J. Anim. Sci. 99:skab090. doi:10.1093/jas/skab090. 33755175 PMC8269968

[CIT0022] Beal, J. D., S. J. Niven, and P. H. Brooks. 2004. The effect of copper on the death rate of *Salmonella typhimurium* DT104:30 in food substrates acidified with organic acids. Letters Appl. Microbiol. 38:8–12. doi:10.1046/j.1472-765X.2003.01147.x. 14687208

[CIT0023] Beaulieu, A. D., J. Aalhus, N. H. Williams, and J. F. Patience. 2010. Impact of piglet birth weight, birth order and litter size on subsequent growth performance, carcass quality, muscle composition and eating quality of pork. J. Anim. Sci. 88:2767–2778. doi:10.2527/jas.2009-2222. 20418451

[CIT0024] Beaulieu, A. D., C. L. Levesque, and J. F. Patience. 2006. The effects of dietary energy concentration and weaning site on weanling pig performance. J. Anim. Sci. 84:1159–1168. doi:10.2527/2006.8451159x. 16612018

[CIT0025] Bedford, M. R., and H. V. Masey O’Neill. 2016. Extending the value of the literature: Data requirements for holo-analysis and interpretation of outputs. In: M. R. Bedford, M. Choct, and H. Masey O’Neill, editors, Nutrition experiments in pigs and poultry: a practical guide. Wallingford (UK): CABI. p. 121–137.

[CIT0026] Bednorz, C., K. Oelgeschläger, B. Kinnemann, S. Hartmann, K. Neumann, R. Pieper, A. Bethe, T. Semmler, K. Tedin, P. Schierack, et al. 2013. The broader context of antibiotic resistance: zinc feed supplementation of piglets increases the proportion of multi-resistant Escherichia coli in vivo. Int. J. Med. Microbiol. 303:396–403. doi:10.1016/j.ijmm.2013.06.004. 23856339

[CIT0027] Behnke, K. C. 1996. Feed manufacturing technology: current issues and challenges. Anim. Feed Sci. Technol. 62:49–57. doi:10.1016/S0377-8401(96)01005-X.

[CIT0028] Berghof, T. V., M. Poppe, and H. A. Mulder. 2019. Opportunities to improve resilience in animal breeding programs. Front. Genet. 9:692. doi:10.3389/fgene.2018.00692. 30693014 PMC6339870

[CIT0029] Biancone, L., I. Monteleone, G. D. V. Blanco, P. Vavassori, and F. Pallone. 2002. Resident bacterial flora and immune system. Dig. Liver Dis. 34:S37–S43. doi:10.1016/S1590-8658(02)80162-1. 12408438

[CIT0030] Bikker, P., and A. Dirkzwager. 2003. Withdrawal of antimicrobial growth promoters from pig diets increases the amino acid requirements for growth performance. In W. B. Souffrant, C. Metges, editors, Progr. Res. Ener. Prot. Metabol. Wageningen (The Netherlands): Wageningen Academic Publishers; p. 593–596.

[CIT0031] Bikker P. , A. Dirkzwager, J. Fledderus, P. Trevisi, I. le Huerou-Luron, J. P. Lallès, and A. Awati. 2006. The effect of dietary protein and fermentable carbohydrates levels on growth performance and intestinal characteristics in newly weaned piglets. J. Anim. Sci. 84:3337–3345. doi:10.2527/jas.2006-076. 17093226

[CIT0032] Bikker, P., A. W. Jongbloed, and J. van Baal. 2016. Dose-dependent effects of copper supplementation of nursery diets on growth performance and fecal consistency in weaned pigs. J. Anim. Sci. 94:181–186. doi:10.2527/jas.2015-9874.

[CIT0033] Bindels, L. B., N. M. Delzenne, P. D. Cani, and J. Walter. 2015. Towards a more comprehensive concept for prebiotics. Nat. Rev. Gastroenterol. Hepatol. 12:303–310. doi:10.1038/nrgastro.2015.47. 25824997

[CIT0034] Blank, R., R. Mosenthin, W. C. Sauer, and S. Huang. 1999. Effect of fumaric acid and dietary buffering capacity on ileal and fecal amino acid digestibilities in early-weaned pigs. J. Anim. Sci. 77:2974–2984. doi:10.2527/1999.77112974x. 10568467

[CIT0035] Blecha, F., D. S. Pollman, and D. A. Nichols. 1983. Weaning pigs at an early age decreases cellular immunity. J. Anim. Sci. 56:396–400. doi:10.2527/jas1983.562396x. 6841290

[CIT0036] Boddicker, N., E. H. Waide, R. R. R. Rowland, J. K. Lunney, D. J. Garrick, J. M. Reecy, and J. C. M. Dekkers. 2012. Evidence for a major QTL associated with host response to porcine reproductive and respiratory syndrome virus challenge. J. Anim. Sci. 90:1733–1746. doi:10.2527/jas.2011-4464. 22205662

[CIT0037] Boyd, R. D., C. E. Zier-Rush, A. J. Moeser, M. Culbertson, K. R. Stewart, D. S. Rosero, and J. F. Patience. 2019. Invited review: innovation through research in the North American pork industry. Animal 13:2951–2966. doi:10.1017/S1751731119001915. 31426881 PMC6874321

[CIT0038] Braun, K., and K. de Lange. 2004. Liquid swine feed ingredients: nutritional quality and contaminants. Proc. ANAC Eastern Nutrition Conference. 17pp.

[CIT0039] Brown, P. 2006. Advantages and disadvantages of batch farrowing. In Practice 28:94–96. doi:10.1136/inpract.28.2.94.

[CIT0040] Bruininx, E. M. A. M., G. P. Binnendijk, C. M. C. van der Peet-Schwering, J. W. Schrama, L. A. den Hartog, H. Everts, and A. C. Beynen. 2002. Effect of creep feed consumption on individual feed intake characteristics and performance of group-housed weanling pigs. J. Anim. Sci. 80:1413–1418. doi:10.2527/2002.8061413x. 12078720

[CIT0041] Bruininx, E. M. A. M., C. M. C. van der Peet-Schwering, J. W. Schrama, P. F. G. Vereijken, P. C. Vesseur, H. Everts, L. A. den Hartog, and A. C. Beynen. 2001. Individually measured feed intake characteristics and growth performance of group-housed weanling pigs: effects of sex, initial body weight, and body weight distribution within groups. J. Anim. Sci. 79:301–308. doi:10.2527/2001.792301x. 11219437

[CIT0042] Brumm, M. C. 2012. Impact of heavy market weights on facility and equipment needs. St. Paul (MN): Proc. Allen D. Leman Swine Conf. Univ. Minnesota. p. 165–168.

[CIT0043] Brumm, M. C., J. M. Dahlquist, and J. M. Heemstra. 2000. Impact of feeders and drinker devices on pig performance, water use and manure volume. J. Swine Health Prod. 8:51–57.

[CIT0044] Brundige, D. R., E. A. Maga, K. C. Klasing, and J. D. Murray. 2008. Lysozyme transgenic goats’ milk influences gastrointestinal morphology in young pigs. J. Nutr. 138:921–926. doi:10.1093/jn/138.5.921. 18424602

[CIT0045] Brüssow, H. 2017. Adjuncts and alternatives in the time of antibiotic resistance and in-feed antibiotic bans. Micro. Biotechnol. 10:674–677. doi:10.1111/1751-7915.12730. PMC5481517 28643479

[CIT0046] Bryden, W. L. 2012. Mycotoxin contamination of the feed supply chain: implications for animal productivity and feed security. Anim. Feed Sci. Technol. 173:134–158. doi:10.1016/j.anifeedsci.2011.12.014.

[CIT0047] Burdick Sanchez, N. C., P. R. Broadway, and J. A. Carroll. 2021. Influence of yeast products on modulating metabolism and immunity in cattle and swine. Animals 11:371. doi:10.3390/ani11020371. 33540746 PMC7913008

[CIT0048] Butler, J. E., K. M. Lager, W. Golde, K. S. Faaberg, M. Sinkora, C. Loving, and Y. I. Zhang. 2014. Porcine reproductive and respiratory syndrome (PRRS): an immune dysregulatory pandemic. Immunol. Res. 29:81–108. doi:10.1007/s12026-014-8549-5. PMC7091131 24981123

[CIT0049] Callaway, T. R., T. S. Edrington, A. Brabban, B. Kutter, L. Karriker, C. Stahl, E. Wagstrom, R. Anderson, T. L. Poole, K. Genovese, et al. 2011. Evaluation of phage treatment as a strategy to reduce *salmonella* populations in growing swine. Foodborne Pathog. Dis. 8:261–266. doi:10.1089/fpd.2010.0671. 21034249

[CIT0050] Calleson, J. 2004. Effects of termination of AGP-use on pig welfare and productivity. In Beyond Antibiotic Growth Promoters in Food Animal Production: Working Papers From the International Symposium. DIAS Report – Animal Husbandry 57:57–60.

[CIT0051] Callewaert, L., and C. W. Michiels. 2010. Lysozymes in the animal kingdom. J. Biosci. 35:127–160. doi:10.1007/s12038-010-0015-5. 20413917

[CIT0052] Canani, R. B., M. Di Costanzo, L. Leone, M. Pedata, R. Meli, and A. Calignano. 2011. Potential beneficial effects of butyrate in intestinal and extraintestinal diseases. World J. Gastroenterol. 17:1519–1528. doi:10.3748/wjg.v17.i12.1519. 21472114 PMC3070119

[CIT0053] Canibe, N., and B. B. Jensen. 2003. Fermented and nonfermented liquid feed to growing pigs: effect on aspects of gastrointestinal ecology and growth performance. J. Anim. Sci. 81:2019–2031. doi:10.2527/2003.8182019x. 12926784

[CIT0054] Cappai, M. G., M. Picciau, and W. Pinna. 2013. Ulcerogenic risk assessment of diets for pigs in relation to gastric lesion prevalence. BMC Vet. Res. 9:36–44. doi:10.1186/1746-6148-9-36. 23432961 PMC3598992

[CIT0055] Carver, J. D., and W. A. Walker. 1995. The role of nucleotides in human nutrition. J. Nutr. Biochem. 6:58–72. doi:10.1016/0955-2863(94)00019-I.

[CIT0056] CDC. 2013. Antibiotic Resistance Threats in the United States, 2013. Washington (DC): U.S. Department of Health and Human Services, Centers for Disease Control and Prevention.

[CIT0057] CDC. 2019. Antibiotic use in the United States, 2018 Update: Progress and Opportunities. Atlanta (GA): U.S. Dept of Health and Human Services.

[CIT0058] CDC. 2020. Antibiotic/antimicrobial resistance. (AR/AMR). Atlanta (GA): Centers for Disease Control and Prevention.

[CIT0059] CFIA. 2018. How is the CFIA contributing to the responsible use of medically important antimicrobials in animals? Canadian Food Inspection Agency, Government of Canada, Ottawa. (Last revised April 3, 2018).

[CIT0060] Chance, J. A., J. M. DeRouchey, R. G. Amachawadi, V. Ishengoma, T. G. Nagaraja, R. D. Goodband, J. C. Woodworth, M. D. Tokach, H. I. Calderón, Q. Kang, et al. 2021. Live yeast and yeast extracts with and without pharmacological levels of zinc on nursery pig growth performance and antimicrobial susceptibilities of fecal *Escherichia coli*. J. Anim. Sci. 99:skab330. doi:10.1093/jas/skab330. 34752618 PMC8664753

[CIT0061] Chantziaris, I., J. Dewulf, T. Van Limbergen, T. Stadejek, J. Niemi, I. Kyriazakis, and D. Maes. 2020. Biosecurity levels of pig fattening farms from four EU countries and links with the farm characteristics. Livestock Sci. 237:104037. doi:10.1016/j.livsci.2020.104037.

[CIT0062] Che, L., Q. Xu, C. Wu, Y. Luo, X. Huang, B. Zhang, E. Auclair, T. Kiros, Z. Fang, Y. Lin, et al. 2017. Effects of dietary live yeast supplementation on growth performance, diarrhoea severity, intestinal permeability and immunological parameters of weaned piglets challenged with enterotoxigenic *Escherichia coli* K88. Br. J. Nutr. 118:949–958. doi:10.1017/S0007114517003051. 29166952

[CIT0063] Chen, H. H., Y. K. Chen, H. C. Chang, and S. Y. Lin. 2012. Immunomodulatory effects of xylooligosaccharides. Food Sci. Technol. Res. 18:195–199. doi:10.3136/fstr.18.195.

[CIT0064] Choi, J., L. Wang, S. Liu, P. Lu, X. Zhao, H. Liu, L. Lahaye, E. Santin, S. Liu, M. Nyachoti, et al. 2020. Effects of a microencapsulated formula of organic acids and essential oils on nutrient absorption, immunity, gut barrier function, and abundance of enterotoxigenic *Escherichia coli* F4 in weaned piglets challenged with E. coli F4. J. Anim. Sci. 98:skaa259. doi:10.1093/jas/skaa259. 32780110 PMC7526869

[CIT0065] Christian, L. L. 1972. A review of the role of genetics in animal stress susceptibility and meat quality. Madison (WI): Proc. Pork Qual. Symp. Univ. Wisconsin. p. 91–115.

[CIT0066] Ciesinski, L., S. Guenther, R. Pieper, M. Kalisch, C. Bednorz, and L. H. Wieler. 2018. High dietary zinc feeding promotes persistence of multi-resistant E. coli in the swine gut. PLoS One. 13(1):e0191660. doi:10.1371/journal.pone.0191660. 29373597 PMC5786291

[CIT0067] Clouard, C., C. Le Bourgot, F. Respondek, J. E. Bolhuis, and W. J. J. Gerrits. 2018. A milk formula containing maltodextrin, vs. Lactose, as main carbohydrate source, improves cognitive performance of piglets in a spatial task. Sci. Rep. 8:9433. doi:10.1038/s41598-018-27796-1. 29930401 PMC6013478

[CIT0068] Cochrane, R. A., S. S. Dritz, J. C. Woodworth, C. R. Stark, A. R. Huss, J. P. Cano, R. W. Thompson, A. C. Fahrenholz, and C. K. Jones. 2016a. Feed mill biosecurity plans: a systematic approach to prevent biological pathogens in swine feed. J. Swine Health Prod. 24:154–164

[CIT0069] Cochrane, R. A., S. S. Dritz, J. C. Woodworth, C. R. Stark, M. Saensukjaroenphon, J. T. Gebhardt, J. Bai, R. A. Hesse, E. G. Poulsen, Q. Chen, et al. 2020. Assessing the effects of medium-chain fatty acids and fat sources on PEDV infectivity. Transl. Anim. Sci 4:1051–1059. doi:10.1093/tas/txz179. PMC7107285 32289114

[CIT0070] Cochrane, R. A., A. R. Huss, G. C. Aldrich, C. R. Stark, and C. K. Jones. 2016b. Evaluating chemical mitigation of *Salmonella* typhimurium ATCC 14028 in animal feed ingredients. J. Food Prot. 79:672–676. doi:10.4315/0362-028X.JFP-15-320. 27052874

[CIT0071] Colditz, I. G., and B. C. Hine. 2016. Resilience in farm animals: biology, management, breeding and implications for animal welfare. Anim. Prod. Sci 56:1961–1983. doi:10.1071/AN15297.

[CIT0072] Collineau, L., A. Backhans, J. Dewulf, U. Emanuelson, E. grosse Beilage, A. Lehébel, S. Loesken, E. Okholm Nielsen, M. Postma, M. Sjölund, K. D. C. Stärk, and C. Belloc. 2017. Profile of pig farms combining high performance and low antimicrobial usage within four European countries. Vet. Rec. 181:657. doi:10.1136/vr.103988. 29051316

[CIT0073] Collins, J. E., D. A. Benfield, W. T. Christianson, L. Harris, J. C. Hennings, D. P. Shaw, S. M. Goyal, S. McCullogh, R. B. Morrison, H. S. Joo, et al. 1992. Isolation of swine infertility and respiratory syndrome virus (isolate ATCC VR-2332) in North America and experimental reproduction of the disease in gnotobiotic pigs. J. Vet. Diagn. Invest. 4:117–126. doi:10.1177/104063879200400201. 1616975

[CIT0074] Columbus, D., and C. F. M. de Lange. 2012. Evidence for validity of ileal digestibility coefficients in monogastrics.Brit. J. Nutr. 108:S264–S272. doi:10.1017/S0007114512002334. 23107537

[CIT0075] Condotta, I. C. S. S., T. M. Brown-Brandl, J. P. Stinn, G. A. Rohrer, J. D. Davis, and K. O. Silva-Miranda. 2018. Dimensions of the modern pig. Trans. ASABE 61:1729–1739. doi:10.13031/trans.12826.

[CIT0076] Cooper, C. A., L. C. Garas Klobas, E. A. Maga, and J. D. Murray. 2013. Consuming transgenic goats’ milk containing the antimicrobial protein lysozyme helps resolve diarrhea in young pigs. PLoS One 8:e58409. doi:10.1371/journal.pone.0058409. 23516474 PMC3596375

[CIT0077] Cornelison, A. S., L. A. Karriker, N. H. Williams, B. J. Haberl, K. J. Stalder, L. L. Schulz, and J. F. Patience. 2018. Impact of health challenges on pig growth performance, carcass characteristics and net returns under commercial conditions. Transl. Anim. Sci 2:50–61. doi:10.1093/tas/txx005. 32289106 PMC7107292

[CIT0078] Craig, J. R., F. R. Dunshea, J. J. Cottrell, E. M. Ford, U. A. Wijesiriwardana, and J. R. Pluske. 2019. Feeding conjugated linoleic acid without a combination of medium-chain fatty acids during late gestation and lactation improves pre-weaning survival rates of gilt and sow progeny. Animals 9:62. doi:10.3390/ani9020062. PMC6406248 30781377

[CIT0079] Cromwell, G. L. 2002. Why and how antibiotics are used in swine production. Anim. Biotechnol. 13:7–27. doi:10.1081/ABIO-120005767. 12212945

[CIT0080] Cromwell, G. L., M. D. Lindemann, H. J. Monegue, D. D. Hall, and D. E. Orr, Jr. 1998. Tribasic copper chloride and copper sulfate as copper sources for weanling pigs. J. Anim. Sci. 76:118–123. doi:10.2527/1998.761118x. 9464892

[CIT0081] Costa, M. R., J. Gasa, J. A. C. Diaz, M. Postma, J. Dewulf, G. McCutcheon, and E. G. Manzanilla. 2019. Using the Biocheck. UGent™ scoring tool in Irish farrow-to-finish pig farms: assessing biosecurity and its relation to productive performance. Porc. Health Manag. 5:4. doi:10.1186/s40813-018-0113-6. PMC6396494 30867936

[CIT0082] Dallas, D. C., V. Weinborn, J. M. de Moura Bell, M. Wang, E. A. Parker, A. Guerrero, K. A. Hettinga, C. B. Lebrilla, J. B. German, and D. Barile. 2014. Comprehensive peptidomic and glycomic evaluation reveals that sweet whey permeate from colostrum is a source of milk protein-derived peptides and oligosaccharides. Food Res. Int. 63:203–209. doi:10.1016/j.foodres.2014.03.021. 25284962 PMC4180214

[CIT0083] Decuypere, J., and N. Dierick. 2003. The combined use of triacylglycerols containing medium-chain fatty acids and exogenous lipolytic enzymes as an alternative to in-feed antibiotics in piglets: concept, possibilities and limitations. An overview. Nutr. Res. Rev. 16:193–210. doi:10.1079/NRR200369. 19087389

[CIT0084] DeDecker, J. M., M. Ellis, B. F. Wolter, B. P. Corrigan, S. E. Curtis, and G. R. Hollis. 2005. Effect of stocking rate on pig performance in a wean-to-finish production system. Can. J. Anim. Sci. 85:1–5. doi:10.4141/A04-042.

[CIT0085] Dee, S., J. Deen, D. Burns, G. Douthit, and C. Pijoan. 2004. An assessment of sanitation protocols for commercial transport vehicles contaminated with porcine reproductive and respiratory syndrome virus. Can. J. Vet. Res. 68:208–214. 15352546 PMC1142141

[CIT0086] Dee, S., J. E. Guzman, D. Hanson, N. Garbes, R. Morrison, D. Amodie, and L. G. Pantoja. 2018. A randomized controlled trial to evaluate performance of pigs raised in antibiotic-free or conventional production systems following challenge with porcine reproductive and respiratory syndrome virus. PLoS One 13:e0208430. doi:10.1371/journal.pone.0208430. 30521587 PMC6283559

[CIT0087] Dee, S., C. Neill, T. Clement, J. Christopher-Hennings, and E. Nelson. 2014. An evaluation of a liquid antimicrobial (Sal CURB ®) for reducing the risk of porcine epidemic diarrhea virus infection of naïve pigs during consumption of contaminated feed. BMC Vet. Res. 10:220. doi:10.1186/s12917-014-0220-9. 25253192 PMC4179854

[CIT0088] Dee, S., C. Neill, A. Singrey, T. Clement, R. Cochrane, C. Jones, G. Patterson, G. Spronk, J. Christopher-Hennings, and E. Nelson. 2016. Modeling the transboundary risk of feed ingredients contaminated with porcine epidemic diarrhea virus. BMC Vet. Res. 2016:51. doi:10.1186/s12917-016-0674-z. PMC4788872 26968372

[CIT0089] Dee, S., M. C. Niederwerder, R. Edler, D. Hanson, A. Singrey, R. Cochrane, G. Spronk, and E. Nelson. 2021. An evaluation of additives for mitigating the risk of virus-contaminated feed using an ice-block challenge model. Transbound. Emerg. Dis. 68:833–845. doi:10.1111/tbed.13749. 32706431 PMC8247034

[CIT0090] Delaney, R. A. M. 1975. The nutritive value of porcine blood amino acids in dried blood concentrates prepared by utrafiltration and spray drying. J. Sci. Food Agric. 26:303–310. doi:10.1002/jsfa.2740260310. 1134068

[CIT0091] De Lange, C. F. M., J. Pluske, J. Gong, and C. M. Nyachoti. 2010. Strategic use of feed ingredients and feed additives to stimulate gut health and development in young pigs. Livestock Sci. 134:124–134. doi:10.1016/j.livsci.2010.06.117.

[CIT0092] Denver, S., J. D. Jensen, and T. Christensen. 2021. Consumer preferences for reduced antibiotic use in Danish pig production. Prev. Vet. Med. 189:105310. doi:10.1016/j.prevetmed.2021.105310. 33667759

[CIT0093] DeRouchey, J. M., J. D. Hancock, R. H. Hines, C. A. Maloney, D. J. Lee, H. Cao, D. W. Dean, and J. S. Park. 2004. Effects of rancidity and free fatty acids in choice white grease on growth performance and nutrient digestibility in weanling pigs. J. Anim. Sci. 82:2937–2944. doi:10.2527/2004.82102937x. 15484945

[CIT0094] Desiree, K., S. Mosimann, and P. Ebner. 2021. Efficacy of phage therapy in pigs: systematic review and meta-analysis. J. Anim. Sci. 99:skab157. doi:10.1093/jas/skab157. 34196704 PMC8280926

[CIT0095] Dewey, C. E., W. T. Johnston, L. Gould, and T. L. Whiting. 2006. Postweaning mortality in Manitoba swine. Can. J. Vet. Res. 70:161–167. 16850937 PMC1477929

[CIT0096] Dibner, J. J., and J. D. Richards. 2005. Antibiotic growth promoters in agriculture: history and mode of action. Poult. Sci. 84:634–643. doi:10.1093/ps/84.4.634. 15844822

[CIT0097] Dierick, N., and J. A. Decurpere. 2002. Endogenous lipolysis in feedstuffs and compound feeds for pigs: effects of storage time and conditions and lipase and/or emulsifier addition. Anim. Feed Sci. Technol. 102:53–70. doi:10.1016/S0377-8401(02)00224-9.

[CIT0098] Domeneghini, C., A. Di Giancamillo, G. Bosi, and S. Arrighi. 2006. Can nutraceuticals affect the structure of intestinal mucosa? qualitative and quantitative microanatomy in L-glutamine diet-supplemented weaning piglets. Vet. Res. Comm. 30:331–342. doi:10.1007/s11259-006-3236-1. 16437309

[CIT0099] Done, S. H., D. J. Chennells, A. C. J. Gresham, S. Williamson, B. Hunt, L. L. Taylor, V. Bland, P. Jones, D. Armstrong, R. P. White, et al. 2005. Clinical and pathological responses of weaned pigs to atmospheric ammonia and dust. Vet. Rec. 157:71–80. doi:10.1136/vr.157.3.71. 16024672

[CIT0100] Dong, G. Z., and J. R. Pluske. 2007. The low feed intake in newly-weaned pigs: problems and possible solutions. Asian-Aust. J. Anim. Sci. 20:440–452. doi:10.5713/ajas.2007.440.

[CIT0101] Dritz, S. S., M. D. Tokach, R. D. Goodband, and J. L. Nelssen. 2002. Effects of administration of antimicrobials in feed on growth rate and feed efficiency of pigs in multisite production systems. J. Amer. Vet. Med. Assoc. 220:1690–1695. doi:10.2460/javma.2002.220.1690. 12051512

[CIT0102] Dunmire, K. M., T. A. Wickersham, L. L. Frenzel, S. R. Sprayberry, L. C. Joiner, L. P. Hernandez, A. M. Cassens, B. Dominguez, and C. B. Paulk. 2020. Effects of adding liquid lactose or molasses to pelleted swine diets on pellet quality and pig performance. Transl. Anim Sci 4:616–629. doi:10.1093/tas/txaa039. PMC7229991 32705036

[CIT0103] Duttlinger, A. W., K. R. Kpodo, D. C. Lay, Jr, B. T. Richert, and J. S. Johnson. 2019. Replacing dietary antibiotics with 0.20% L-glutamine in swine nursery diets: impact on health and productivity of pigs following weaning and transport. J. Anim. Sci. 97:2035–2052. doi:10.1093/jas/skz098. 30924491 PMC6488329

[CIT0104] Duttlinger, A. W., K. R. Kpodo, A. P. Schinckel, B. T. Richert, and J. S. Johnson. 2020. Effects of increasing dietary L-glutamine to replace antibiotics on pig health and performance following weaning and transport. Transl. Anim. Sci 4:txaa157. doi:10.1093/tas/txaa157. 33123679 PMC7575130

[CIT0105] Eklund, T. 1983. The antimicrobial effect of dissociated and undissociated sorbic acid at different pH levels. J. Appl. Bacteriol. 54:383–389. doi:10.1111/j.1365-2672.1983.tb02632.x. 6409875

[CIT0106] Fablet, C., C. Marois, V. Dorenlor, F. Eono, E. Eveno, J. P. Jolly, L. Le Devendec, M. Kobisch, F. Madec, and N. Rose. 2012. Bacterial pathogens associated with lung lesions in slaughter pigs from 125 herds. Res. Vet. Sci. 93:627–630. doi:10.1016/j.rvsc.2011.11.002. 22133708

[CIT0107] Faccin, J. E. G., F. Laskoski, L. F. Hernig, R. Kummer, G. F. R. Lima, U. A. D. Orlando, M. A. D. Gonçalves, A. P. G. Mellagi, R. R. Ulguim, and F. P. Bortolozzo. 2020a. Impact of increasing weaning age on pig performance and belly nosing prevalence in a commercial multisite production system. J. Anim. Sci. 98 (4):skaa031. doi:10.1093/jas/skaa031. 32034395 PMC7183176

[CIT0108] Faccin, J. E. G., F. Laskoski, M. Quirino, M. A. D. Goncalves, U. A. D. Orlando, A. P. G. Mellagi, M. L. Bernardi, R. R. Ulguim, and F. P. Bertolozzo. 2020b. Impact of housing nursery pigs according to body weight on the onset of feed intake, aggressive behavior, and growth performance. Trop. Anim. Health Prod. 52:1073–1079. doi:10.1007/s11250-019-02096-6. 31701398

[CIT0109] Faccin, J. E. G., M. D. Tokach, M. W. Allerson, J. C. Woodworth, J. M. DeRouchey, S. S. Dritz, F. P. Bortolozzo, and R. D. Goodband. 2020c. Relationship between weaning age and antibiotic usage on pig growth performance and mortality. J. Anim. Sci. 98:skaa363. doi:10.1093/jas/skaa363. 33188416 PMC7755175

[CIT0110] FAO/WHO. 2002. Guidelines for the evaluation of probiotics in food. Report of a joint Food and Agricultural Organization of the United Nations/World Health Organization-working group on drafting guidelines for the evaluation of probiotics in food, London, Ontario, Canada.

[CIT0111] Fairbairn, S. L., J. F. Patience, H. L. Classen, and R. T. Zijlstra. 1999. The energy content of barley fed to growing pigs: characterizing the nature of its variability and developing prediction equations for its estimation. J. Anim. Sci. 77:1502–1512. doi:10.2527/1999.7761502x. 10375227

[CIT0112] Farmer, C., and S. A. Edwards. 2020. The neonatal pig: developmental influences on vitality. In: C. Farmer, editor, The Suckling and Weaned Pig. Wageningen (The Netherlands): Wageningen Academic Press. p. 9–39. doi:10.3920/978-90-8686-894-0.

[CIT0113] FDA. 2018. 2017 Summary report on antimicrobials sold and distributed for use in food producing animals. Washington (DC): Food and Drug Administration.

[CIT0114] Ferrari, C. V., P. E. Sbardella, M. L. Bernardi, M. L. Coutinho, I. S. Vaz, Jr, I. Wentz, and F. P. Bertolozzo. 2014. Effect of birth weight and colostrum intake on mortality and performance of piglets after cross-fostering in sows of different parities. Prev. Veterin. Med. 114:259–266. doi:10.1016/j.prevetmed.2014.02.013. 24674020

[CIT0115] Feyera, T., T. F. Pedersen, U. Krogh, L. Foldager, and P. K. Theil. 2018. Impact of sow energy status during farrowing on farrowing kinetics, frequency of stillborn piglets, and farrowing assistance. J. Anim. Sci. 96:2320–2331. doi:10.1093/jas/sky141. 29684197 PMC6095272

[CIT0116] Flohr, J. R., J. M. DeRouchey, J. C. Woodworth, M. D. Tokach, R. D. Goodband, and S. S. Dritz. 2016. A survey of current feeding regimens for vitamins and trace minerals in the US swine industry. J. Swine Health Prod. 6:290–303.

[CIT0117] Fouhse, J. M., R. T. Zijlstra, and B. P. Willing. 2016. The role of gut microbiota in the health and disease of pigs. Anim. Front 6:30–36. doi:10.2527/af.2016-0031.

[CIT0118] Franz, C., K. H. C. Baser, and W. Windisch. 2010. Essential oils and aromatic plants in animal feeding – a European perspective. A review. Flavour Fragr. J. 25:327–340. doi:10.1002/ffj.1967.

[CIT0119] Fraser, D., J. F. Patience, P. A. Phillips, and J. M. McLeese. 1993. Water for piglets and lactating sows: quantity, quality and quandaries. In: D. J. A. W. Haresign, and P. C. Garnsworthy, editors, Recent Developments in Pig Nutrition 2. Nottingham University Press. p. 201–224.

[CIT0120] Fraser, D., and P. A. Phillips. 1989. Lethargy and low water intake by sows during early lactation: a cause of low piglet weight gains and survival. Appl. Anim. Behav. Sci. 24:3–22. doi:10.1016/0168-1591(89)90121-4.

[CIT0121] FSIS. 2017. FY 2017 Residue Sample Results. Washington (DC): Food Safety and Inspection Service, United States Department of Agriculture. 50 pp.

[CIT0122] Garas, L. C., C. A. Cooper, M. W. Dawson, J. -L. Wang, J. D. Murray, and E. A. Maga. 2017. Young pigs consuming lysozyme transgenic goat milk are protected from clinical symptoms of enterotoxigenic *Escherichia coli* infection. J. Nutr. 147:2050–2059. doi:10.3945/jn.117.251322. 28954839

[CIT0123] Gebhardt, J. T., K. A. Thomson, J. C. Woodworth, S. S. Dritz, M. D. Tokach, J. M. DeRouchey, R. D. Goodband, C. K. Jones, R. A. Cochrane, M. C. Niederwerder, et al. 2020a. Effect of dietary medium-chain fatty acids on nursery pig growth performance, fecal microbial composition, and mitigation properties against porcine epidemic diarrhea virus following storage. J. Anim. Sci. 98:skz358. doi:10.1093/jas/skz358. 31758795 PMC6978897

[CIT0124] Gebhardt, J. T., M. D. Tokach, S. S. Dritz, N. Cernicchiaro, A. L. Dixon, D. G. Renter, J. C. Woodworth, J. M. DeRouchey, and R. D. Goodband. 2020b. Practical application of sample size determination models for assessment of mortality outcomes in wine field trials. Kansas Agricultural Experiment Station Research Reports 6(10). doi:10.4148/2378-5977.8004.

[CIT0125] Gerrits, W. J. J., M. W. Bosch, and J. J. G. C. van den Borne. 2012. Quantifying resistant starch using novel, in vivo methodology and the energetic utilization of fermented starch in pigs. J. Nutr. 142:238–244. doi:10.3945/jn.111.147496. 22223577

[CIT0126] Gibson, G. R. 1999. Dietary modulation of the human gut microflora using the prebiotics oligofructose and inulin. J. Nutr. 129:1438S–1441S. doi:10.1093/jn/129.7.1438S. 10395616

[CIT0127] Gleeson, B. L., and A. M. Collins. 2015. Under what conditions is it possible to produce pigs without using antibiotics. Anim. Prod. Sci 55:1424–1431. doi:10.1071/AN15271.

[CIT0128] Gonyou, H. W., M. C. Brumm, E. Bush, J. Deen, S. A. Edwards, T. Fangman, J. J. McGlone, M. Meunier-Salaun, R. B. Morrison, H. Spoolder, et al. 2006. Application of broken-line analysis to assess floor space requirements of nursery and grower-finisher pigs expressed on an allometric basis. J. Anim. Sci. 84:229–235. doi:10.2527/2006.841229x. 16361511

[CIT0129] Gonyou, H. W. 1998. Sorting and mixing grower/finisher pigs. Saint Paul (MN): Proc. Allen D. Leman Swine Conf. Univ. Minnesota. p. 126–128.

[CIT0130] González-Ortiz, G., G. A. Gomes, T. T. dos Santos, and M. R. Bedford. 2019. New strategies influencing gut functionality and animal performance. In: G. González-Ortiz, M. R. Bedford, K. E. Bach Knudsen, C. M. Courtin, and H. L. Classen, editors, The Value of Fibre: Engaging the Second Brain for Animal Nutrition. Wageningen (The Netherlands): Wageningen Academic Publishers. p. 343–349 doi:10.3920/978-90-8686-893-3.

[CIT0131] González-Ortiz, G., J. F. Pérez, R. G. Hermes, F. Molist, R. Jiménez-Díaz, and S. M. Martín-Orúe. 2014. Screening the ability of natural feed ingredients to interfere with the adherence of enterotoxigenic *Escherichia coli* (ETEC) K88 to the porcine intestinal mucus. Br. J. Nutr. 111:633–642. doi:10.1017/S0007114513003024. 24047890

[CIT0132] Greiner, L. L., T. S. Stahly, and T. J. Stabel. 2001. The effect of dietary soy genistein on pig growth and viral replication during a viral challenge. J. Anim. Sci. 79:1272–1279. doi:10.2527/2001.7951272x. 11374547

[CIT0133] Grinstead, G. S., R. D. Goodband, S. S. Dritz, M. D. Tokach, J. L. Nelssen, J. C. Woodworth, and M. Molitor. 2000. Effects of a whey protein product and spray-dried animal plasma on growth performance of weanling pigs. J. Anim. Sci. 78:647–657. doi:10.2527/2000.783647x. 10764072

[CIT0134] Hajati, H. 2018. Application of organic acids in poultry nutrition. Int. J. Avian Wildlife Biol. 3:324–329. doi:10.15406/ijawb.2018.03.00114.

[CIT0135] Halas, D., J. M. Heo, C. F. Hansen, C. F. Kim, D. J. Hampson, B. P. Mullan, and J. R. Pluske. 2007. Organic acids, prebiotics and protein level as dietary tools to control the weaning transition and reduce post-weaning diarrhoea in piglets. CAB Rev. 2:79. doi:10.1079/PAVSNNR20072079.

[CIT0136] Hanczakowska, E. 2017. The use of medium-chain fatty acids in piglet feeding–A review. Ann. Anim. Sci 17:967–977. doi:10.1515/aoas-2016-0099.

[CIT0137] Harris, D. L. 1988. Alternative approaches to eliminating endemic diseases and improving performance of pigs. Vet. Rec. 123:422–423. doi:10.1136/vr.123.16.422. 3201671

[CIT0138] Hastad, C. H., M. D. Tokach, S. S. Dritz, R. D. Goodband, J. M. Derouchey, and F. Wu. 2020. Effects of added fat on growth performance of finishing pigs sorted by initial weight. Transl. Anim. Sci. 4:307–315. doi:10.1093/tas/txz162. 32704990 PMC7200418

[CIT0139] Healy, B. J., J. D. Hancock, G. A. Kennedy, P. J. Bramel-Cox, K. C. Behnke, and R. H. Hines. 1994. Optimum particle size of corn and hard and soft sorghum for nursery pigs. J. Anim. Sci. 72:22274–22236. doi:10.2527/1994.7292227x. 8002441

[CIT0140] Helm, E. T., N. K. Gabler, and E. R. Burrough. 2021. Highly fermentable fiber alters fecal microbiota and mitigates swine dysentery induced by brachyspira hyodysenteriae. animals 11:396. doi:10.3390/ani11020396. 33557345 PMC7915590

[CIT0141] Heo, J. M., J. C. Kim, C. F. Hansen, B. P. Mullan, D. J. Hampson, and J. R. Pluske. 2009. Feeding a diet with decreased protein content reduces indices of protein fermentation and the incidence of postweaning diarrhea in weaned pigs challenged with an enterotoxigenic strain of *Escherichia coli*. J. Anim. Sci. 87:2833–2843. doi:10.2527/jas.2008-1274. 19502498

[CIT0142] Heo, J. M., F. O. Opapeju, J. R. Pluske, J. C. Kim, D. J. Hampson, and C. M. Nyachoti. 2013. Gastrointestinal health and function in weaned pigs: a review of feeding strategies to control post-weaning diarrhoea without using in-feed antimicrobial compounds. J. Anim. Physiol. Anim. Nutr 97:207–237. doi:10.1111/j.1439-0396.2012.01284.x. 22416941

[CIT0143] Hermes, R. G., F. Molist, M. Ywazaki, M. Nofrarías, A. Gomez de Segura, J. Gasa, and J. F. Pérez. 2009. Effect of dietary level of protein and fiber on the productive performance and health status of piglets. J. Anim. Sci. 87:3569–3577. doi:10.2527/jas.2008-1241. 19648494

[CIT0144] Herpin, P., M. Damon, and J. Le Dividich. 2002. Development of thermoregulation and neonatal survival in pigs. Livest. Prod. Sci. 78:25–45. doi:10.1016/S0301-6226(02)00183-5.

[CIT0145] van Heugten, E., D. S. Rosero, P. L. Chang, C. Zier-Rush, and R. D. Boyd. 2016. Peroxidized lipids in nursery pig diets – why and when should we be concerned? Indianapolis (IN): Proc. Midwest Swine Nutr. Conf. p. 31–39.

[CIT0146] Hill, H. 1990. Overview and history of mystery swine disease (swine infertility/respiratory syndrome). In: Proc. Mystery Swine Disease Committee Meeting. Denver (CO): Livestock Conservation Institute. pp. 29–31.

[CIT0147] Hill, C., F. Guarner, G. Reid, G. R. Gibson, D. J. Merenstein, B. Pot, L. Morelli, R. B. Canani, H. J. Flint, S. Salminen, et al. 2014. The international scientific association for probiotics and prebiotics consensus statement on the scope and appropriate use of the term probiotic. Nat. Rev. Gastroenterol. Hepatol. 11:506–514. doi:10.1038/nrgastro.2014.66. 24912386

[CIT0148] Højberg, O., N. Canibe, H. D. Poulsen, M. S. Hedemann, and B. B. Jensen. 2005. Influence of dietary zinc oxide and copper sulfate on the gastrointestinal ecosystem in newly weaned piglets. Appl. Environ. Microbiol. 71:2267–2277. doi:10.1128/AEM.71.5.2267-2277.2005. 15870311 PMC1087531

[CIT0149] Holscher, H. D. 2017. Dietary fiber and prebiotics and the gastrointestinal microbiota. Gut Microbes 8:172–184. doi:10.1080/19490976.2017.1290756. 28165863 PMC5390821

[CIT0150] Holtkamp, D. J., J. B. Kliebenstein, E. J. Neumann, J. Zimmerman, H. R. Rotto, T. K. Yoder, C. Wang, P. Yeske, C. Mowrer, and C. Haley. 2013. Assessment of the economic impact of porcine reproductive and respiratory syndrome virus on United States pork producers. J. Swine Health Prod. 21:72–84.

[CIT0151] Holtkamp, D., M. Torremorell, C. A. Corzo, D. C. L. Linhares, M. N. Almeida, P. Yeske, D. D. Polson, L. Becton, H. Snelson, T. Donovan, et al. 2021. Proposed modifications to porcine reproductive and respiratory syndrome virus herd classification. J. Swine Health Prod 29:261–270.

[CIT0152] Hossan, S., K. S. Huque, K. M. Kamaruddin, and A. H. Beg. 2018. Global restriction of using antibiotic growth promoters and alternative strategies in poultry production. Sci. Prog. Chem. Org. Nat. Prod. 101:52–75. doi:10.3184/003685018X15173975498947. PMC10365203 29467062

[CIT0153] Hughes, P., and J. Heritage. 2004. Antibiotic growth-promoters in food animals. Assessing Quality and Safety of Animal Feeds. Rome (Italy): Food and Agriculture Organization of the United Nations. p. 129–152.

[CIT0154] Humphrey, B. D., and K. C. Klasing. 2003. Modulation of nutrient metabolism and homeostasis by the immune system. Lillehammer (Germany): Proc. 14th Eur. Symp. Poult. Nutr. p. 137–144.

[CIT0155] Hunger, C., C. Schwarz, C. Schieder, B. Rueel, K. Schedle, and A. Faouën. 2017. Efficacité d’un additif alimentaire phytogénique chez des porcs charcutiers nourris avec un aliment à faible teneur en protéines. J. Rech. Porc. 49:107–108.

[CIT0156] Huntley, N. F., C. M. Nyachoti, and J. F. Patience. 2018. Lipopolysaccharide immune stimulation but not β-mannanase supplementation affects maintenance energy requirements in young weaned pigs. J. Anim. Sci. Biotechnol. 9:901–916. doi:10.1186/s40104-018-0264-y. PMC6003148 29946460

[CIT0157] Hurley, W. L. 2015. Composition of sow colostrum and milk. In: C. Farmer, editor, The gestating and lactating sow. Wageningen (The Netherlands): Wageningen Academic Publishers. p. 193–230. doi:10.3920/978-90-8686-803-2.

[CIT0158] Hutchens, W. M., M. D. Tokach, J. C. Woodworth, J. M. DeRouchey, R. D. Goodband, J. T. Gebhardt, H. I. Calderon, K. Keppy, P. Maynard, and E. Grilli. 2021. Evaluation of microencapsulated organic acids and botanicals on growth performance of nursery and growing-finishing pigs. Transl. Anim. Sci 5:txab205. doi:10.1093/tas/txab205. 34761168 PMC8576442

[CIT0159] Huting, A. M. S., I. Wellock, S. Tuer, and I. Kyriazakis. 2019. Weaning age and post weaning nursery feeding regime are important in improving the performance of lightweight pigs. J. Anim. Sci. 97:4834–4844. doi:10.1093/jas/skz337. 31679028 PMC6915233

[CIT0160] Hyun, Y., and M. Ellis. 2002. Effect of group size and feeder type on growth performance and feeding patterns in finishing pigs. J. Anim. Sci. 80:568–574. doi:10.2527/2002.803568x. 11890394

[CIT0161] Hyun, Y. M., R. Ellis, and W. Johnson. 1998. Effects of feeder type, space allowance, and mixing on the growth performance and feed intake pattern of growing pigs. J. Anim. Sci. 76:2771–2778. doi:10.2527/1998.76112771x. 9856385

[CIT0162] Jackman, J. A., R. D. Boyd, and C. C. Elrod. 2020. Medium-chain fatty acids and monoglycerides as feed additives for pig production: towards gut health improvement and feed pathogen mitigation. J. Anim. Sci. Biotechnol. 11:44. doi:10.1186/s40104-020-00446-1. 32337029 PMC7178611

[CIT0163] Jacobsen, L. D. 2012a. How to economically cool pig buildings. Salta (Argentina): Proc. XI Nat’l Cong. Swine Prod.

[CIT0164] Jacobsen, L. D. 2012b. 21st century ventilation and energy management. Nat’l Hog Farmer. www.nationalhogfarmer.com/facilities/21st-century-ventilation-and-energy-management. Accessed April 12, 2021.

[CIT0165] Jamalludeen, N., R. P. Johnson, P. E. Shewen, and C. L. Gyles. 2009. Evaluation of bacteriophages for prevention and treatment of diarrhea due to experimental enterotoxigenic *Escherichia coli* O149 infection of pigs. Vet. Microbiol. 136:135–141. doi:10.1016/j.vetmic.2008.10.021. 19058927

[CIT0166] Jang, K. B., J. M. Purvis, and S. W. Kim. 2021. Dose–response and functional role of whey permeate as a source of lactose and milk oligosaccharides on intestinal health and growth of nursery pigs. J. Anim. Sci. 99:skab008. doi:10.1093/jas/skab008. 33521816 PMC7849970

[CIT0167] Jasaitis, D. K., J. E. Wohlt, and J. L. Evans. 1987. Influence of feed ion content on buffering capacity of ruminant feedstuffs in vitro. J. Dairy Sci. 70:1391–1403. doi:10.3168/jds.S0022-0302(87)80161-3.

[CIT0168] Jaworski, N. W., and H. H. Stein. 2017. Disappearance of nutrients and energy in the stomach and small intestine, cecum, and colon of pigs fed corn-soybean meal diets containing distillers dried grains with solubles, wheat middlings, or soybean hulls. J. Anim. Sci. 95:727–739. doi:10.2527/jas2016.0752. 28380581

[CIT0169] Jeaurond, E. A., M. Rademacher, J. R. Pluske, C. H. Zhu, and C. F. M. de Lange. 2008. Impact of feeding fermentable proteins and carbohydrates on growth performance, gut health and gastrointestinal function of newly weaned pigs. Can. J. Anim. Sci. 88:271–281. doi:10.4141/CJAS07062.

[CIT0170] Jensen, J., C. K. Nielsen, J. Vinther, and R. B. D’Eath. 2012. The effect of space allowance for finishing pigs on productivity and pen hygiene. Livestock Sci. 149:33–40. doi:10.1016/j.livsci.2012.06.018.

[CIT0171] Jha, R., and J. F. D. Berrocoso. 2015. Review: dietary fiber utilization and its effects on physiological functions and gut health of swine. Animal 9:1441–1452. doi:10.1017/S1751731115000919. 25997437 PMC4574174

[CIT0172] Jobling, M. 1995. Simple indices for the assessment of the influences of social environment on growth performance, exemplified by studies on Arctic char. Aquaculture Int. 3:60–65.

[CIT0173] Johnson, C. 2018. Raised without antibiotics: analyzing the impact of biological and economic performance. Proc. Banff Pork Sem. 29:215–222.

[CIT0174] Johnson, J. S., and D. C. Lay, Jr. 2017. Evaluating the behavior, growth performance, immune parameters, and intestinal morphology of weaned piglets after simulated transport and heat stress when antibiotics are eliminated from the diet or replaced with L-glutamine. J. Anim. Sci. 95:91–102. doi:10.2527/jas.2016.1070. 28177383

[CIT0175] Johnson, R., C. Gyles, W. Huff, S. Ojha, G. Huff, N. Rath, and A. Donoghue. 2008. Bacteriophages for prophylaxis and therapy in cattle, poultry and pigs. Anim. Health Res. Rev. 9:201–215. doi:10.1017/S1466252308001576. 19102791

[CIT0176] Jukes, T. H., E. L. R. Stokstad, R. R. Tayloe, T. J. Cunha, H. M. Edwards, and G. B. Meadows. 1950. Growth promoting effect of aureomycin on pigs. Arch. Biochem 26:324–330. 15411210

[CIT0177] Kahn, L. H., G. Bergeron, M. W. Bourassa, B. De Vegt, J. Gill, F. Gomes, F. Malouin, K. Opengart, G. D. Ritter, R. S. Singer, C. Storrs, and E. Topp. 2019. From farm management to bacteriophage therapy: strategies to reduce antibiotic use in animal agriculture. Ann. N. Y. Acad. Sci. 1441:31–39. doi:10.1111/nyas.14034. 30924542 PMC6850639

[CIT0178] Kar, S. K., A. J. M. Jansman, S. Boeren, L. Kruijt, and M. A. Smits. 2016. Protein, peptide, amino acid composition, and potential functional properties of existing and novel dietary protein sources for monogastrics. J. Anim. Sci. 94:30–39. doi:10.2527/jas.2015-9677.

[CIT0179] Karavolias, J., M. J. Salois, K. T. Baker, and K. Watkins. 2018. Raised without antibiotics: impact on animal welfare and implications for food policy. Transl. Anim. Sci 2:337–348. doi:10.1093/tas/txy016. 32704717 PMC7200433

[CIT0180] Kasper, C., D. Ribeiro, A. M. De Almeida, C. Larzul, and E. Murani. 2020. Application in animal science—a special emphasis on stress response and damaging behaviour in pigs. Genes 11:920. doi:10.3390/genes11080920. PMC7464449 32796712

[CIT0181] Kats, L. J., J. L. Nelssen, M. D. Tokach, R. D. Goodband, J. A. Hansen, and J. L. Laurin. 1994. The effect of spray-dried porcine plasma on growth performance in the early-weaned pig. J. Anim. Sci. 72:2075–2081. doi:10.2527/1994.7282075x. 7982837

[CIT0182] Kellner, T. A., and J. F. Patience. 2017. The digestible energy, metabolizable energy, and net energy content of dietary fat sources in thirteen and fifty-kilogram pigs. J. Anim. Sci. 95:3984–3995. doi:10.2527/jas2017.1824. 28992014

[CIT0183] Kerr, B. J., T. A. Kellner, and G. C. Shurson. 2015. Characteristics of lipids and their feeding value in swine diets. J. Anim. Sci. Biotechnol. 6:300. doi:10.1186/s40104-015-0028-x. PMC4512021 26207182

[CIT0184] Khan, S. U., K. R. Atanasova, S. W. S. Krueger, A. Ramirez, and G. C. Gray. 2013. Epidemiology, geographical distribution, and economic consequences of swine zoonoses. Emerg Microbes Infect 2:1–11. doi:10.1038/emi.2013.87. PMC3880873 26038451

[CIT0185] Kiarie, E. G., and A. Mills. 2019. Role of feed processing on gut health and function in pigs and poultry: conundrum of optimal particle size and hydrothermal regimens. Front. Vet. Sci 6:19. doi:10.3389/fvets.2019.00019. 30838217 PMC6390496

[CIT0186] Kiarie, E., B. A. Slominski, D. O. Krause, and C. M. Nyachoti. 2009. Gastrointestinal ecology response of piglets fed diets containing non-starch polysaccharide hydrolysis products and egg yolk antibodies following an oral challenge with *Escherichia coli* (K88). Can. J. Anim. Sci. 89:341–352. doi:10.4141/CJAS09008.

[CIT0187] Kil, D. Y., and H. H. Stein. 2010. Board invited review: management and feeding strategies to ameliorate the impact of removing antibiotic growth promoters from diets fed to weanling pigs. Can. J. Anim. Sci. 90:447–460. doi:10.4141/CJAS10028.

[CIT0188] Kim, J., B. P. Mullan, D. J. Hampson, and J. R. Pluske. 2008. Addition of oat hulls to an extruded rice-based diet for weaner pigs ameliorates the incidence of diarrhoea and reduces indices of protein fermentation in the gastrointestinal tract. Br. J. Nutr. 99:1217–1225. doi:10.1017/S0007114507868462. 18042308

[CIT0189] Kim, S. J., C. H. Kwon, B. C. Park, C. Y. Lee, and J. H. Han. 2015. Effects of a lipid-encapsulated zinc oxide dietary supplement, on growth parameters and intestinal morphology in weanling pigs artificially infected with enterotoxigenic *Escherichia coli*. J. Anim. Sci. Technol. 57:4. doi:10.1186/s40781-014-0038-9. 26290724 PMC4540299

[CIT0190] King, L. M., G. V. Sanchez, M. Bartoces, L. A. Hicks, and K. Fleming-Dutra. 2018. Antibiotic therapy in US adults with sinusitis. J.A.M.A. Intern. Med. 178:992–994. doi:10.1001/jamainternmed.2018.0407. PMC5885211 29582085

[CIT0191] Kirbis, A., and M. Krizman. 2015. Spread of antibiotic resistant bacteria from food of animal origin to humans and vice versa. Proc. Food Sci. 5:148–151. doi:10.1016/j.profoo.2015.09.043.

[CIT0192] Kirchhelle, C. 2018. Swann Song: Antibiotic regulation in British livestock production (1953-2006). Bull. Hist. Med. 92:317–351. doi:10.1353/bhm.2018.0029. 29961717

[CIT0193] Klopatek, S., T. R. Callaway, T. Wickersham, T. G. Sheridan, and D. J. Nisbet. 2021. Bacteriophage utilization in animal hygiene. In: D. R. Harper, S. T. Abedon, B. H. Burrowes, and M. L. McConville, editors, Bacteriophages. Cham (Switzerland): Springer. Doi: 10.1007/978-3-319-41986-2_30

[CIT0194] Knap, P. W., and A. Doeschl-Wilson. 2020. Why breed disease-resilient livestock, and how? Genet. Sel. Evol. 52:60. doi:10.1186/s12711-020-00580-4. 33054713 PMC7557066

[CIT0195] Krause, D. O., S. K. Bhandari, J. D. House, and C. M. Nyachoti. 2010. Response of nursery pigs to a synbiotic preparation of starch and an anti-*Escherichia coli* K88 probiotic. Appl. Environ. Microbiol. 76:8192–8200. doi:10.1128/AEM.01427-10. 20952649 PMC3008263

[CIT0196] Laanen, M., D. S. Persoons, E. Ribbens, B. de Jong, M. Callens, D. Strubbe, D. Maes, and J. Dewulf. 2013. Relationship between biosecurity and production/antimicrobial treatment characteristics in pig herds. Vet. J. 198:508–512. doi:10.1016/j.tvjl.2013.08.029. 24268483

[CIT0197] Lagos, L. V., S. A. Lee, M. R. Bedford, and H. H. Stein. 2021. Reduced concentrations of limestone and monocalcium phosphate in diets without or with microbial phytase did not influence gastric pH, fecal score, or growth performance, but reduced bone ash and serum albumin in weanling pigs. Transl. Anim. Sci 5:txab115. doi:10.1093/tas/txab115. 34377950 PMC8345825

[CIT0198] Lallès, J. P., P. Bosi, H. Smidt, and C. R. Stokes. 2007. Nutritional management of gut health in pigs around weaning. Proc. Nutr. Soc. 66:260–268. doi:10.1017/S0029665107005484. 17466106

[CIT0199] Lallès, J. P., G. Boudry, C. Favier, N. Le Floc’h, I. Luron, L. Montagne, I. P. Oswald, S. Pié, C. Piel, and B. Sève. 2004. Gut function and dysfunction in young pigs: physiology. Anim. Res. 53:301–316. doi:10.1051/animres:2004018.

[CIT0200] Lallès, J. -P., and C. A. Montoya. 2021. Dietary alternatives to in-feed antibiotics, gut barrier function and inflammation in piglets post-weaning: where are we now? Anim. Feed Sci. Technol. 274:114836. doi:10.1016/j.anifeedsci.2021.114836.

[CIT0201] Lambo, M. T., C. Xiaofeng, and D. Liu. 2021. The recent trend in the use of multistrain probiotics in livestock production: an overview. Animals 11:2805. doi:10.3390/ani11102805. 34679827 PMC8532664

[CIT0202] Lange, S., and I. Lonnroth. 2001. The anti-secretory factor: synthesis, anatomical and cellular distribution, and biological action in experimental and clinical studies. Int’l Rev. Cytol. 210:39–75. doi:10.1016/S0074-7696(01)10003-3. 11580208

[CIT0203] Larivière, E. L., C. Zhu, S. Zettell, R. Patterson, N. A. Karrow, and L. -A. Huber. 2021. The effect of deoxynivalenol-contaminated corn and an immune-modulating feed additive on growth performance and immune response of nursery pigs fed corn- and soybean meal-based diets. Transl. Anim. Sci 5:txab141. doi:10.1093/tas/txab141. 34611597 PMC8485908

[CIT0204] Larson, Z., B. Subramanyam, and T. Herman. 2008. Stored-product insects associated with eight feed mills in the midwestern United States. J. Econ. Entomol. 101:998–1005. doi:10.1093/jee/101.3.998. 18613605

[CIT0205] Laskoski, F., J. E. G. Faccin, C. M. Vier, M. A. D. Goncalves, U. A. D. Orlando, R. Kummer, A. P. G. Mellagi, M. L. Bernardi, I. Wentz, and F. P. Bortolozzo. 2019. Effects of pigs per feeder hole and group size on feed intake onset, growth performance, and ear and tail lesions in nursery pigs with consistent space allowance. J. Swine Health Prod. 27:12–18.

[CIT0206] Lassok, B., and B. -A. Tenhagen. 2013. From pig to pork: methicillin-resistant Staphylococcus aureus in the pork production chain. J. Food Protection 76:1095–1108. doi:10.4315/0362-028X.JFP-12-341. 23726208

[CIT0207] Lawlor, P. G., G. E. Gardiner, and R. D. Goodband. 2020. Feeding the weaned pig. In: C. Farmer, editor, The suckling and weaned pig. Wageningen (The Netherlands): Wageningen Academic Press. p. 251–275 doi:10.3920/978-90-8686-894-0.

[CIT0208] Lawlor, P. G., P. B. Lynch, and P. J. Caffrey. 2006. Effect of fumaric acid, calcium formate and mineral levels in diets on the intake and growth performance of newly weaned pigs. Irish J. Agric. Food Res 45:61–71.

[CIT0209] Lawlor, P. G., P. B. Lynch, P. J. Caffrey, J. J. O’Reilly, and M. K. O’Connel. 2005. Measurements of the acid-binding capacity of ingredients used in pig diets. Irish Vet. J. 58:447–452. doi:10.1186/2046-0481-58-8-447. PMC3113917 21851673

[CIT0210] Lee, S., A. Hosseindoust, A. Goel, Y. Choi, I. K. Kwonand, and B. Chae. 2016. Effects of dietary supplementation of bacteriophage with or without zinc oxide on the performance and gut development of weanling pigs. Italian J. Anim. Sci. 15:412–418. doi:10.1080/1828051X.2016.1188676.

[CIT0211] Le Floc’h, N., A. G. Wessels, E. Corrent, G. Wu, and P. Bosi. 2018. The relevance of functional amino acids to support the health of growing pigs. Anim. Feed Sci. Technol. 245:104–116. doi:10.1016/j.anifeedsci.2018.09.007.

[CIT0212] Leliveld, L. M. C., A. V. Riemensperger, G. E., Gardiner, J. V. O’Doherty, P. B. Lynch, and P. G. Lawlor. 2013. Effect of weaning age and postweaning feeding programme on the growth performance of pigs to 10 weeks of age. Livest. Sci 157:225–233. doi:10.1016/j.livsci.2013.06.030.

[CIT0213] Lemos Teixeira, D., D. Enriquez-Hidalgo, T. Estay Espinoza, F. Bas, and M. J. Hötzel. 2021. Meat consumers’ opinion regarding unhealthy pigs: should they be treated with antibiotics or euthanized on farm? Antibiotics 10:10010060. doi:10.3390/antibiotics10010060. PMC7826963 33435299

[CIT0214] Lerner, A. B., R. A. Cochrane, J. T. Gebhardt, S. S. Dritz, C. K. Jones, J. M. DeRouchey, M. D. Tokach, R. D. Goodband, J. Bai, E. Porter, et al. 2020. Effects of medium chain fatty acids as a mitigation or prevention strategy against porcine epidemic diarrhea virus in swine feed. J. Anim. Sci. 98:skaa159. doi:10.1093/jas/skaa159. 32447386 PMC7281870

[CIT0215] Li, D. F., J. Y. Jiang, and Y. X. Ma. 2003. Early weaning diets and feed additives. In: R. J. Xu, and P. D. Cranwell, editors, The neonatal pig: gastrointestinal physiology and nutrition. Thrumptom (UK): Nottingham Univ. Press. p. 247–274.

[CIT0216] Li, Q. Y. 2018. Impact of dietary fiber and exogenous carbohydrases in weaned pigs. PhD Diss., Iowa State Univ.

[CIT0217] Li, Q. Y., E. R. Burrough, N. K. Gabler, C. L. Loving, O. Sahin, S. A. Gould, and J. F. Patience. 2019. A soluble and highly fermentable dietary fiber with carbohydrases improved gut barrier integrity markers and growth performance in ETEC challenged pigs. J. Anim. Sci. 97:2139–2153. doi:10.1093/jas/skz093. 30888017 PMC6488326

[CIT0218] Li, Q. Y., N. K. Gabler, C. L. Loving, S. A. Gould, and J. F. Patience. 2018. A dietary carbohydrase blend improved intestinal barrier function and growth rate in nursery pigs fed higher fiber diets. J. Anim. Sci. 96:5233–5243. doi:10.1093/jas/sky383. 30299467 PMC6276555

[CIT0219] Li, Q. Y., X. Peng, E. R. Burrough, O. Sahin, S. A. Gould, N. K. Gabler, C. L. Loving, K. S. Dorman, and J. F. Patience. 2020. Dietary soluble or insoluble fiber with or without enzymes altered the intestinal microbiota in weaned pigs challenged with Enterotoxigenic *E. coli* F18. Front. Microbiol. 11:1110. doi:10.3389/fmicb.2020.01110. 32536908 PMC7267687

[CIT0220] Li, Y., Z. Song, K. A. Kerr, and A. J. Moeser. 2017. Chronic social stress in pigs impairs intestinal barrier and nutrient transporter function, and alters neuro-immune mediator and receptor expression. PLoS One 12:e0171617. doi:10.1371/journal.pone.0171617. 28170426 PMC5295718

[CIT0221] Liao, S. F., and M. Nyachoti. 2017. Using probiotics to improve swine gut health and nutrient utilization. Anim. Nutr 3:331–343. doi:10.1016/j.aninu.2017.06.007. 29767089 PMC5941265

[CIT0222] Lockyer, S., and A. P. Nugent. 2017. Health effects of resistant starch. Nutr. Bull. 42:10–41. doi:10.1111/nbu.12244.

[CIT0223] Lunney, J. K., Y. Fang, A. Ladinig, N. Chen, Y. Li, B. Rowland, and G. J. Renukaradhya. 2016. Porcine reproductive and respiratory syndrome virus (PRRSV): pathogenesis and interaction with the immune system. Annu. Rev. Anim. Biosci. 4:129–154. doi:10.1146/annurev-animal-022114-111025. 26646630

[CIT0224] Lynegaard, J. C., I. Larsen, C. F. Hansen, J. P. Nielsen, and C. Amdi. 2021. Performance and risk factors associated with first antibiotic treatment in two herds, raising pigs without antibiotics. Porc. Health Manag. 7:18. doi:10.1186/s40813-021-00198-y. PMC7888151 33597035

[CIT0225] Madec, F. 2005. The role of animal hygiene and health management in pig production. Ploufragan (France): Int’l Soc. Anim. Hygiene. French Food Safety Agency. p. 40–48.

[CIT0226] Maenner, K., W. Vahjen, and O. Simon. 2011. Studies on the effects of essential-oil-based feed additives on performance, ileal nutrient digestibility, and selected bacterial groups in the gastrointestinal tract of piglets. J. Anim. Sci. 89:2106–2112. doi:10.2527/jas.2010-2950. 21357448

[CIT0227] Main, R. G., S. S. Dritz, M. D. Tokach, R. D. Goodband, and J. L. Nelssen. 2004. Increasing weaning age improves pig performance in a multisite production system. J. Anim. Sci. 82:1499–1507. doi:10.2527/2004.8251499x. 15144093

[CIT0228] Main, R. G., J. Stille, B. Borg, M. Hoogland, and D. Tomky. 2010. A field experience implementing an antibiotic-free program in a commercial production system. Ames (IA): Proc. 18th Ann. Swine Diseases Conf. Swine Practitioners, Iowa State Univ. p. 53–56.

[CIT0229] Mallmann, A. L., E. Camilotti, D. P. Fagundes, C. E. Vier, A. P. G. Mellagi, R. R. Ulguim, M. L. Bernardi, U. A. D. Orlando, M. A. D. Gonçalves, R. Kummer, et al. 2019a. Impact of feed intake during late gestation on piglet birth weight and reproductive performance: a dose-response study performed in gilts. J. Anim. Sci. 97:1262–1272. doi:10.1093/jas/skz017. 30649395 PMC6396255

[CIT0230] Mallmann, A. L., D. P. Fagundes, C. E. Vier, G. S. Oliveira, A. P. G. Mellagi, R. R. Ulguim, M. L. Bernardi, U. A. D. Orlando, R. J. Cogo, and F. P. Bortolozzo. 2019b. Maternal nutrition during early and late gestation in gilts and sows under commercial conditions: impacts on maternal growth and litter traits. J. Anim. Sci. 97:4957–4964. doi:10.1093/jas/skz349. 31742334 PMC6915219

[CIT0231] May, K. D., J. E. Wells, C. V. Maxwell, and W. T. Oliver. 2012. Granulated lysozyme as an alternative to antibiotics improves growth performance and small intestinal morphology of 10-day-old pigs. J. Anim. Sci. 90:1118–1125. doi:10.2527/jas.2011-4297. 22064735

[CIT0232] Mayorga, E. J., S. K. Kvidera, E. A. Horst, M. Al-Qaisi, C. S. McCarthy, M. A. Abeyta, S. Lei, T. H. Elsasser, S. Kahl, T. G. Kiros, et al. 2021. Effects of dietary live yeast supplementation on growth performance and biomarkers of metabolism and inflammation in heat-stressed and nutrient-restricted pigs. Transl. Anim. Sci 5 (2):txab072. doi:10.1093/tas/txab072. 34189415 PMC8223600

[CIT0233] McCaw, M. B. 2000. Effect of reducing crossfostering at birth on piglet mortality and performance during an acute outbreak of porcine reproductive and respiratory syndrome. J. Swine Health Prod. 8:15–21.

[CIT0234] McLamb, B. L., A. J. Gibson, E. L. Overman, C. Stahl, and A. J. Moeser. 2013. Early weaning stress in pigs impairs innate mucosal immune responses to enterotoxigenic *e. coli* challenge and exacerbates intestinal injury and clinical disease. PLoS One 8:e59838. doi:10.1371/journal.pone.0059838. 23637741 PMC3634819

[CIT0235] McLeese, J. M., M. L. Tremblay, J. F. Patience, and G. I. Christison. 1992. Water intake patterns in the weanling pig: effect of water quality, antibiotics and probiotics. Anim. Prod 54:135–142. doi:10.1017/S0003356100020651.

[CIT0236] Meijerink, E., S. Neuenschwander, R. Fries, A. Dinter, H. U. Bertschinger, G. Stranzinger, and P. Vögeli. 2000. A DNA polymorphism influencing α(1,2)fucosyltransferase activity of the pig FUT1 enzyme determines susceptibility of small intestinal epithelium to Escherichia coli F18 adhesion. Immunogenetics 52:129–136. doi:10.1007/s002510000263. 11132149

[CIT0237] Metz, J. H. M., and H. W. Gonyou. 1990. Effect of age and housing conditions on the behavioural and haemolytic reaction of piglets to weaning. Appl. Anim. Behav. Sci. 27:299–309. doi:10.1016/0168-1591(90)90126-X.

[CIT0238] Metzler-Zebeli, B. U. 2022. Porcine gut microbiota and host interactions during the transition from the suckling to postweaning phase. In: M. H. Kogut and G. Zhang, editors, Gut microbiota, immunity, and health in production animals. the microbiomes of humans, animals, plants, and the environment. vol 4. Cham (Switzerland): Springer. doi:10.1007/978-3-030-90303-9_8.

[CIT0239] Meyer-Hamme, S. E., K. C. Lambertz, and M. Gauly. 2016. Does group size have an impact on welfare indicators in fattening pigs. Animal 10:142–149. doi:10.1017/S1751731115001779. 26304017

[CIT0240] Michiels A. , S. Piepers, T. Ulens, N. Van Ransbeeck, R. D. Pozo Sacristán, A. Sierens, F. Haesebrouck, P. Demeyer, and D. Maes. 2015. Impact of particulate matter and ammonia on average daily weight gain, mortality and lung lesions in pigs. Prev. Vet. Med. 121:99–107. doi:10.1016/j.prevetmed.2015.06.011. 26148844

[CIT0241] Millet, S., M. Aluwé, A. Van den Broeke, J. De Boever, B. de Witte, L. Douidah, F. Leen, C. de Cuyper, B. Ampe, and S. De Campeneere. 2018a. The effect of crude protein reduction on performance and nitrogen metabolism in piglets (four to nine weeks of age) fed two dietary lysine levels. J. Anim. Sci. 96:3824–3836. doi:10.1093/jas/sky254. 29939350 PMC6127758

[CIT0242] Millet, S., M. Aluwé, A. Van den Broeke, F. Leen, J. De Boever, and S. De Campeneere. 2018b. Review: pork production with maximal nitrogen efficiency. Animal 12:1060–1067. doi:10.1017/S1751731117002610. 29065938

[CIT0243] Millet, S., S. Kumar, J. De Boever, T. Meyns, M. Aluw e, D. De Brabander, and R. Ducatelle. 2012. Effect of particle size distribution and dietary crude fibre content on growth performance and gastric mucosa integrity of growing-finishing pigs. Veterinary J. 192:316–222. doi:10.1016/j.tvjl.2011.06.037. 21880519

[CIT0244] Moeser, A. J., C. S. Pohl, and M. Rajput. 2017. Weaning stress and gastrointestinal barrier development: implications for lifelong gut health in pigs. Anim. Nutr 3:313–321. doi:10.1016/j.aninu.2017.06.003. 29767141 PMC5941262

[CIT0245] Moeser, A. J., K. A. Ryan, P. K. Nighot, and A. T. Blikslager. 2007. Gastrointestinal dysfunction induced by early weaning is attenuated by delayed weaning and mast cell blockade in pigs. Am. J. Physiol. Gastrointest. Liver Physiol. 293:G413–G421. doi: 10.1152/ajpgi.00304.2006. 17525151

[CIT0246] Molden, D. 2007. Water for food water for life: a comprehensive assessment of water management in agriculture. London (UK): Earthscan.

[CIT0247] Moore, P. R., A. Evenson, T. D. Luckey, E. McCoy, C. A. Elvehjem, and E. B. Hart. 1946. Use of sulfasuxidine, streptothricin and streptomycin in nutrition studies with the chick. J. Biol. Chem. 165:437–441. 20276107

[CIT0248] Moran, K., T. Aumiller, K. Manner, J. Zentek, J. D. van der Klis, and A. Muller. 2020. Phytogenics reduces medical treatment in nursery pigs affected by post-weaning diarrhea. J. Anim. Sci. 98:202–203. doi:10.1093/jas/skaa054.351.

[CIT0249] Moran, K., R. D. Boyd, C. Zier-Rush, P. Wilcock, N. Bajjalieh, and E. van Heugten. 2017. Effects of high inclusion of soybean meal and a phytase superdose on growth performance of weaned pigs housed under the rigors of commercial conditions. J. Anim. Sci. 95:5455–5465. doi:10.2527/jas2017.1789. 29293769 PMC6292292

[CIT0250] Mount, L. E. 1959. The metabolic rate of the new-born pig in relation to environmental temperature and to age. J. Physiol. 147:333–345. doi:10.1113/jphysiol.1959.sp006247. 14424736 PMC1357032

[CIT0251] Nathues, H., P. Alarcon, J. Rushton, R. Jolie, K. Fiebig, M. Jimenez, V. Geurts, and C. Nathues. 2017. Cost of porcine reproductive and respiratory syndrome virus at individual farm level – An economic disease model. Prev. Veter. Med. 142:16–29. doi:10.1016/j.prevetmed.2017.04.006. 28606362

[CIT0252] Nessmith, W. B., M. D. Tokach, R. D. Goodband, and J. L. Nelssen. 1997. Defining quality of lactose sources used in swine diets. J. Swine Health Prod. 5(4):145–149.

[CIT0253] Nguyen, D. H., W. J. Seok, and I. H. Kim. 2020. Organic acids mixture as a dietary additive for pigs—a review. Animals 10:952. doi:10.3390/ani10060952. PMC7341275 32486180

[CIT0254] Niederwerder, M. C. 2021. Risk and mitigation of African swine fever virus in feed. Animals 11:792. doi:10.3390/ani11030792. 33803495 PMC7998236

[CIT0255] Niederwerder, M. C., S. Dee, D. G. Diel, A. M. M. Stoian, L. A. Constance, M. Olcha, V. Petrovan, G. Patterson, A. G. Cino-Ozuna, and R. R. R. Rowland. 2021. Mitigating the risk of African swine fever virus in feed with anti-viral chemical additives. Transbound. Emerg. Dis 68:477–486. doi:10.1111/tbed.13699. 32613713

[CIT0256] Niewold, T. A. 2007. The nonantibiotic anti-inflammatory effect of antimicrobial growth promoters, the real mode of action? a hypothesis. Poult. Sci. 86:605–609. doi:10.1093/ps/86.4.605. 17369528

[CIT0257] NRC. 2012. Nutrient requirements of swine. 11th ed., Washington, DC: National Academy Press.

[CIT0258] Nyachoti, C. M., E. Kiarie, S. K. Bhandari, G. Zhang, and D. O. Krause. 2012. Weaned pig responses to *Escherichia coli* K88 (ETEC) oral challenge when receiving a lysozyme supplement. J. Anim. Sci. 90:252–260. doi:10.2527/jas.2010-3596. 21890507

[CIT0259] Nyachoti, C. M., R. T. Zijlstra, C. F. M. de Lange, and J. F. Patience. 2004. Voluntary feed intake in swine: a review of the main determining factors and potential approaches for accurate predictions. Can. J. Anim. Sci. 84:549–566. doi:10.4141/A04-001.

[CIT0260] Odle, J. 1997. New insights into the utilization of medium-chain triglycerides by the neonate: observations from a piglet model. J. Nutr. 127:1061–1067. doi:10.1093/jn/127.6.1061. 9187618

[CIT0261] Oliver, W. T., and J. E. Wells. 2013. Lysozyme as an alternative to antibiotics improves growth performance and small intestinal morphology in nursery pigs. J. Anim. Sci. 91:3129–3136. doi:10.2527/jas.2012-5782. 23572262

[CIT0262] Oliver, W. T., and J. E. Wells. 2015. Lysozyme as an alternative to growth promoting antibiotics in swine production. J. Anim. Sci. Biotechnol. 6:35. doi:10.1186/s40104-015-0034-z. 26273432 PMC4535397

[CIT0263] Oliver, W. T., J. E. Wells, and C. V. Maxwell. 2014. Lysozyme as an alternative to antibiotics improves performance in nursery pigs during an indirect immune challenge. J. Anim. Sci. 92:4927–4934. doi:10.2527/jas.2014-8033. 25253813

[CIT0264] Olsen, K. M., N. K. Gabler, C. J. Rademacher, K. J. Schwartz, W. P. Schweer, G. G. Gourley, and J. F. Patience. 2018. The effects of group size and sub-therapeutic antibiotic alternatives on growth performance and morbidity of nursery pigs: a model for feed additive evaluation. Transl. Anim. Sci 2:298–310. doi:10.1093/tas/txy068. 32289107 PMC7107267

[CIT0265] Omonijo, F. A., L. Ni, J. Gong, Q. Wang, L. Lahaye, and C. Yang. 2018. Essential oils as alternatives to antibiotics in swine production. Anim. Nutr 4:126–136. doi:10.1016/j.aninu.2017.09.001. 30140752 PMC6104524

[CIT0266] O’Quinn, P. R., S. S. Dritz, R. D. Goodband, M. D. Tokach, J. C. Swanson, J. L. Nelssen, and R. E. Musser. 2001. Sorting growing-finishing pigs by weight fails to improve growth performance or weight variation. J. Swine Health Prod. 9:11–16.

[CIT0267] Owen, B. D., J. M. Bell, C. M. Williams, and R. G. Oakes. 1961. Effects of porcine immunoglobin administration on the survival and serum protein composition of colostrum deprived pigs reared in a non-isolated environment. Can. J. Anim. Sci. 41:236–252. doi:10.4141/cjas61-032.

[CIT0268] Pajor, E. A., D. Fraser, and D. L. Kramer. 1991. Consumption of solid food by suckling pigs: individual variation and relation to weight gain. Appl. Anim. Behav. Sci. 32:139–155. doi:10.1016/S0168-1591(05)80038-3.

[CIT0269] Pandey, K. R., S. R. Naik, and B. V. Vakil. 2015. Probiotics, prebiotics and synbiotics- a review. J. Food Sci. Technol. 52:7577–7587. doi:10.1007/s13197-015-1921-1. 26604335 PMC4648921

[CIT0270] Partanen, K. H., and Z. Mroz. 1999. Organic acids for performance enhancement in pig diets. Nutr. Res. Rev. 12:117–145. doi:10.1079/095442299108728884. 19087448

[CIT0271] Patience, J. F. 1996. Meeting the energy and protein requirements of the high producing sow. Anim. Feed Sci. Tech. 58:49–64. doi:10.1016/0377-8401(95)00873-X.

[CIT0272] Patience, J. F. 2013. Water in swine nutrition. Sustainable Swine Nutrition. L.L. Chiba, ed. Hoboken (NJ): John Wiley & Sons, Inc.. p. 3–22.

[CIT0273] Patience, J. F. 2017. The theory and practice of feed formulation. In: P. Moughan, K. de Lange, and W. Hendriks, editors, Feed evaluation science. Wageningen (The Netherlands): Wageningen Academic Press. p. 457–490.

[CIT0274] Patience, J. F., A. D. Beaulieu, and D. A. Gillis. 2004. The impact of ground water high in sulphates on the growth performance, nutrient utilization, and tissue mineral levels of pigs housed under commercial conditions. J. Swine Health Prod. 12:228–236.

[CIT0275] Patience, J. F., A. Chipman, C. K. Jones, and T. Scheer. 2011. Varying corn particle size distribution affects the digestibility of energy for the growing pig. J. Anim. Sci. 89(E-Suppl. 2):127.

[CIT0276] Patience, J. F., H. W. Gonyou, D. L. Whittington, E. Beltranena, C. S. Rhodes, and A. G. Van Kessel. 2000. Evaluation of site and age of weaning on pig growth performance. J. Anim. Sci. 78:1726–1731. doi:10.2527/2000.7871726x. 10907813

[CIT0277] Patience, J. F. and A. L. Petry. 2019. Susceptibility of fibre to exogenous carbohydrases and impact on performance in swine. In: G. Gonzalez-Ortiz, M. R. Bedford, K. E. Bach, C. Knudsen, C. Courtin, and H. L. Classen, editors, The value of fibre. Engaging the second brain for animal nutrition. Wageningen (The Netherlands): Wageningen Academic Press. p. 101–117.

[CIT0278] Patience, J. F., P. A. Thacker, and C. F. M. de Lange. 1995. Swine nutrition guide, 2nd ed. Saskatoon (SK): Prairie Swine Centre Inc.

[CIT0279] Patience, J. F., J. F. Umboh, R. K. Chaplin, and C. M. Nyachoti. 2005. Nutritional and physiological responses of growing pigs exposed to a diurnal pattern of heat stress. Livestock Prod. Sci. 96:205–214. doi:10.1016/j.livprodsci.2005.01.012.

[CIT0280] Peace, R. M., J. Campbell, J. Polo, J. Crenshaw, L. Russell, and A. Moeser. 2011. Spray-dried porcine plasma influences intestinal barrier function, inflammation, and diarrhea in weaned pigs. J. Nutr. 141:1312–1317. doi:10.3945/jn.110.136796. 21613450

[CIT0281] Petherick, J. C., and S. H. Baxter. 1981. Modelling the static special requirements of livestock. Aberdeen (Scotland): CIGR Section II Seminar.

[CIT0282] Pei, X., Z. Xiao, L. Liu, G. Wang, W. Tao, M. Wang, J. Zou, and D. Leng. 2019. Effects of dietary zinc oxide nanoparticles supplementation on growth performance, zinc status, intestinal morphology, microflora population, and immune response in weaned pigs. J. Sci. Food Agric. 99:1366–1374. doi:10.1002/jsfa.9312. 30094852

[CIT0283] Perez, A. M., P. R. Davies, C. K. Goodell, D. J. Holtkamp, E. Mondaca-Fernández, Z. Poljak, S. J. Tousignant, P. Valdes-Donoso, J. J. Zimmerman, and R. B. Morrison. 2015. Lessons learned and knowledge gaps about the epidemiology and control of porcine reproductive and respiratory syndrome virus in North America. J. Am. Veter. Med. Assoc. 246:1304–1317. doi:10.2460/javma.246.12.1304. 26043128

[CIT0284] Pérez-Bosque, A., J. Polo, and D. Torrallardona. 2016. Spray dried plasma as an alternative to antibiotics in piglet feeds, mode of action and biosafety. Porcine Health Manag 23:16. doi:10.1186/s40813-016-0034-1. PMC5382520 28405442

[CIT0285] Perez-Palencia, J. Y., R. S. Samuel, and C. L. Levesque. 2021. Supplementation of protease to low amino acid diets containing superdose level of phytase for wean-to-finish pigs: effects on performance, postweaning intestinal health and carcass characteristics. Transl. Anim. Sci 5:txab088. doi:10.1093/tas/txab088. 34159298 PMC8212168

[CIT0286] Petry, A. L., and J. F. Patience. 2020. Xylanase supplementation in corn-based swine diets: a review with emphasis on potential mechanisms of action. J. Anim. Sci. 98:skaa318. doi:10.1093/jas/skaa318. 32970148 PMC7759750

[CIT0287] Pettigrew, J. E. 2006. Critical review of acidifiers. Des Moines (IA): National Pork Board.

[CIT0288] PIC. 2019. Wean-to-finish management. Hendersonville (TN): PIC North America.

[CIT0289] Pieper, R., C. Villodre, C. Tudela, M. Taciak, J. Bindelle, J. Pérez, and J. Zentek. 2016. Health relevance of intestinal protein fermentation in young pigs. Anim. Health Res. Rev. 17:137–147. doi:10.1017/S1466252316000141. 27572670

[CIT0290] PigChamp. 2020. Benchmark Magazine. Spring 2020. Accessed Mar. 7, 2021. www.pigchamp.com/flipbooks/benchmark-magazine/2020/USA/index.html#3/z.

[CIT0291] Piva, A. V., V. M. Pizzamiglio, M. Morlacchini, M. Tedeschi, and G. Piva. 2007. Lipid microencapsulation allows slow release of organic acids and natural identical flavors along the swine intestine. J. Anim. Sci. 85:486–493. doi:10.2527/jas.2006-323. 17040943

[CIT0292] Plumed-Ferrer, C., and A. Von Wright. 2009. Fermented pig liquid feed: nutritional, safety and regulatory aspects. J. Appl. Microbiol. 106:351–368. doi:10.1111/j.1365-2672.2008.03938.x. 19016978

[CIT0293] Pluske, J. R. 2013. Feed- and feed additives-related aspects of gut health and development in weanling pigs. J. Anim. Sci. Biotechnol. 4 (1). doi:10.1186/2049-1891-4-1. PMC3623743 23289727

[CIT0294] Pluske, J. R., D. J. Kerton, P. D. C., ranwell, R. G. Campbell, B. P. Mullan, R. H. King, G. N. Power, S. G. Pierzynowski, B. Westrom, et al. 2003. Age, sex, weight at weaning influences organ weight and gastrointestinal development of weanling pigs. Aust. J. Agric. Res. 54:515–527. doi:10.1071/AR02156.

[CIT0295] Pohl, C. S., J. E. Medland, E. Mackey, L. L. Edwards, K. D. Bagley, M. P. DeWilde, K. J. Williams, and A. J. Moeser. 2017. Early weaning stress induces chronic functional diarrhea, intestinal barrier defects, and increased mast cell activity in a porcine model of early life adversity. Neurogastroenterol. Motil. 29:e13118. doi:10.1111/nmo.13118. PMC5650513 28573751

[CIT0296] Postma, M. A., A. Backhans, L. Collineau, S. Loesken, M. Sjölund, C. Belloc, U. Emanuelson, E. Grosse Beilage, K. D. C. Stärk, and J. Dewulf. 2016. The biosecurity status and its associations with production and management characteristics in farrow-to-finish pig herds. Animal 10:478–489. doi:10.1017/S1751731115002487. 26567800

[CIT0297] Postma, M., K. D. C. Stärk, M. Sjölund, A. Backhans, E. Grosse, S. Beilage, C. Lösken, C. Belloc, L. D. Collineau, D. Iten, et al. 2015. Alternatives to the use of antimicrobial agents in pig production: a multi-country expert-ranking of perceived effectiveness, feasibility and return on investment. Prev. Veter. Med. 118:457–466. doi:10.1016/j.prevetmed.2015.01.010. 25650306

[CIT0298] Price, L. B., E. Johnson, R. Vailes, and E. Silbergeld. 2005. Fluoroquinolone-resistant campylobacter isolates from conventional and antibiotic-free chicken products. Environ. Health Perspect. 113:557–560. doi:10.1289/ehp.7647. 15866763 PMC1257547

[CIT0299] Quesnel, H., C. Farmer, and N. Devillers. 2012. Colostrum intake: influence on piglet performance and factors of variation. Livest. Prod. Sci. 146:105–114. doi:10.1016/j.livsci.2012.03.010.

[CIT0300] Raasch, S. M., M. Posta, J. Dewulf, K. D. C. Stärk, E. Grosse, and S. Beilage. 2018. Association between antimicrobial usage, biosecurity measures as well as farm performance in German farrow-to-finish farms. Porcine Health Mgmt. 4:30. doi:10.1186/s40813-018-0106-5. PMC6293545 30564434

[CIT0301] Radcliffe, J. S., Z. Zhang, and E. T. Kornegay. 1998. The effects of microbial phytase, citric acid, and their interaction in a corn-soybean meal-based diet for weanling pigs. J. Anim. Sci. 76:1880–1886. doi:10.2527/1998.7671880x. 9690644

[CIT0302] Rakhshandeh, A., J. K. Htoo, N. Karrow, S. P. Miller, and C. F. M. de Lange. 2014. Impact of immune system stimulation on ileal nutrient digestibility and utilization of methionine plus cysteine intake for whole body protein deposition in growing pigs. Brit. J. Nutr. 111:101–110. doi:10.1017/S0007114513001955. 23803219

[CIT0303] Ramirez, A., and P. Zaabel. 2012. Swine biological risk management. Veterinary Diagnostic and Production Animal Medicine Publications. 3. doi:lib.dr.iastate.edu/vdpam_pubs/3.

[CIT0304] Regassa, A., and C. M. Nyachoti. 2018. Application of resistant starch in swine and poultry diets with particular reference to gut health and function. Anim. Nutr 4:305–310. doi:10.1016/j.aninu.2018.04.001. 30175259 PMC6116817

[CIT0305] Ren, P., F. Almeida, U. Orlando, M. Gonçalves, D. Hancock, and M. Vazquez-Añón. 2021. Optimal standardized ileal digestible total sulfur amino acids to lysine requirements are increased in nursery pigs raised under antibiotic-free feeding regime. Animals 11:3143. doi:10.3390/ani11113143. 34827875 PMC8614517

[CIT0306] Renaudeau, D. J., J. L. Gourdine, B. A. N. Silva, and J. Noblet. 2008. Nutritional routes to attenuate heat stress in pigs. Hammamet (Tunisia): Proc. Int’l Conf. Livestock Global Climate Change. Brit. Soc. Anim. Sci. p. 134–138.

[CIT0307] Rezaei, R., W. Wang, Z. Dai, J. Wang, and G. Wu. 2013. Biochemical and physiological bases for utilization of dietary amino acids by young pigs. J. Anim. Sci. Biotechnol. 4:1. doi:10.1186/2049-1891-4-7. 23445937 PMC3599606

[CIT0308] Robert, S., D. M. Weary, and H. Gonyou. 1999. Segregated early weaning and welfare of piglets. J. Appl. Anim. Welfare Sci. 2:31–40. doi: 10.1207/s15327604jaws0201_3. 16363960

[CIT0309] Rocha-Mendoza, D., E. Kosmerl, A. Krentz, L. Zhang, S. Badiger, G. Miyagusuku-Cruzado, A. Mayta-Apaza, M. Giusti, R. Jimenez-Flores, and I. Garcia-Cano. 2021. Invited review: acid whey trends and health benefits. J. Dairy Sci. 104:1262–1275. doi:10.3168/jds.2020-19038. 33358165

[CIT0310] Rodrigues, L. A., M. O. Wellington, J. C. González-Vega, J. H. K. Htoo, A. G. Van Kessel, and D. A. Columbus. 2021. Functional amino acid supplementation, regardless of dietary protein content, improves growth performance and immune status of weaned pigs challenged with *Salmonella Typhimurium*. J. Anim. Sci. 99:skaa365. doi:10.1093/jas/skaa365. 33529342 PMC8631066

[CIT0311] Rosa, L., D. D. Chiarelli, M. C. Rulli, J. Dell’Angelo, and P. D’Odorico. 2020. Global agricultural economic water scarcity. Sci. Adv. 6:eaaz6031. doi:10.1126/sciadv.aaz6031. 32494678 PMC7190309

[CIT0312] Rose, N., and M. Andraud. 2017. The use of vaccines to control pathogen spread in pig populations. Porc. Health Manag. 3:8. doi: 10.1186/s40813-017-0053-6. PMC5382368 28405464

[CIT0313] Roselli, M., A. Finamore, M. S. Britti, P. Bosi, I. Oswald, and E. Mengheri. 2005. Alternatives to in-feed antibiotics in pigs: evaluation of probiotics, zinc or organic acids as protective agents for the intestinal mucosa. A comparison of in vitro and in vivo results. Anim. Res. 54:203–218. doi:10.1051/animres:2005012.

[CIT0314] Rosen, B. P. 2002. Transport and detoxification systems for transition metals, heavy metals and metalloids in eukaryotic and prokaryotic microbes. Comp. Biochem. Physiol. A: Mol. Integr. Physiol. 133:689–693. doi:10.1016/S1095-6433(02)00201-5. 12443926

[CIT0315] Rossi, R., G. Pastorelli, S. Cannata, and C. Corino. 2020. Recent advances in the use of fatty acids as supplements in pig diets: a review. Anim. Feed Sci. Technol. 162:1–11. doi: 10.1016/j.anifeedsci.2010.08.013.

[CIT0316] Roura, E., and M. Fu. 2017. Taste, nutrient sensing and feed intake in pigs (130 years of research: then, now and future). Anim. Feed Sci. Technol. 233:3–12. doi:10.1016/j.anifeedsci.2017.08.002.

[CIT0317] Ruckman, L. A., A. L. Petry, S. A. Gould, and J. F. Patience. 2020. The impact of porcine spray-dried plasma protein and dried egg protein harvested from hyper-immunized hens, provided in the presence or absence of subtherapeutic levels of antibiotics in the feed, on growth and indicators of intestinal function and physiology of nursery pigs. Transl. Anim. Sci 4:txaa095. doi:10.1093/tas/txaa095. 32844150 PMC7438620

[CIT0318] Scheidt, A. B., T. R. Cline, L. K. Clark, V. B. Mayrose, W. G. van Alstine, M. A. Diekman, and W. L. Singleton. 1995. The effect of all-in-all-out growing-finishing on the health of pigs. J. Swine Health Prod. 3:202–205.

[CIT0319] Schlimme, E., D. Martin, and H. Meisel. 2000. Nucleosides and nucleotides: natural bioactive substances in milk and colostrum. Brit. J. Nutr 84:59–68. doi:10.1017/S0007114500002269. 11242448

[CIT0320] Schmolke, S. A., Y. Z. Li, and H. W. Gonyou. 2003. Effect of group size on performance of growing-finishing pigs. J. Anim. Sci. 81:874–878. doi:10.2527/2003.814874x. 12723074

[CIT0321] Schweer, W. A., A. Ramirez, and N. Gabler. 2017. Alternatives to in-feed antibiotics for nursery pigs. Puerto Vallarta (Mexico): Proc. Mex. Assoc. Spec. Anim. Nutr. p. 6.

[CIT0322] Scipioni, R., G. Martelli, and L. A. Volpelli. 2009. Assessment of welfare in pigs, Italian J. Anim. Sci. 8:117–137. doi:10.4081/ijas.2009.s1.117.

[CIT0323] Shridhar, P. B., R. G. Amachawadi, M. Tokach, I. Patel, J. Gangiredla, M. Mammel, and T. G. Nagaraja. 2022. Whole genome sequence analyses-based assessment of virulence potential and antimicrobial susceptibilities and resistance of *Enterococcus faecium* strains isolated from commercial swine and cattle probiotic products. J. Anim. Sci. 100:skac030. doi: 10.1093/jas/skac030. 35150575 PMC8908542

[CIT0324] da Silva, B. L., M. P. Abuçafy, E. Berbel Manaia, J. A. Oshiro Junior, B. G. Chiari-Andréo, R. Pietro, and L. A. Chiavacci. 2019. Relationship between structure and antimicrobial activity of zinc oxide nanoparticles: an overview. Int. J. Nanomedicine 14:9395–9410. doi:10.2147/IJN.S216204. 31819439 PMC6897062

[CIT0325] Silva, G. S., L. G. Corbellini, D. L. Linhares, K. L. Baker, and D. J. Holtkamp. 2018. Development and validation of a scoring system to assess the relative vulnerability of swine breeding herds to the introduction of PRRS virus. Prev. Vet. Med. 160:116–122. doi:10.1016/j.prevetmed.2018.10.004. 30388993

[CIT0326] Skrovanek, S. K., R. DiGuilio, W. Bailey, R. Huntington, B. Urbas, G. Mayilvaganan, G. Mercogliano, and J. M. Mullin. 2014. Zinc and gastrointestinal disease. World J. Gastrointest. Pathophysiol 5:496–513. doi:10.4291/wjgp.v5.i4.496. 25400994 PMC4231515

[CIT0327] Slifierz, M. J., R. M. Friendship, and J. S. Weese. 2015. Methicillin-resistant staphylococcus aureus in commercial swine herds is associated with disinfectant and zinc usage. Appl. Environ. Microbiol. 81:2690–2695. doi:10.1128/AEM.00036-15. 25662976 PMC4375337

[CIT0328] Smith, B. N., and R. N. Dilger. 2018. Immunomodulatory potential of dietary soybean-derived isoflavones and saponins in pigs. J. Anim. Sci. 96:1288–1304. doi:10.1093/jas/sky036. 29471443 PMC6140853

[CIT0329] Smith, F., J. E. Clark, B. L. Overman, C. C. Tozel, J. H. Huang, J. E. F. Rivier, A. T. Blisklager, and A. J. Moeser. 2010. Early weaning stress impairs development of mucosal barrier function in the porcine intestine. Amer. J. Physiol. - Gastrointestinal and Liver Physiol. 298:G352–G363. doi: 10.1152/ajpgi.00081.2009. PMC2838512 19926814

[CIT0330] Smith, L. F., J. F. Patience, H. W. Gonyou, A. D. Beaulieu, and R. D. Boyd. 2004. The impact of feeder adjustment and group size/floor space allowance on the performance of nursery pigs. J. Swine Health Prod. 12:111–118.

[CIT0331] Sneeringer, S., M. Bowman, and M. Clancy. 2019. The U.S. and EU Animal Pharmaceutical Industries in the Age of Antibiotic Resistance, ERR-264. Washington (DC): U.S. Department of Agriculture, Economic Research Service.

[CIT0332] Spoolder, H. A. M., S. A. Edwards, and S. Corning. 1999. Effects of group size and feeder space allowance on welfare in finishing pigs. Anim. Sci. 69:481–489. doi:10.1017/S135772980005133X.

[CIT0333] Spreeuwenberg, M. A. M., J. M. A. J. Verdonk, H. R. Gaskins, and M. W. A. Verstegen. 2001. Small intestine epithelial barrier function is compromised in pigs with low feed intake at weaning. J. Nutr. 131:1520–1527. doi:10.1093/jn/131.5.1520. 11340110

[CIT0334] Spring, P., C. Wenk, A. Connolly, and A. Kiers. 2015. A review of 733 published trials on Bio-Mos®, a mannan oligosaccharide, and Actigen®, a second generation mannose rich fraction, on farm and companion animals. J. Appl. Anim. Nutr 3:E8. doi:10.1017/jan.2015.6.

[CIT0335] Stark, C. R. 2012. Feed processing to maximize feed efficiency. In: J. F. Patience, editor, Feed efficiency in swine. Wageningen (The Netherlands): Wageningen Academic Press. p. 131–151 doi:10.3921/978-90-8686-756-1.

[CIT0336] Starr, M. P., and D. M. Reynolds. 1951. Streptomycin resistance of coliform bacteria from turkeys fed streptomycin. Amer. J. Publ. Health 41:1375–1380. 10.2105/ajph.41.11_pt_1.1375 PMC1525794 14885532

[CIT0337] Stephens, D. B., D. L. Ingram, and D. F. Sharman. 1983. An investigation into some cerebral mechanisms involved in schedule-induced drinking in the pig. Quart. J. Exp. Physiol 68:653–660. doi:10.1113/expphysiol.1983.sp002755. 6647740

[CIT0338] Stinn, J. P. and H. Xin. 2014. Performance of evaporative cooling pads on a swine farm in central Iowa. ASL R2932. Ames (IA): Anim. Ind. Rpt., Iowa State University.

[CIT0339] Stoian, A., J. Zimmerman, J. Ji, T. J. Hefley, S. Dee, D. G. Diel, R. Rowland, and M. C. Niederwerder. 2019. Half-life of African Swine Fever virus in shipped feed. Emerg. Infect. Dis. 25:2261–2263. doi:10.3201/eid2512.191002. 31524583 PMC6874236

[CIT0340] Street, B. R., and H. W. Gonyou. 2008. Effects of housing finishing pigs in two group sizes and at two floor space allocations on production, health, behavior, and physiological variables. J. Anim. Sci. 86:982–991. doi:10.2527/jas.2007-0449. 17965323

[CIT0341] Suiryanrayna, M. V. A. N., and J. V. Ramana. 2015. A review of the effects of dietary organic acids fed to swine. J. Anim. Sci. Biotechnol. 6:45. doi:10.1186/s40104-015-0042-z. 26500769 PMC4618844

[CIT0342] Sulabo, R. C., M. D. Tokach, J. M. DeRouchey, S. S. Dritz, R. D. Goodband, and J. L. Nelssen. 2010a. Influence of feed flavors and nursery diet complexity on preweaning and nursery pig performance. J. Anim. Sci. 88:3918–3926. doi:10.2527/jas.2009-2724. 20833770

[CIT0343] Sulabo, R. C., M. D. Tokach, S. S. Dritz, R. D. Goodband, J. M. DeRouchey, and J. L. Nelssen. 2010b. Effects of varying creep feeding duration on the proportion of pigs consuming creep feed and neonatal pig performance. J. Anim. Sci. 88:3154–3162. doi:10.2527/jas.2009-2134. 20495115

[CIT0344] Sulakvelidze, A. A., A. Alavidze, and J. G. Morris, Jr. 2001. Bacteriophage therapy. Antimicrob. Agents Chemother. 45:649–659. doi:10.1128/AAC.45.3.649-659.2001. 11181338 PMC90351

[CIT0345] Suleiman, R. A., K. A. Rosentrater, and C. J. Bern. 2013. Effects of deterioration parameters on storage of maize: a review. J. Nat. Sci. Res. 3:147–165. doi:10.1016/j.jspr.2019.101566.

[CIT0346] Tan, F. P. Y., E. Beltranena, and R. T. Zijlstra. 2021. Resistant starch: implications of dietary inclusion on gut health and growth in pigs: a review. J. Animal Sci. Biotechnol. 12:124. doi:10.1186/s40104-021-00644-5. PMC8597317 34784962

[CIT0347] Tang, K. L., N. P. Caffrey, D. B. Nobrega, S. C. Cork, P. E. Ronksley, H. W. Barkema, A. J. Polachek, H. Ganshorn, N. Sharma, J. D. Kellner, et al. 2017. Restricting the use of antibiotics in food-producing animals and its association with antibiotic resistance in food-producing animals and human beings: a systemic review and meta-analysis. Lancet Planet Health 1:316–327. doi:10.1016/S2542-5196(17)30141-9. PMC5785333 29387833

[CIT0348] Tang, K. L., N. P. Caffrey, D. B. Nóbrega, S. C. Cork, P. E. Ronksley, H. W. Barkema, A. J. Polachek, H. Ganshorn, N. Sharma, J. D. Kellner, S. L. Checkley, and W.A. Ghali, 2019. Examination of unintended consequences of antibiotic use restrictions in food-producing animals: sub-analysis of a systematic review. One Health 7:100095. doi: 10.1016/j.onehlt.2019.100095. 31193679 PMC6538949

[CIT0349] Taylor-Pickard, J., T. McArdle, and S. Icely. 2017. Effect of feeding Actigen™ to sows during gestation and lactation and on piglet performance. J. Appl. Anim. Nutr 5:E1. doi:10.1017/Jan.2017.2.

[CIT0350] Teillant, A., and R. Laxminarayan. 2015. Economics of antibiotic use in U.S. Swine and poultry production. Choices 30:1–11.

[CIT0351] Terpstra, C., G. Wensvoort, and J. M. A. Pol. 1991. Experimental reproduction of porcine epidemic abortion and respiratory syndrome (mystery swine disease) by infection with Lelystad virus: Koch’s postulates fulfilled. Vet. Q 13:131–136. doi:10.1080/01652176.1991.9694297. 1949539

[CIT0352] Thanki, A. M., S. Hooton, A. M. Gigante, R. J. Atterbury, and M. R. J. Clokie. 2021. Potential roles for bacteriophages in reducing *Salmonella* from poultry and swine. In: A. Lamas, P. Regal, and C. M. Franco, editors, *Salmonella* spp. - a global challenge. IntechOpen. doi: 10.5772/intechopen.96984.

[CIT0353] Thomas, L. L., R. D. Goodband, J. C. Woodworth, M. D. Tokach, J. M. DeRouchey, and S. S. Dritz. 2017. Effects of space allocation on finishing pig growth performance and carcass characteristics. Transl. Anim. Sci 1:351–357. doi:10.2527/tas2017.0042. 32704659 PMC7205350

[CIT0354] Thomas, R. R., P. R. Locke, A. Karriker, A. Ramirez, J. Zhang, J. S. Ellingson, K. K. Crawford, J. L. Bates, K. J. Hammen, and D. J. Holtkamp. 2015. Evaluation of time and temperature sufficient to inactivate porcine epidemic diarrhea virus in swine feces on metal surfaces. J. Swine Health Prod. 23:84–90.

[CIT0355] Thomke, S., and K. Elwinger. 1998. Growth promotants in feeding pigs and poultry. II. Mode of action of antibiotic growth promotants. Ann. Zootech. 47:153–167.

[CIT0356] Tiwari, U. P., H. Chen, S. W. Kim, and R. Jha. 2018. Supplemental effect of xylanase and mannanase on nutrient digestibility and gut health of nursery pigs studied using both in vivo and in vitro models. Anim. Feed Sci. Technol. 245:77–90. doi:10.1016/j.anifeedsci.2018.07.002.

[CIT0357] Tizard, I. R. 2021. Porcine vaccines. Vaccines for Veterinarians 225–242.e1. doi:10.1016/B978-0-323-68299-2.00027-7.

[CIT0358] Tokach, M.D., H. S. Cemin, R. C. Sulabo, and R. D. Goodband. 2020. Feeding the suckling pig: creep feeding. In: C. Farmer, editor, The suckling and weaned pig. Wageningen (The Netherlands): Wageningen Academic Publishers. p. 139–158.

[CIT0359] Tokach, M. D., J. E. Pettigrew, L. J. Johnston, M. Øverland, J. W. Rust, and S. G. Cornelius. 1995. Effect of adding fat and (or) milk products to the weanling pig diet on performance in the nursery and subsequent grow-finish stages. J. Anim. Sci. 73:3358–3368. doi:10.2527/1995.73113358x. 8586595

[CIT0360] Torrallardona, D. 2010. Spray dried animal plasma as an alternative to antibiotics in weanling pigs - a review. Anim. Biosci 23:131–148. doi:10.5713/ajas.2010.70630.

[CIT0361] Torrey, S., and E. L. M. Toth. 2008. Effect of drinker type on water intake and waste in newly weaned piglets. J. Anim. Sci. 86:1439–1445. doi:10.2527/jas.2007-0632. 18272851

[CIT0362] Trachsel, J., C. Briggs, N. K. Gabler, H. K. Allen, and C. L. Loving. 2019. Dietary resistant potato starch alters intestinal microbial communities and their metabolites, and markers of immune regulation and barrier function in swine. Front. Immunol. 10:1381. doi:10.3389/fimmu.2019.01381. 31275319 PMC6593117

[CIT0363] Trowell, H. C., D. A. T. Southgate, T. M. S. Wolever, A. R. Leeds, M. A. Gassull, and D. J. A. Jenkins. 1976. Dietary fiber redefined. Lancet 307:967. 10.1016/s0140-6736(76)92750-1 57372

[CIT0364] Trudeau, M. P., H. Verma, F. Sampedro, P. E. Urriola, G. C. Shurson, J. McKelvey, S. D. Pillai, and S. M. Goyal. 2016. Comparison of thermal and non-thermal processing of swine feed and the use of selected feed additives on inactivation of porcine epidemic diarrhea virus (PEDV). PLoS One 11:e0158128. doi:10.1371/journal.pone.0158128. 27341670 PMC4920390

[CIT0365] Tugnoli, B., G. Giovagnoni, A. Piva, and E. Grilli. 2020. From acidifiers to intestinal health enhancers: how organic acids can improve growth efficiency of pigs. Animals 10:134. doi:10.3390/ani10010134. PMC7022919 31947627

[CIT0366] Upadhaya, S., K. Lee, and I. Kim. 2014. Protected organic acid blends as an alternative to antibiotics in finishing pigs. Anim. Biosci 27:1600–1607. doi:10.5713/ajas.2014.14356. PMC4213705 25358320

[CIT0367] Vahjen, W., D. Pietruszynska, I. C. Starke, and J. Zentek. 2015. High dietary zinc supplementation increases the occurrence of tetracycline and sulfonamide resistance genes in the intestine of weaned pigs. Gut Pathog 7:23–28. doi:10.1186/s13099-015-0071-3. 26322131 PMC4551370

[CIT0368] Van Barneveld, R., E. Batterham, and B. Norton. 1994. The effect of heat on amino acids for growing pigs. Brit. J. Nutr. 72:221–241. doi:10.1079/BJN19940026. 7947642

[CIT0369] Van Belleghem, J. D., K. Dąbrowska, K. M. Vaneechoutte, J. J. Barr, and P. L. Bollyky. 2019. Interactions between bacteriophage, bacteria, and the mammalian immune system. Viruses 11:10. doi:10.3390/v11010010. PMC6356784 30585199

[CIT0370] Vande Pol, K. D., R. O. Bautista, H. Harper, C. M. Shull, C. B. Brown, and M. Ellis. 2021. Effect of within-litter birth weight variation after cross-fostering on piglet pre-weaning growth and mortality. Transl. Anim. Scitxab039. doi: 10.1093/tas/txab039. 34723136 PMC8552483

[CIT0371] Van Soest, P. J., and R. W. McQueen. 1973. The chemistry and estimation of fibre. Proc. Nutr. Soc. 32:123–130. doi:10.1079/PNS19730029. 4605228

[CIT0372] Van Soest, P. J., J. B. Robertson, and B. A. Lewis. 1991. Methods for dietary fiber, neutral detergent fiber, and nonstarch polysaccharides in relation to animal nutrition. J. Dairy Sci. 74:3583–3597. doi:10.3168/jds.S0022-0302(91)78551-2. 1660498

[CIT0373] Villanueva-García, D., D. Mota-Rojas, J. Martínez-Burnes, A. Olmos-Hernández, P. Mora-Medina, C. Salmerón, J. Gómez, L. Boscato, O. Gutiérrez-Pérez, V. Cruz, et al. 2020. Hypothermia in newly born piglets: mechanisms of thermoregulation and pathophysiology of death. J. Anim. Behav. Biometeorol. 9:2101. doi: 10.31893/jabb.21001.

[CIT0374] Visek, W. J. 1978. The mode of growth promotion by antibiotics. J. Anim. Sci. 46:1447–1469. doi:10.2527/jas1978.4651447x.

[CIT0375] Vukmirović, D., R. Čolović, S. Rakita, T. Brlek, O. Đuragić, and D. Solà-Oriol. 2017. Importance of feed structure (particle size) and feed form (mash vs. pellets) in pig nutrition–A review. Anim. Feed Sci. Technol. 233:133–144. doi:10.1016/j.anifeedsci.2017.06.016.

[CIT0376] Wathes, C. M., T. G. M. Demmers, N. Teer, R. P. White, L. L. Taylor, V. Bland, P. Jones, D. Armstrong, A. C. J. Gresham, J. Hartung, et al. 2004. Production responses of weaned pigs after chronic exposure to airborne dust and ammonia. Anim. Sci. 78:87–97. doi:10.1017/S135772980005387X.

[CIT0377] Wastell, M. E., C. A. P. Garbossa, and A. P. Schinckel. 2018. Effects of wet/dry feeder and pen stocking density on grow-finish pig performance. Transl. Anim. Sci 2:358–364. doi:10.1093/tas/txy073. 32704718 PMC7200493

[CIT0378] Weber, E. K., J. F. Patience, and K. J. Stalder. 2015. Wean-to-finish feeder space availability effects on nursery and finishing pig performance and total tract digestibility in a commercial setting when feeding dried distillers grains with solubles. J. Anim. Sci. 93:1905–1915. doi:10.2527/jas.2014-8136. 26020213

[CIT0379] Wei, X., K. A. Bottoms, H. H. Stein, L. Blavi, C. L. Bradley, J. Bergstrom, J. Knapp, R. Story, C. Maxwell, T. Tsai, et al. 2021a. Dietary organic acids modulate gut microbiota and improve growth performance of nursery pigs. Microorganisms 9:110. doi:10.3390/microorganisms9010110. PMC7824888 33466376

[CIT0380] Wei, X., T. Tsai, S. Howe, and J. Zhao. 2021b. Weaning induced gut dysfunction and nutritional interventions in nursery pigs: a partial review. Animals 5:1279. doi:10.3390/ani11051279. PMC8146462 33946901

[CIT0381] Weinbauer, M. G. 2004. Ecology of prokaryotic viruses. FEMS Microbiol. Rev. 28:127–181. doi:10.1016/j.femsre.2003.08.001. 15109783

[CIT0382] Wellock, I. J., G. C. Emmans, and I. Kyriazakis. 2003. Predicting the consequences of social stressors on pig food intake and performance. J. Anim. Sci. 81:2995–3007. doi:10.2527/2003.81122995x. 14677855

[CIT0383] Weng, R. C. 2016. Dietary fat preference and effects on performance of piglets at weaning. Asian-Austral. J. Anim. Sci. 30:834–842. doi:10.5713/ajas.16.0499. PMC5411847 27739293

[CIT0384] Wensley, M. R., M. D. Tokach, J. C. Woodworth, R. D. Goodband, J. T. Gebhardt, J. M. DeRouchey, and D. McKilligan. 2021a. Maintaining continuity of nutrient intake after weaning II: review of pre-weaning strategies. Transl. Anim. Sci 5:txab021. doi:10.1093/tas/txab021. 33750992 PMC7963027

[CIT0385] Wensley, M. R., M. D. Tokach, J. C. Woodworth, R. D. Goodband, J. T. Gebhardt, J. M. DeRouchey, and D. McKilligan. 2021b. Maintaining continuity of nutrient intake after weaning II: review of post-weaning strategies. Transl. Anim. Sci 5:txab022. doi:10.1093/tas/txab022. 34841202 PMC8611789

[CIT0386] Wensvoort, C., C. Terpstra, J. M. A. Pal, E. A. ter Laak, M. Bloemraad, E. P. de Kluyver, C. Kragten, L. van Buiten, A. den Besten, F. Wagenaa., J. M. Broekhuijaen, P. L. J. M. Moonen, T. Zetstra, E. A. de Boer, H. J. Tibben, M. F. de Jong, P. van’t Veld, G. J. R. Groenland, J. A. van Gennep, M. T. Voets, J. H. M. Verheijden, and J. Braamsknmp. 1991. Mystery swine disease in the netherlands: the isolation of lelystad virus. Vet. Q 13:121–130. doi:10.1080/01652176.1991.9694296. 1835211

[CIT0387] Wessels, A. G., T. Chalvon-Demersey, and J. Zentek. 2021. Use of low dosage amino acid blends to prevent stress-related piglet diarrhea. Transl. Anim. Sci 5:txab209. doi:10.1093/tas/txab209. 34805771 PMC8599283

[CIT0388] Whitworth, K. M., and R. S. Prather. 2017. Gene editing as applied to prevention of reproductive porcine reproductive and respiratory syndrome. Mol. Reprod. Dev. 84:926–933. doi:10.1002/mrd.22811. 28390179

[CIT0389] Widowski, T. M., T. Cottrell, C. E. Dewey, and R. M. Friendship. 2003. Observations of piglet-directed behavior patterns and skin lesions in eleven commercial swine herds. J. Swine Health Prod. 11:181–185.

[CIT0390] Wiedemann, V. E., R. Linckh, P. Kühlmann, P. Schmidt, and U. Lösch. 1991. Chicken egg antibodies for prophylaxis and therapy of infectious intestinal diseases. J. Vet. Med. B 38:283–291. doi:10.1111/j.1439-0450.1991.tb00872.x. 1887700

[CIT0391] Wierup, M. 2001. The Swedish experience of the 1986 year ban of antimicrobial growth promoters, with special reference to animal health, disease prevention, productivity, and usage of antimicrobials. Microb. Drug Resist. 7:183–190. doi:10.1089/10766290152045066. 11442345

[CIT0392] Wilberts, B. L., P. H. Arruda, J. M. Kinyon, T. S. Frana, C. Wang, D. R. Magstadt, D. R. Madson, J. F. Patience, and E. R. Burrough. 2014. Investigation of the impact of increased dietary insoluble fiber through the feeding of distillers dried grains with solubles (DDGS) on the incidence and severity of *Brachyspira-*associated colitis in pigs. PLoS One 9:e114741. doi:10.1371/journal.pone.0114741. 25485776 PMC4259391

[CIT0393] Wilson, N., Y. Li, M. Rajput, K. Thelen, K. Kerr, and A. J. Moeser. 2019. Weaning and postnatal age influence the early time course and nature of intestinal mast cell activation and mucosal inflammation in a porcine model of early life adversity. J. Immunol. 202(1 Suppl.):73.11.

[CIT0394] Windisch, W. K., K. Schedle, C. Plitzner, and A. Kroismayr. 2008. Use of phytogenic products as feed additives for swine and poultry. J. Anim. Sci. 86:E140–E148. doi:10.2527/jas.2007-0459. 18073277

[CIT0395] Wolter, B. and A. Gaines. 2016. Growth of a pig production business, consumer challenges, strategies and opportunities. Proc. Leman Conference, cited by C. Johnson.

[CIT0396] Wolter, B. F., M. Ellis, S. E. Curtis, N. R. Augspurger, D. N. Hamilton, E. N. Parr, and D. M. Webel. 2001. Effect of group size on pig performance in a wean-to-finish production system. J. Anim. Sci. 79:1067–1073. doi:10.2527/2001.7951067x. 11374526

[CIT0397] Wolter, B. F., M. Ellis, S. E. Curtis, E. N. Parr, and D. M. Webel. 2000. Group size and floor-space allowance can affect weanling-pig performance. J. Anim. Sci. 78:2062–2067. doi:10.2527/2000.7882062x. 10947088

[CIT0398] Wu, G. F., W. Bazer, G. A. Johnson, D. A. Knabe, R. C. Burghardt, T. E. Spencer, X. L. Li, and G. J. Wang. 2011. Important roles for l-glutamine in swine nutrition and production. J. Anim. Sci. 89:2017–2030. doi:10.2527/jas.2010-3614. 21169511

[CIT0399] Xiu, Z., Y. Liu, J. Mathieu, J. Wang, D. Zhu, and P. J. Alvarez. 2014. Elucidating the genetic basis for *Escherichia coli* defense against silver toxicity using mutant arrays. Environ. Toxicol. Chem. 33:993–997. doi:10.1002/etc.2514. 24408659

[CIT0400] Zeng, Z., S. Zhang, H. Wang, and X. Piao. 2015. Essential oil and aromatic plants as feed additives in non-ruminant nutrition: a review. J. Anim. Sci. Biotechnol. 6:7. doi:10.1186/s40104-015-0004-5. 25774291 PMC4359495

[CIT0401] Zentek, J., S. Buchheit-Renko, F. Ferrara, W. Vahjen, A. G. Van Kessel, and R. Pieper. 2011. Nutritional and physiological role of medium-chain triglycerides and medium-chain fatty acids in piglets. Anim. Health Res. Rev. 12:83–93. doi:10.1017/S1466252311000089. 21676342

[CIT0402] Zentek, J., F. Ferrara, R. Pieper, L. Tedin, W. Meyer, and W. Vahjen. 2013. Effects of dietary combinations of organic acids and medium chain fatty acids on the gastrointestinal microbial ecology and bacterial metabolites in the digestive tract of weaning piglets. J. Anim. Sci. 91:3200–3210. doi:10.2527/jas.2012-5673. 23798515

[CIT0403] Zhao, J., Z. Zhang, S. Zhang, G. Page, and N. W. Jaworski. 2021. The role of lactose in weanling pig nutrition: a literature and meta-analysis review. J. Anim. Sci. Biotechnol. 12:10. doi:10.1186/s40104-020-00522-6. 33431022 PMC7798279

[CIT0404] Zheng, L., D. Li, Z. -L. Li, L. -N. Kang, Y. -Y. Jiang, X. -Y. Liu, Y. -P. Chi, Y. -Q. Li, and J. -H. Wang. 2017. Effects of Bacillus fermentation on the protein microstructure and anti‐nutritional factors of soybean meal. Lett. Appl. Microbiol. 65:520–526. doi:10.1111/lam.12806. 28975646

[CIT0405] Zhu, H., C. A. Hart, D. Sales, and N. B. Roberts. 2006. Bacterial killing in gastric juice – effect of pH and pepsin on *Escherichia coli* and *Helicobacter pylori*. J. Med. Microbiol. 55:1265–1270. doi:10.1099/jmm.0.46611-0. 16914658

[CIT0406] Zijlstra, R. T., C. F. M. de Lange, and J. F. Patience. 1999. Nutritional value of wheat samples for growing pigs: chemical composition and digestible energy content. Can. J. Anim. Sci. 79:187–194. doi:10.4141/A98-103.

[CIT0407] Zou, L. J., X. Xiong, H. N. Liu, J. Zhou, Y. H. Liu, and Y. L. Yin. 2019. Effects of dietary lysozyme levels on growth performance, intestinal morphology, immunity response and microbiota community of growing pigs. J. Sci. Food Agric. 99:1643–1650. doi:10.1002/jsfa.9348. 30198063

